# Hydrophilic Poly(meth)acrylates by Controlled Radical Branching Polymerization: Hyperbranching and Fragmentation

**DOI:** 10.1021/acs.macromol.4c00408

**Published:** 2024-05-29

**Authors:** Kriti Kapil, Arman Moini Jazani, Julian Sobieski, Leticia P. Madureira, Grzegorz Szczepaniak, Michael R. Martinez, Adam Gorczyński, Hironobu Murata, Tomasz Kowalewski, Krzysztof Matyjaszewski

**Affiliations:** † Department of Chemistry, Carnegie Mellon University, 4400 Fifth Avenue, Pittsburgh, Pennsylvania 15213, United States; ‡ Faculty of Chemistry, University of Warsaw, Pasteura 1, Warsaw 02-093, Poland; § PPG Industries, Inc., 4325 Rosanna Drive, Allison Park, Pennysylvania 15101, United States; ∥ Faculty of Chemistry, Adam Mickiewicz University, Uniwersytetu Poznańskiego 8, Poznań, 61-614, Poland

## Abstract

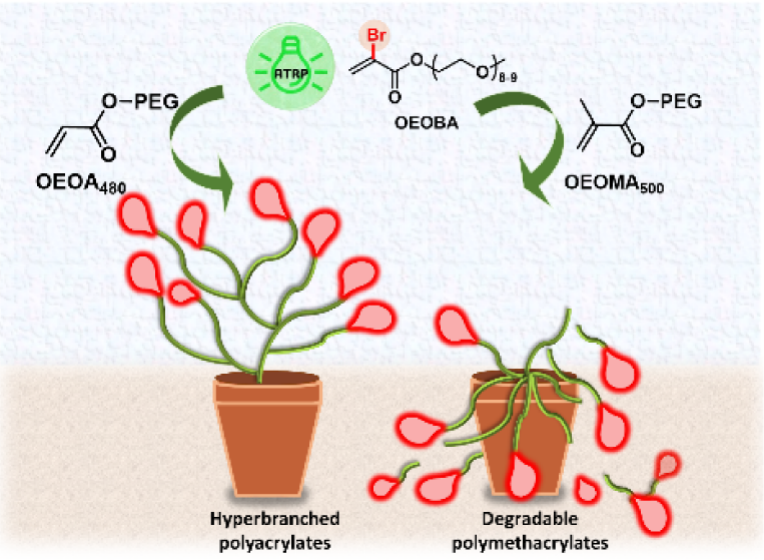

Topology significantly impacts polymer properties and applications. Hyperbranched polymers (HBPs) synthesized via atom transfer radical polymerization (ATRP) using inimers typically exhibit broad molecular weight distributions and limited control over branching. Alternatively, copolymerization of inibramers (IB), such as α-chloro/bromo acrylates with vinyl monomers, yields HBPs with precise and uniform branching. Herein, we described the synthesis of hydrophilic HB polyacrylates in water by copolymerizing a water-soluble IB, oligo(ethylene oxide) methyl ether 2-bromoacrylate (OEOBA), with various hydrophilic acrylate comonomers. Visible-light-mediated controlled radical branching polymerization (CRBP) with dual catalysis using eosin Y (EY) and copper complexes resulted in HBPs with various molecular weights (*M*_n_ = 38 000 to 170 000) and degrees of branching (2%–24%). Furthermore, the optimized conditions enabled the successful application of the OEOBA to synthesize linear-hyperbranched block copolymers and hyperbranched polymer protein hybrids (HB-PPH), demonstrating its potential to advance the synthesis of complex macromolecular architecture under environmentally benign conditions. Copolymerization of hydrophilic methacrylate monomer, oligo(ethylene oxide) methyl ether methacrylate (OEOMA_500_), and inibramer OEOBA was accompanied by fragmentation via β-carbon C–C bond scission and subsequent growth of polymer chains from the fragments. Furthermore, computational studies investigating the fragmentation depending on the IB and comonomer structure supported the experimental observations. This work expands the toolkit of water-soluble inibramers for CRBP and highlights the critical influence of the inibramer structure on reaction outcomes.

## Introduction

Reversible deactivation radical polymerization (RDRP), also known as controlled radical polymerization (CRP), has received significant attention over the past few decades as it enables the precise synthesis of macromolecules with predetermined molecular weight, narrow molecular weight distribution, controlled topology, composition, and functionality.^1−9^ Notably, polymer topology is a crucial attribute that impacts the solution/melt viscosity, glass transition temperature, solubility in diverse solvents, and size of individual polymer chains in solution.^10−16^ RDRP facilitates the development of advanced functional materials with well-defined architecture, including densely grafted linear copolymers,^17^ star copolymers,^18^ bottlebrush copolymers,^19,20^ hyperbranched polymers,^21−23^ networks/gels,^24,25^ and cyclic copolymers,^26^ suitable for an extensive range of applications such as nanocomposites,^27^ and biomedical uses, including drug delivery systems^28,29^ and scaffolds.^30−32^

Structurally controlled hyperbranched polymers (HBPs) exhibit distinctive physical properties, such as weaker entanglements, lower intrinsic viscosity, multiple modifiable end-groups, and the presence of cavities.^33,34^ Consequently, HBPs emerge as the most promising alternatives to dendrimers, circumventing the tedious multistep synthesis.^35,36^ However, achieving structural control in the synthesis of HBPs remains a practical challenge. Methods like step-growth polymerization through polycondensation of AB_*x*_ monomers or A_2_+B_*x*_ monomers,^37−41^ or by self-condensing vinyl polymerization (SCVP) using AB* inimer containing a double bond (A) and initiating group (B*) result in HBPs with limited control over molecular weight, dispersity, and degree of branching.^11,21−23,42−44^ While specific conditions such as gradual addition of more reactive monomer,^45^*in situ* modulations of monomer reactivity through substitution,^37^ microemulsion polymerization,^46,47^ or click polymerization,^48−50^ have proven effective in achieving control, but they substantially restrict the range of monomers and polymerization conditions available.^51^

**Scheme 1:**
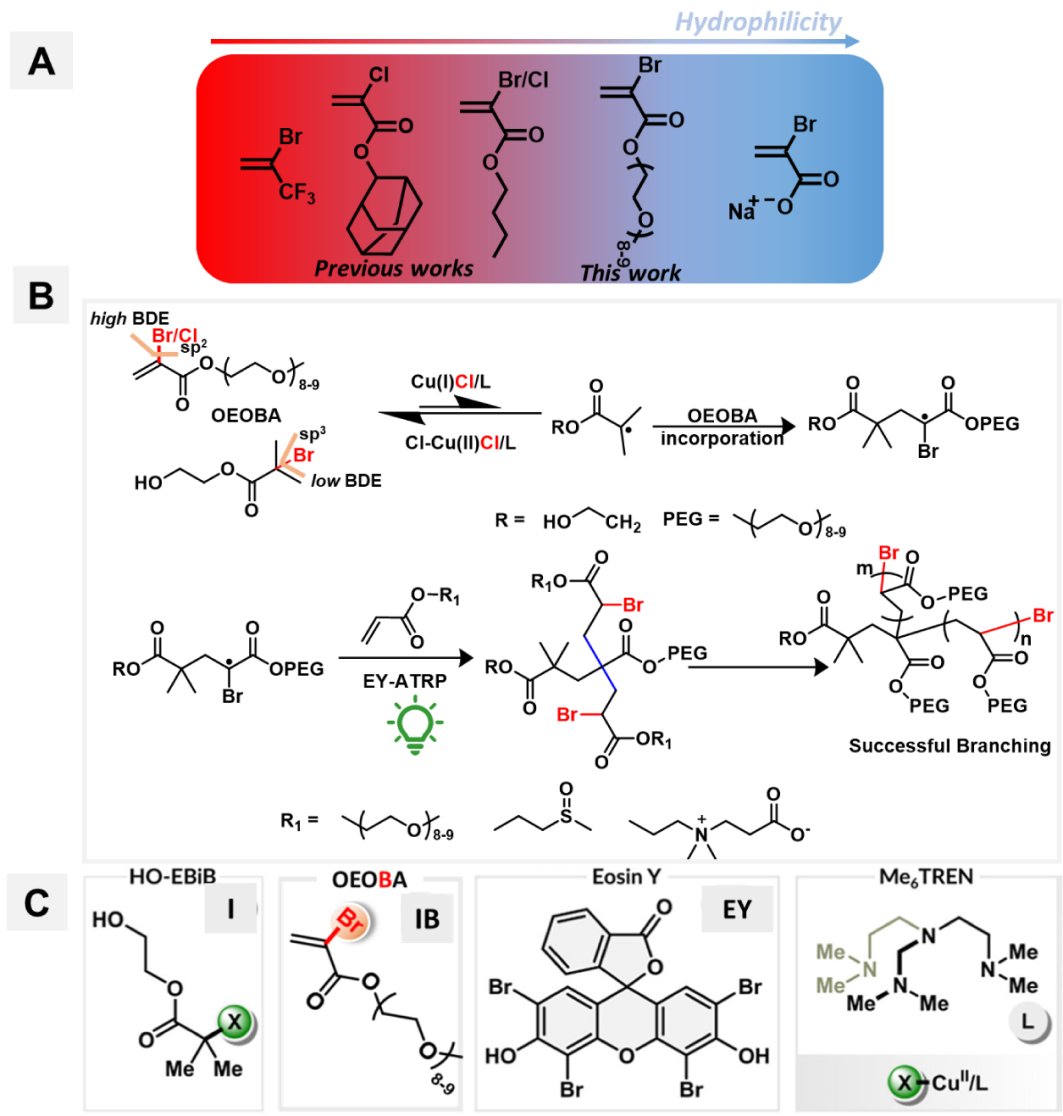
(A) IB Used for CRBP Developed in Previous Work and this Work. (B) Proposed Pathway for Aqueous CRBP to Synthesize HB Polyacrylates Using EY-ATRP. (C) Components of the ATRP Reaction Mixture

Yamago and Zhong have recently introduced a novel and generalized approach for advancing organotellurium-mediated radical polymerization (TERP)^52^ and atom transfer radical polymerization (ATRP)^53^ toward the synthesis of hyperbranched polymers (HBPs). In the conventional AB* inimer system, which exhibits distinct reactivity for A and B* functional groups, it often results in the formation of highly dispersed HBPs. To enhance structural control, novel branching monomers with hierarchical reactivity were engineered. These monomers, coined as “inibramers (IB)″ by Zhong, and “evolmers” by Yamago can trigger the branching process only after their incorporation into the polymer chain. The inibramer/evolmer contains a vinyl halide/telluride bond (C(sp^2^)–X, where X = Cl or Br or TeR) at the α position. This bond has a notably high bond dissociation energy (BDE), rendering it unreactive under typical ATRP or TERP conditions. Nevertheless, once the inibramer is incorporated into the propagating chain, the resulting C(sp^3^)– X bond with a lower BDE can be activated, instigating the initiation of a new branch (Scheme 1B). Zhong demonstrated unprecedented control over the branching of acrylates, styrene, acrylonitrile, and acrylamides, using hydrophobic inibramer such as α-bromo/chloro butyl acrylate in organic solvents.^54^ Chen et al. also reported a one-pot synthesis of well-defined branched fluoropolymers using 2-bromo-trifluoropropene IB (Scheme 1A).^55^ However, both of these methods suffer from the high inibramer reactivity ratio and challenges in regulating copolymerization kinetics.

Our group extended the application of ATRP method in water using a water-soluble ionic inibramer sodium 2-bromoacrylate (SBA) with improved copolymerization kinetic parameters to synthesize hydrophilic hyperbranched polymethacrylates.^56^ TERP under emulsion conditions in water using Brij 98 surfactant for the synthesis of highly branched poly(*n*-butyl acrylate) was reported.^57^

ATRP of acrylates in water is still considered a challenge due to potential hydrolysis of the C(sp^3^)–X (secondary halides are more prone to hydrolysis than tertiary), leading to loss of chain-end functionality since the C(sp^3^)–OH cannot participate in further chain growth.^58−60^ Second, the high values of equilibrium constant of ATRP in water lead to a high concentration of radicals, which may result in more dead chains.^61,62^ These challenges shall be even more pronounced during the controlled synthesis of hyperbranched acrylates using inibramers because their incorporation enhances the concentration of radicals.^63,64^ Recently, we extended the scope EY/Cu photocatalyzed ATRP^65^ resulting in well-defined water-soluble linear acrylates under biorelevant conditions^66−68^ using low-energy green light.^69,70^ The use of Cu^II^/Me_6_TREN as deactivator and EY at ppm levels in 1× phosphate-buffered saline (PBS) medium suppressed the dissociation of the [X–Cu^II^/L]^+^ deactivators and disproportionation of Cu^I^ species to Cu^II^ and Cu^0^ thereby maintaining equilibrium throughout ATRP.

In this study, we aimed to expand the library of water-soluble IBs to advance CRBP in water. A water-soluble PEG-based IB, namely, oligo(ethylene oxide) methyl ether 2-bromoacrylate acrylate (OEOBA) was synthesized. This approach facilitated rapid and well-controlled aqueous branching polymerizations of hydrophilic acrylates under homogeneous conditions in open air under green light. While careful optimization resulted in the successful synthesis of hydrophilic HB polyacrylates, the PEG side chain of the IB was less efficient for the synthesis of HB polymethacrylates. This was attributed to the accelerated fragmentation of the polymethacrylate backbone by midchain radicals (MCR).

## Results and Discussion

### Synthesis of PEG-Based IB

**Figure 1:**
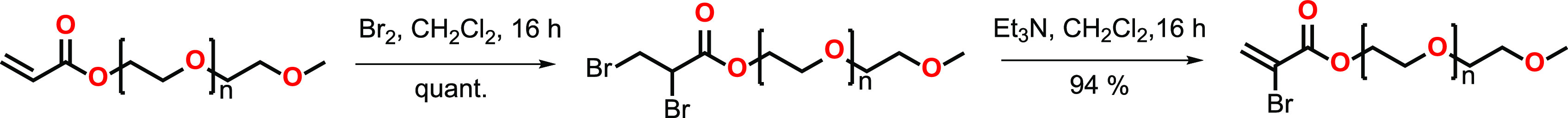
Synthesis of IB oligo(ethylene oxide) methyl ether 2-bromoacrylate (OEOBA).

The new water-soluble PEG-based IB was synthesized in two steps using oligo(ethylene oxide) methyl ether acrylate (*M*_n_ = 480) as the starting material in a one-pot reaction (Figure 1). The addition of bromine (Br_2_) to the double bond resulted in the formation of 2,3-dibromo oligo(ethylene oxide) methyl ether acrylate in quantitative yield. Following the removal of excess Br_2_, this intermediate was directly employed in a subsequent step. Triethylamine allowed for a mild elimination reaction, with selective removal of the β-bromine, to form the final product, oligo(ethylene oxide) acrylate methyl ether 2-bromoacrylate (OEOBA) (see Supporting Information). Progress of the reaction was monitored by ^1^H NMR spectroscopy, while the structures of intermediates and final monomer were conclusively confirmed by ^1^H and ^13^C NMR spectra (Figures S1–S5). The final compound was moderately stable and was stored at 4 °C, which is a common characteristic among IB-type monomers.^53^

### Synthesis of HB Polyacrylates

To synthesize HB-POEOA_480_, we performed copolymerization of oligo(ethylene oxide) methyl ether acrylate (average *M*_n_ = 480, OEOA_480_) with OEOBA using oxygen-tolerant EY/Cu^II^-tris[2-(dimethylamino)ethyl]amine (Me_6_TREN) dual-catalyzed photo redox ATRP. The copolymerization was carried out in the presence of 2-hydroxyethyl α-bromoisobutyrate (HO-EBIB) as the initiator (Table 1) in phosphate-buffered saline (PBS) as the reaction medium to provide biocompatible conditions to suppress dissociation of the [X–Cu^II^/L]^+^ deactivator and to form the highly photoactive form of EY (Scheme 1C). The polymerizations were carried out in open vials placed in a photoreactor with green LEDs (527 nm, 50 mW cm^–2^). The copolymerization of OEOA_480_ (300 mM) and OEOBA (18 mM), conducted in the batch process, resulted in a high conversion of OEOBA inibramer (>99%), but a relatively lower conversion of comonomer, OEOA_480_ (55%) in 60 min (Table 1, entry 1). This indicated that gradient copolymers were synthesized in a batch process due to IB’s higher reactivity than the OEOA_480_ (Figure S6). Similar observations were reported earlier for ATRP in organic media.^54^

**Table 1:** Synthesis of HB-Polyacrylates by Copolymerization of OEOBA and Hydrophilic Acrylate Monomers: OEOA_480_, CBA, MSEA^a^

entry	[M]/[OEOBA]/[I]	feed rate (eq/min)	**α**_**M**_^b^**(%)**	**α**_IB_^b^**(%)**	*M*_n,th_^c^ ([I])	*M*_n,abs_^d^	*Đ*
1	200/12/1	batch	55	>99	55 000	54 500	1.16
2	200/0/1	-	82	-	78 720	84 000	1.09
3	200/4/1	0.06	86	>99	84 800	84 600	1.60
4	200/8/1	0.133	90	>99	90 900	94 200	1.58
5	200/12/1	0.2	85	>99	88 300	92 200	1.55
6	200/24/1	0.3	82	>99	88 800	84 400	1.48
7^e^	200/24/1	0.2	92%	>99	56 900	53 700	1.83
8^f^	200/12/1	0.2	97%	>99	47 000	50 300	1.40
9	100/6/1	0.2	67%	>99	35 500	38 300	1.56
10	400/24/1	0.2	60%	>99	128 600	123 000	1.48
11	600/36/1	0.2	52%	>99	170 100	169 000	1.52

a Reaction conditions: [M]/[OEOBA]/[HO-EBIB]/[EY]/[CuBr_2_]/[Me_6_Tren]: 200/0-24/1/0.01/0.2/0.3, M = OEOA480, MSEA^,d^ CBA^.e^ [OEOBA] = 0–36 mM, [HO-EBIB] = 0.5–1.5 mM in 1× PBS buffer at room temperature, irradiated with green light LEDs (λ_max_ = 527 nm, 50 mW cm^–2^), without deoxygenation for 60 min. Reaction volume 2.0 mL, stirring at 100 rpm.

b aOEOA_480_ and OEOBA conversion was determined by using ^1^H-NMR spectroscopy.

c Theoretical molecular weight was calculated using the equation *M*_n,th_ = [M/I] * MW_M_ *αM + [OEOBA/I]* MW_OEOBA_* αIB +MW_I_.

d *M*_n,abs_ and *Đ* were analyzed using SEC (1× PBS as eluent) equipped with triple detectors: multi-angle light scattering (MALS), refractive index (RI), and inline viscometer and UV detector.

e MSEA was used as a monomer.

f CBA was used as a monomer.

To enable statistical incorporation of OEOBA and thus achieve randomly distributed branching junctions along the polymer backbone, OEOBA was introduced to the polymerization by feeding over 60 min in the subsequent reactions. The molar ratio of OEOBA varied from 2 to 12 mol % to achieve a tunable degree of branching in the synthesized HB-polyacrylates (Table 1, entries 3–6). ^1^H NMR revealed high conversion of both OEOA_480_ and OEOBA. Analysis of the resulting copolymer by size-exclusion chromatography (SEC) with a multiangle light scattering (MALS) detector revealed that the absolute molecular weight (*M*_n,abs_) of copolymers agreed well with their theoretical molecular weights (*M*_n,th_). Furthermore, the HB-POEOA_480_ also possessed slightly higher dispersity values than their linear counterpart (Table 1, entry 2), arising from the broader branching distribution along the polymer backbone (Figure S7).^54,71^ The copolymerization kinetics of OEOBA with OEOA_480_ under these conditions revealed that the *M*_n,abs_ of HB polyacrylates increased as a function of monomer conversion (Figure S8A–C), and agreed well with *M*_n,th_. The molecular weight distribution values also increased as a function of the degree of branching (1.22 ≤ *Đ* ≤ 1.69) during polymerization (Figure S8C).

### Expanding Monomer Scope

The scope of monomers was further expanded to include other water-soluble acrylates. Copolymerization of the OEOBA with 2-(methyl sulfinyl) ethyl acrylate (MSEA) and zwitterionic carboxy betaine acrylate (CBA) afforded well-defined branched polymers with predicted molecular weights (Table 1, entries 7 and 8) and broad molecular weight distributions, that could be attributed to hyperbranched structures (Figure S9).^71^ Furthermore, hydrophobic monomers such as methyl acrylate (MA) and ethyl acrylate (EA) were also copolymerized with the OEOBA inibramer (see Supporting Information). Due to the amphiphilic properties of OEOBA, HB-PMA and HB-PEA were synthesized with good control in DMSO within 90 min under green LEDs (527 nm, 50 mW cm^–2^) without prior deoxygenation (Figure S10). This demonstrated that the catalytic system described herein can also be extended to the nonaqueous polymerization system for hydrophobic monomers.

**Figure 2:**
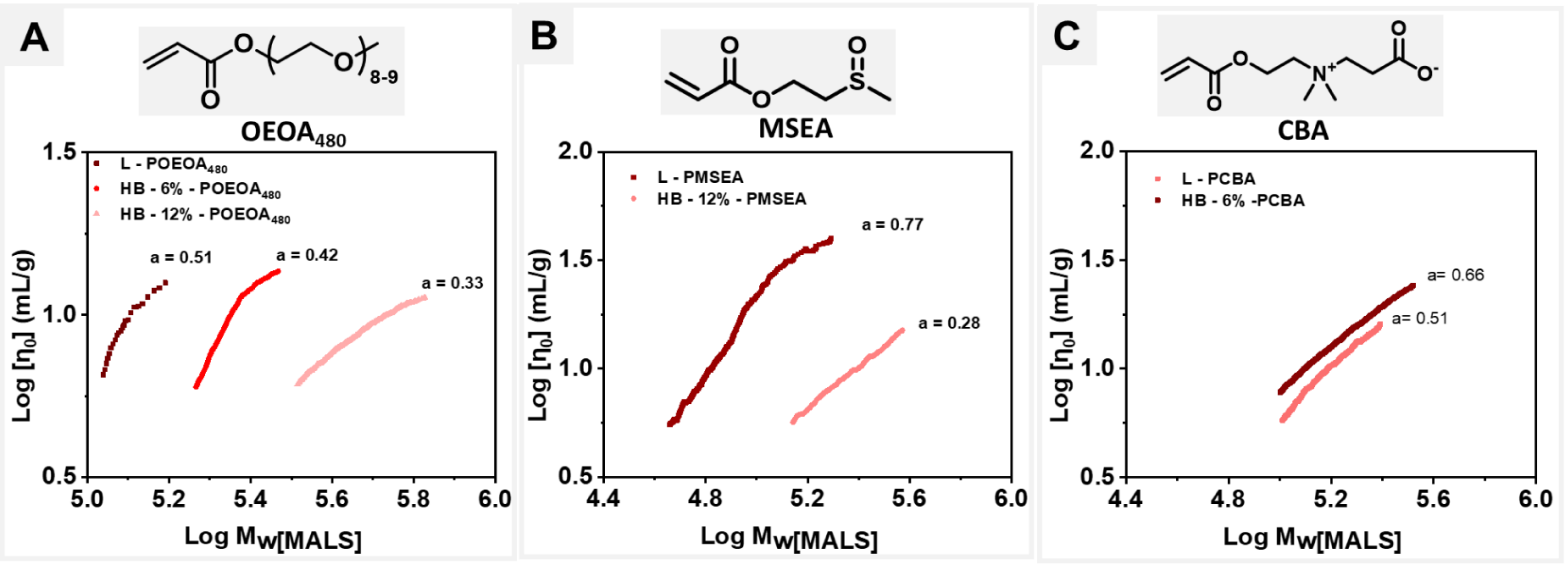
Intrinsic viscosity as a function of molar mass where L refers to linear analogs of (A) HB-POEOA_480_, (B) HB-PMSEA, and (C) HB-PCBA. The slope values, known as Mark–Houwink parameter “a”, correspond to the polymer conformation.

To characterize the degree of branching, the molar mass and the molecular size were determined simultaneously and independently using SEC-MALS equipped with a triple detector (UV, RI, Viscometer) and inline DLS. A Mark–Houwink–Sakurada (MHS) plot showing intrinsic viscosity as a function of molar mass (log–log plot of [η] versus M), revealing the polymer conformation, was obtained (Figure 2A). The slope of HB-POEOA_480_ had lower values as compared to linear POEOA_480_ as indicated by the MH constant “a”. Also, subsequent reduction in “a” values was observed for a higher molar ratio of inibramer, confirming the successful synthesis of well-defined hyperbranched polyacrylates with tunable branching ratio.^35,72^ Similar MHS plots for HB-PMSEA (Figure 2B) and HB-PCBA (Figure 2C) also revealed a lowering in slope values (“a”) as a function of the degree of branching.

### Varying Targeted Degrees of Polymerization (DP)

**Figure 3:**
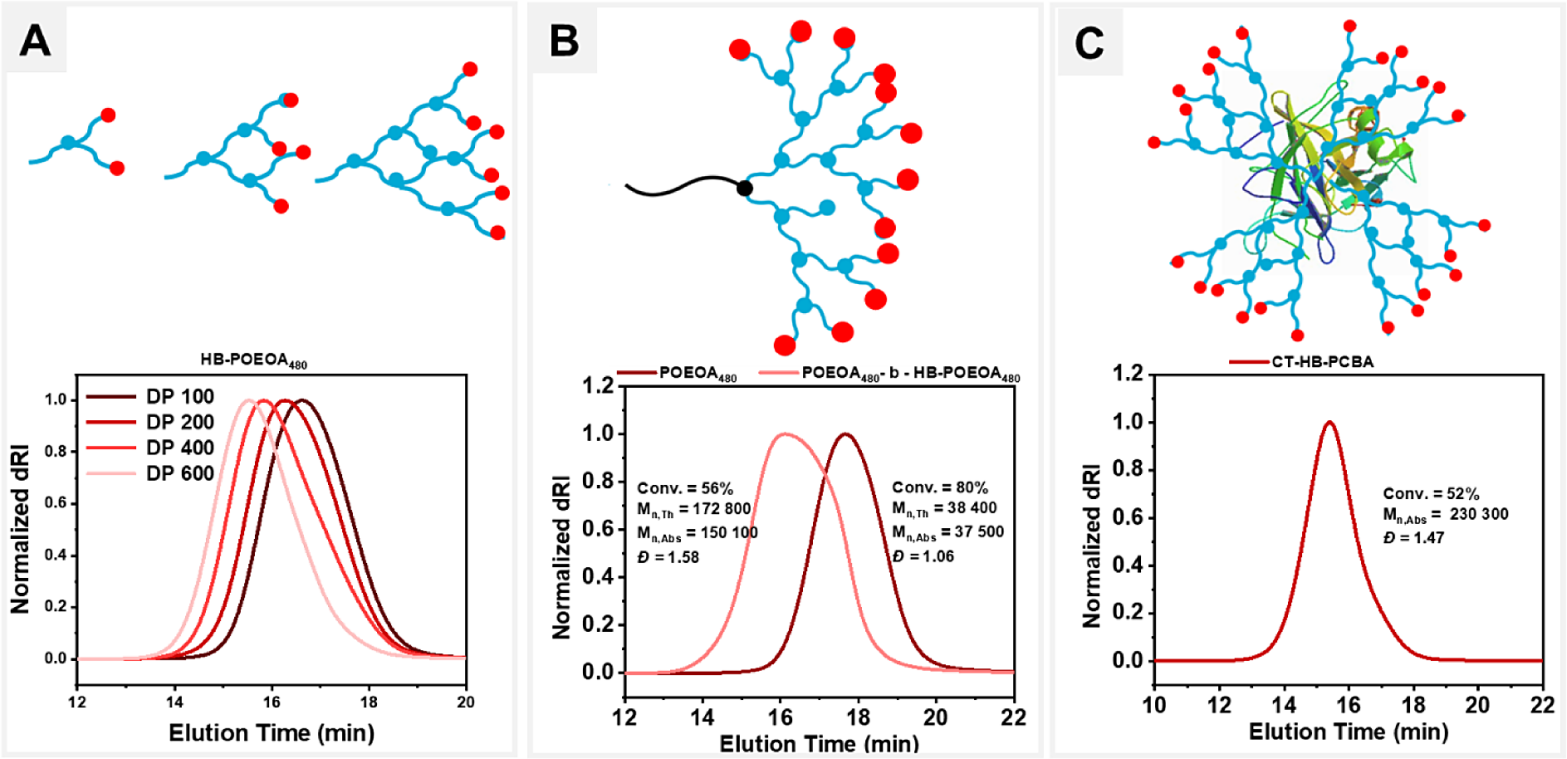
Synthetic scope of HB polyacrylates; (A) varying targeted degree of polymerization (DP), (B) *in situ* chain extension to form linear-HB block copolymer. (C) Synthesis of HB-PPH using CT to form CT-HB-PCBA.

The degree of polymerization was varied (DP = 100–600) to obtain HB polymers with variable molecular weights and degrees of branching. The polymerization was carried out by varying the HO-EBIB concentration while keeping the concentrations of the other reagents constant and slowly feeding the OEOBA for 60 min (Table 1, entries 9–11). HB-POEOA_480_ exhibited predictable *M*_n,abs_ and broader molecular weight distribution (*Đ* ≤ 1.56), typical for a branched polymer (Figure 3A).^71^ Lack of deviations from *M*_n,th_ suggests the absence of new polymer chains or backbone fragmentation, contrary to observations below with polymethacrylate backbones.^56^

**Table 2:** Copolymerization of OEOMA_500_ with OEOBA^a^

entry	[M]/[IB]/[I]	**α**_M_^b^**(%)**	**α**_IB_^b^**(%)**	***M***_n,th_^c^**([I])**	*M*_n,app_^d^	*M*_n,abs_^e^	*Đ*
1.	200/4/1	93	>99	87 600	23 400	32 000	1.20
2.	200/12/1	83	>99	80 800	11 000	16 000	1.17
3.	200/0/1	88	-	88 200	60 000	90 000	1.06

a Reactions conditions: [OEOMA_500_]/[OEOBA]/[HO-EBIB]/[EY]/[CuBr_2_]/[TPMA]: 200/12/1/0.01/0.2/0.6, [OEOMA_500_] = 300 mM, [OEOBA]= 6–18 mM, [HOBIB] = 1.5 mM in 1× PBS buffer at room temperature, irradiated for 30 min under green light LEDs (λ_max_ = 520 nm, 9.0 mW cm^–2^) without deoxygenation. Reaction volume 4.4 mL.

b aOEOMA_500_ and OEOBA conversions were determined by using ^1^H NMR spectroscopy.

c *M*_n,th_ was calculated using the equation *M*_n,th_ =[M/I] * MW_OEOMA50_0 *α_M_ + [OEOBA/I]* MW_OEOBA_* α_OEOBA_ +MW_HO-EBIB_.

d *M*_n,app_ analyzed using GPC (DMF as an eluent), calibrated with PMMA standards.

e *M*_n,abs_ analyzed using SEC (1× PBS as eluent) equipped with triple detectors: MALS, RI and inline viscometer and UV detector.

### Linear-HB Topological Block Copolymer Synthesis

Next, an *in situ* chain extension experiment was performed to analyze the chain-end fidelity. The linear POEOA_480_ macroinitiator (conv. = 80%, *M*_n,abs_ = 37 500, *Đ* = 1.06) was synthesized (see Supporting Information). A sample was taken from the postpolymerization mixture and used without further purification to prepare a new reaction mixture containing the OEOA_480_ (300 mM). After 60 min of slowly feeding OEOBA (18 mM) and of green light irradiation (λ_max_ = 527 nm, 50 mW cm^–2^), in an ambient temperature, OEOA_480_ conversion was 56%. The SEC-MALS analysis showed a clear shift toward higher molecular weights without any shoulder or tailing at lower molecular weights (*M*_n,abs_ = 150 100, *Đ* = 1.58), indicating a controlled polymerization and high retention of chain end functionality (Figure 3). The absence of a low molecular weight shoulder in SEC traces indicated negligible fragmentation and successful chain extension from the macroinitiator, resulting in linear-HB topological block copolymer.

### Synthesis of HB-Protein Polymer Hybrids (PPH)

Owing to the benign condition of EY/Cu catalyzed aqueous CRBP, the grafting-from approach was applied to graft HB-PCBA from the surface of chymotrypsin (CT) enzyme, functionalized with 12 ATRP initiators, to achieve tunable molecular sieving.^73^ HB-PCBA was grafted from the CT macroinitiator.^74^ The copolymerization of CBA (300 mM) and OEOBA (18 mM) fed at the rate of 0.2 eq/min was carried out in a Lumidox photoreactor (527 nm, 125 mW/cm^2^) at 15–18 °C to preserve the activity of CT. The purified bioconjugate was analyzed by^1^H NMR (conv. = 52%) and SEC-MALS (*M*_n,abs_ = 230 300, *Đ* = 1.47) (Figure 3). High molecular weight and monomodal molecular weight distribution indicated a well-controlled branching polymerization.

### Copolymerization of OEOBA with OEOMA_500_

Encouraged by the successful CRBP of OEOA_480_, we employed an OEOBA to synthesize HB hydrophilic polymethacrylates. The copolymerization of OEOBA and a methacrylate comonomer, oligo(ethylene oxide) methyl ether methacrylate (average *M*_n_ = 500, OEOMA_500_), was performed using molar ratios of [OEOMA_500_]/[OEOBA]/[HO-EBIB]/[EY]/[CuBr_2_]/[TPMA] = 200/4/1/0.01/0.2/0.6. High conversions of OEOMA_500_ and OEOBA were observed within 30 min (Table 2, entry 1). The apparent molecular weight of the resulting copolymer (*M*_n,app_ = 23 400) determined by SEC calibrated with linear poly(methyl methacrylate) standards was significantly lower than the *M*_n,th_ value (Figure S11A). The lower *M*_n,app_ values than *M*_n,th_ values are typical for HBPs due to the lower hydrodynamic volume. Nevertheless, analysis of the resulting copolymer by SEC with a MALS detector revealed that the *M*_n,abs_ of the copolymer was also much lower than that of the *M*_n,th_ (Figure S11B). In addition, the resultant copolymer had a narrow molecular weight distribution (*Đ* = 1.20) when compared to that of HB-POEOA_480_ of the same OEOBA and OEOA_480_ feed ratio (*Đ* = 1.60). Copolymerization with a higher molar ratio (6 mol %) of OEOBA (Table 2, entry 2) resulted in an even lower value of measured M_n,app_, and *M*_n,abs_ values, despite the high conversion of both OEOBA and OEOMA_500_, as determined by ^1^H NMR. The homopolymerization of OEOMA_500_ resulted in a well-controlled linear polymer consistent with previous observations (Table 2, entry 3).^65^ The low molecular weight polymers indicated unsuccessful branching in the case of methacrylate, which was further verified by following the copolymerization kinetics of OEOMA_500_ and OEOBA and the synthesis of model polymers with halogens in the backbone.

**Figure 4:**
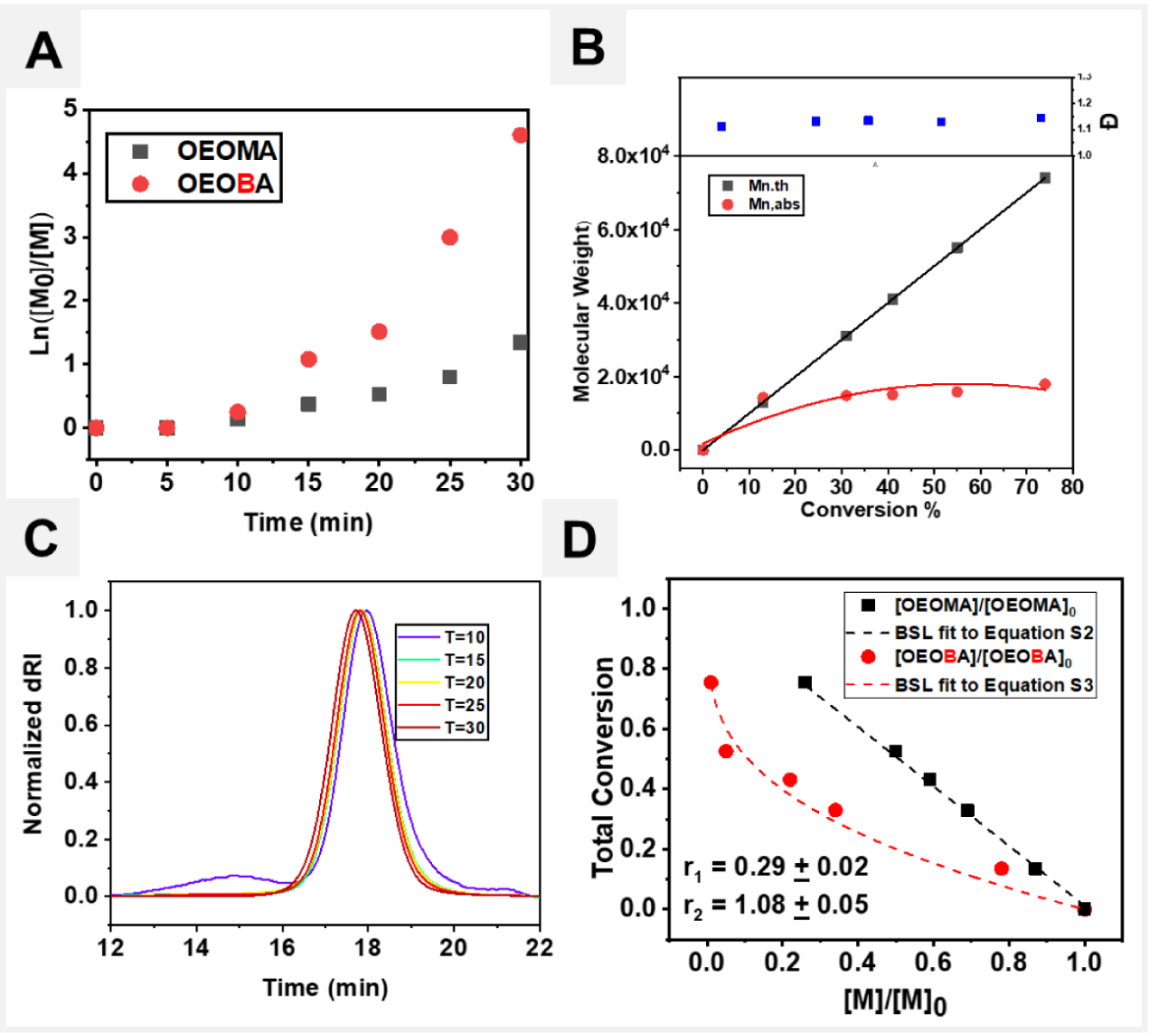
Copolymerization kinetics using molar ratios [OEOMA_500_]/[OEOBA]/[HO-EBIB]/[EY]/[CuBr_2_]/[TPMA] = 200/12/1/0.01/0.2/0.6. (A) First-order kinetic plot. (B) Evolution of molecular weight and molecular weight distribution with % conversion-depict poor control. (C) SEC traces evolution with time depict no shifts. (D) BSL curve fitting to kinetics experiments depicts 4× higher reactivity ratio.

The copolymerization kinetics of OEOMA_500_ and OEOBA was monitored by performing the polymerization reaction under same conditions as above (Table 1, entry 2) using molar ratios as follows: [OEOMA_500_]/[OEOBA]/[HO-EBIB]/[EY]/[CuBr_2_]/[TPMA] = 200/12/1/0.01/0.2/0.6). The samples were drawn out at regular intervals (5 min), quenched with 1,4-bis(3-isocyanopropyl) piperazine,^75^ and then analyzed by ^1^H NMR and SEC-MALS. The copolymerization exhibited first-order kinetics with a short induction period of 5 min, followed by a rapid polymerization, reaching 78% and >99% of OEOMA_500_ and OEOBA conversion within 30 min, respectively (Figure 4A). The rate of the conversion of OEOBA was higher than that of the conversion of OEOMA_500_, suggesting the gradient incorporation of the conversion of OEOBA. The polymer samples showed no increase in the observed *M*_n,abs_ values with an increase in the monomer conversion (Figure 4B). In addition, SEC revealed overlapping traces and did not show any shift in the elution time with increasing monomer conversion. However, they remained narrow throughout polymerization (Figure 4C). The Beckingham–Sanoja–Lynd (BSL)^76^ copolymerization model used to estimate the reactivity ratio, revealed 4 times higher reactivity of OEOBA inibramer as compared to OEOMA_500_ (*R*_1,OEOMA_ = 0.29, *R*_2,OEOBA_ = 1.08 Figure 4D). This is in agreement with previously observed higher reactivity ratio of IB relative to butyl acrylate,^53^ but unlike comparable reactivity ratio between OEOMA_500_ and SBA.^56^

### Analysis of POEOMA_500_ Fragmentation Through Br-Activated MCR

**Scheme 2:**
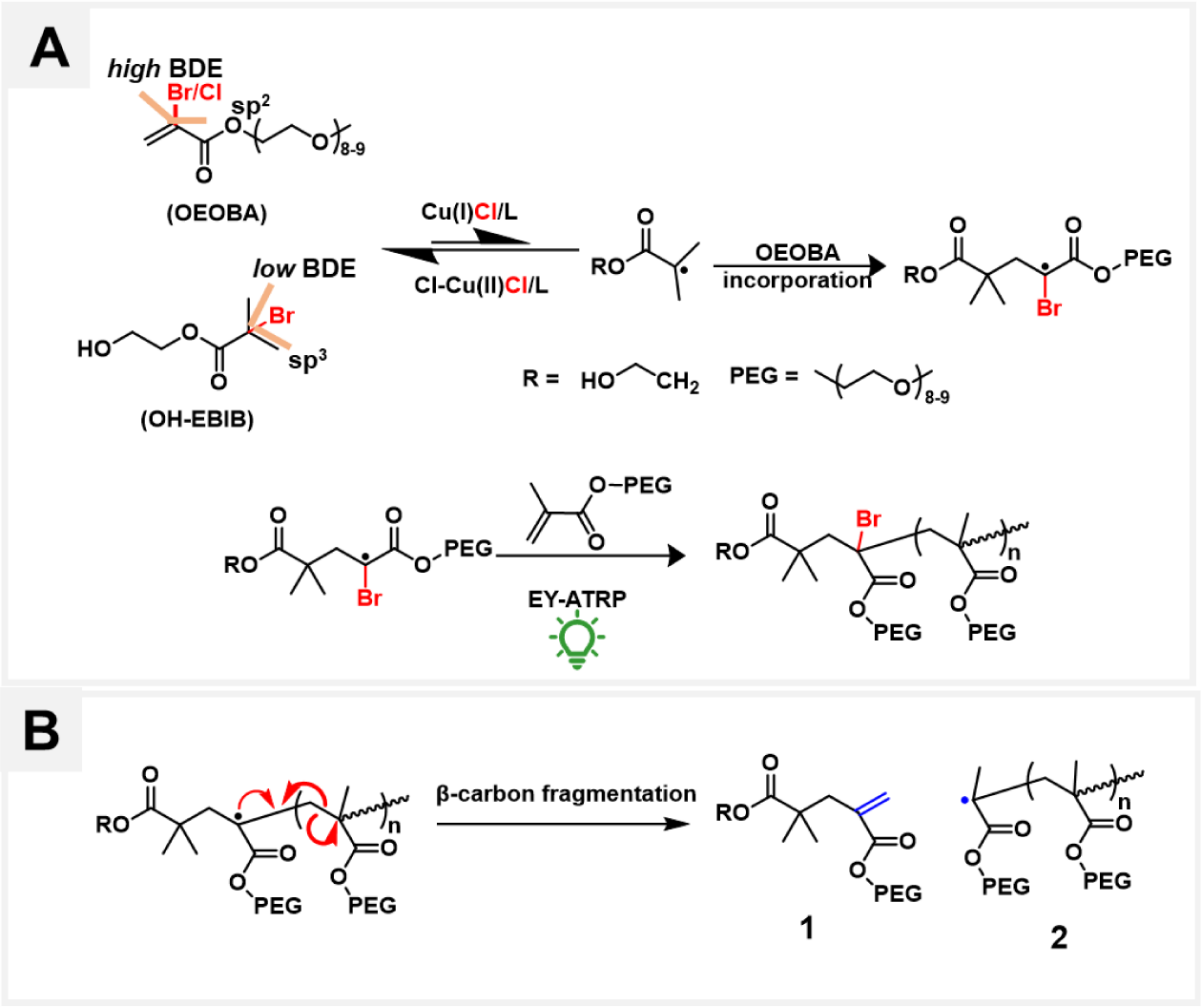
Proposed Mechanism for the (A) Copolymerization of OEOMA_500_ and OEOBA. (B) β-Carbon Fragmentation of Radical Intermediates

The lower-than-expected molecular weights can be attributed to the β-carbon fragmentation of the POEOMA_500_ backbone via formation of midchain radicals (MCRs) upon activation of C–Br bond (Scheme 2).^77,78^ The MCRs generated from C–Br bonds in the backbone of polyacrylates were sufficiently stable to synthesize graft/brush copolymers,^79^ whereas the polymethacrylates underwent fragmentation using ruthenium or photocatalysts.^80−82^

**Figure 5:**
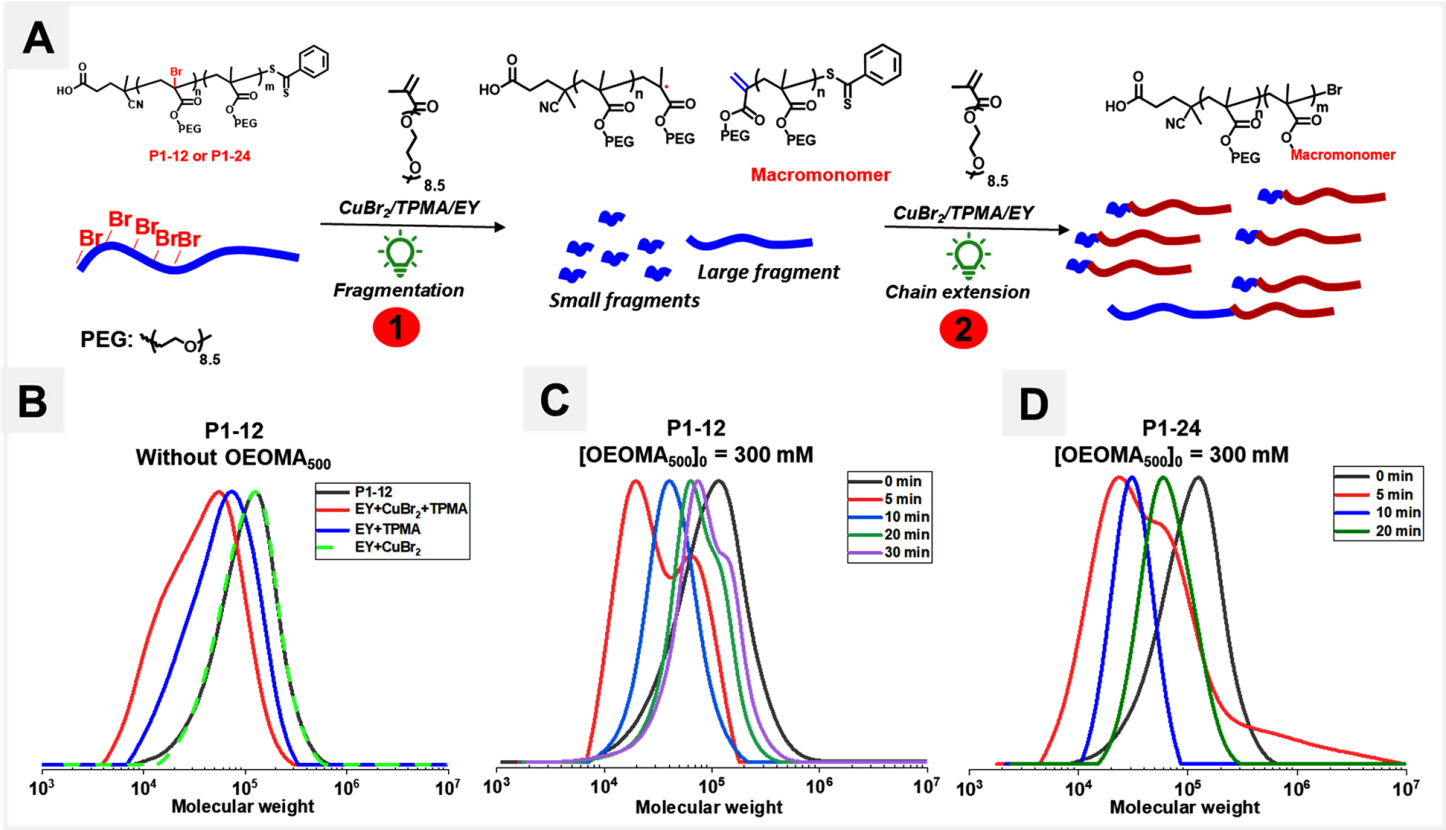
(A) Two steps fragmentation of Br-activated MCR in POEOMA_500_-co-POEOBA copolymers and chain extension from fragmented chains during EY-ATRP. (B) SEC traces of P1–12 in the absence of OEOMA_500_. (C) SEC traces of P1–12 in the presence of OEOMA_500_ (D) SEC traces of P1–24 in the presence of OEOMA_500_.

To investigate this under our polymerization conditions, two model copolymers of OEOMA_500_ and OEOBA were synthesized using sodium pyruvate (SP)-reversible addition–fragmentation (RAFT) polymerization with different feed ratios of OEOBA (target DP of OEOBA = 12 and 24), yielding POEOMA_500_ copolymers (P1–12 and P1–24) with midchain Br atoms (Figure 5). The successful one-pot synthesis of copolymers was confirmed (^1^H NMR and SEC in Figures S13 and S14) and no activation of C–Br was observed during RAFT polymerization.^83,84^ The higher reactivity of OEOBA versus that of OEOMA_500_ affords gradient copolymers with rich C–Br sequences at the beginning of chains.^85^

The copolymers were then exposed to EY-ATRP conditions to generate radicals by activating C–Br (Figure 5A). Upon exposing P1–12 to green light irradiation in the presence of CuBr_2_, TPMA, and EY (without monomer), the SEC traces shifted to a lower molecular weight region (*M*_n,app_ before irradiation = 92 000, *Đ* = 1.42; *M*_n,app_ after irradiation = 26700, *Đ* = 1.91), suggesting the homolytic cleavage of C–C bonds through the MCR formation (Figure 5B). Degradation of P1–12 also occurred in the absence of metal (CuBr_2_), and only in the presence of EY and TPMA (*M*_n,app_ after irradiation = 49 000, *Đ* = 1.66). However, the SEC traces remained nearly unchanged in the presence of EY and CuBr_2_ (without an electron donor, TPMA) or with EY alone, even after prolonged irradiation (16 h) in deoxygenated media (Figure S15). This suggests that EY-catalyzed C–Br activation of midchain halogens primarily occurs through a reductive quenching mechanism involving electron transfer (ET) from TPMA amines to EY, followed by C–Br activation through electron transfer from EY. The degradation of P1–12 did not yield low molecular weight fragments corresponding to 12 times lower molecular weight species. This can be attributed to the higher reactivity ratio of OEOBA versus OEOMA_500_ (as mentioned earlier), resulting in the gradient incorporation of OEOBA during one-pot RAFT copolymerization.

We hypothesized that the presence of monomers (OEOMA_500_) might suppress fragmentation by reacting with radicals formed in the backbone, thereby promoting the generation of brush copolymers over fragmentation (Figure S12). To investigate this, a midchain C–Br activation experiment for P1–12 and P1–24 was conducted in the presence of all polymerization components (CuBr_2_+TPMA+EY) along with OEOMA_500_ (300 mM), and the process was monitored over time. Under this condition, after 5 min of irradiation, both P1–12 and P1–24 exhibited degradation, as evidenced by the formation of lower molecular weight species in SEC (Figure 5C,D) and ^1^H NMR (Figure S16), followed by a gradual increase in SEC traces toward the higher molecular weight region, as the polymerization progressed. This unequivocally indicated that fragmented chains acted as initiators to form new chains (Figure 5A), resulting in polymers with a final *M*_n,abs_ lower than *M*_n,th_. Notably, β-carbon fragmentation yielded two polymer species: one with a radical and another with an unsaturated end chain (macromonomer), which can either initiate or be incorporated into new polymer chains. Attempts to suppress fragmentation by using higher monomer concentrations ([OEOMA_500_]_0_ = 800 mM) led to gelation. This revealed that MCR-induced fragmentation is a predominant event in polymethacrylates and cannot be prevented under these experimental conditions.

To suppress the rate of fragmentation, thereby increasing the structural control in HB polymethacrylates synthesized by TERP, low-temperature conditions were reported to be more suitable.^77^ To achieve successful branching, EY/Cu-mediated ATRP was then performed under low-temperature conditions with slow feeding of the OEOBA inibramer. The copolymerization kinetics was slower as the polymerizations were carried out at 12 °C and further lower at 5 °C as revealed by the conversion of the monomer measured by ^1^H NMR (Table S2). However, SEC-MALS analysis of resultant copolymers showed only a very small improvement in the observed *M*_n,abs_ as compared to copolymerization conducted at 25 °C. There still was poor agreement between *M*_n,th_ and *M*_n,abs_. The SEC traces did not shift to higher molecular weight regions with an increase in conversion of the OEOMA_500_ (Figure S17). This indicated that the rate of propagation and, hence, successful branching event competed with the rate of fragmentation because of the instability of the polymer backbone due to the incorporated inibramer.

### Comparison between OEOBA and SBA During Copolymerization with OEOMA_500_

**Figure 6:**
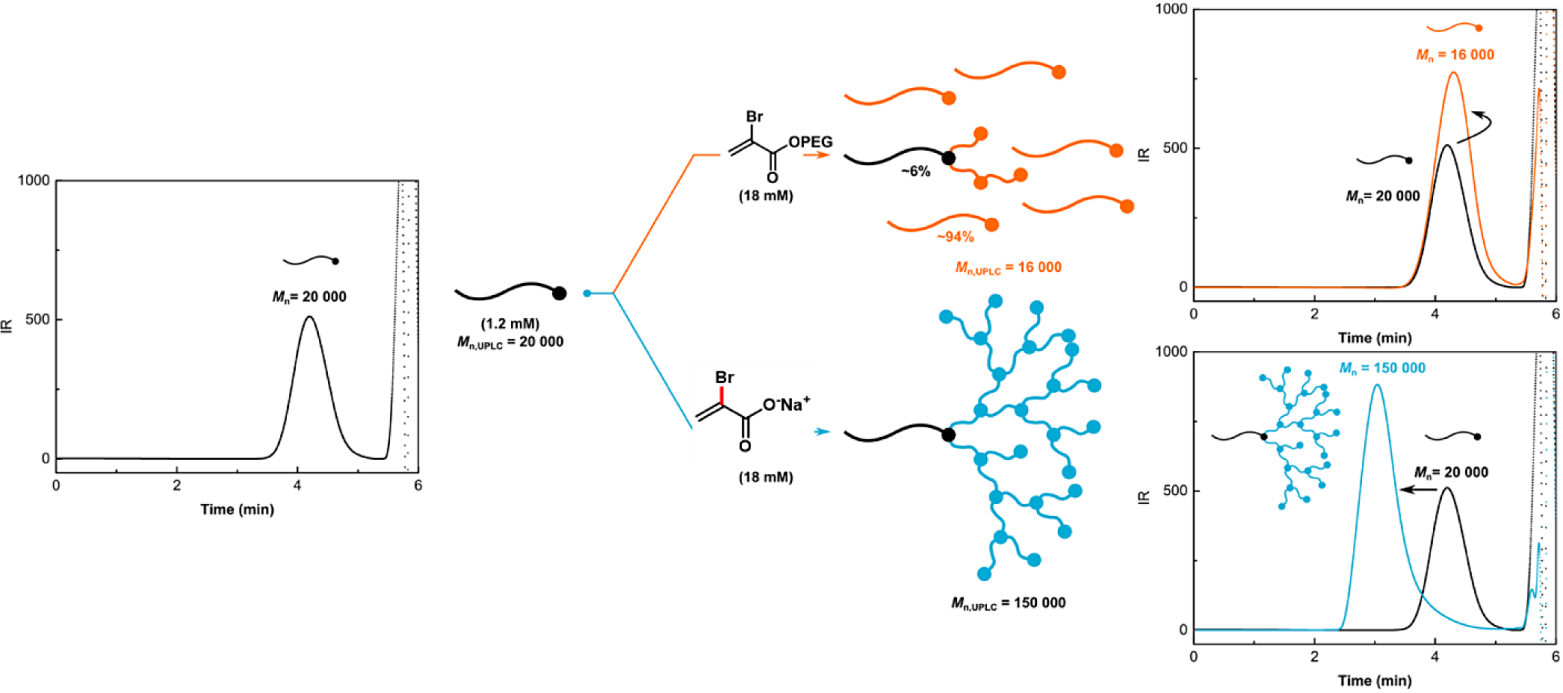
Comparison between OEOBA and SBA in an *in situ* chain extension experiment toward synthesis of POEOMA_500_-*b*-HB-POEOMA_500_.

To compare the performance of OEOBA and the previously reported SBA inibramer^56^ during copolymerization with OEOMA_500_, an *in situ* chain extension experiment was conducted using a linear POEOMA_500_ (*M*_n,abs_ = 20 000) as a macroinitiator which was further extended with a second block using either OEOBA or SBA with OEOMA_500_. Ultraperformance liquid chromatography (UPLC) analysis of resultant copolymers indicated no shift in the trace of macroinitiator when the OEOBA was used. Conversely, SBA enabled grafting of HB-POEOMA_500_ block as revealed by a clear shift of polymer trace (Figure 6). This highlights the critical role of the IB structure in the CRBP. The SBA, with a short ionic side chain, promotes rapid propagation of radicals instead of fragmentation of the polymethacrylate backbone, whereas the OEOBA, with the relatively longer side chain of PEG, resulted in fragmentation.

### Computational Analysis of Total Energies for Model Substrates and Fragments

**Scheme 3:**
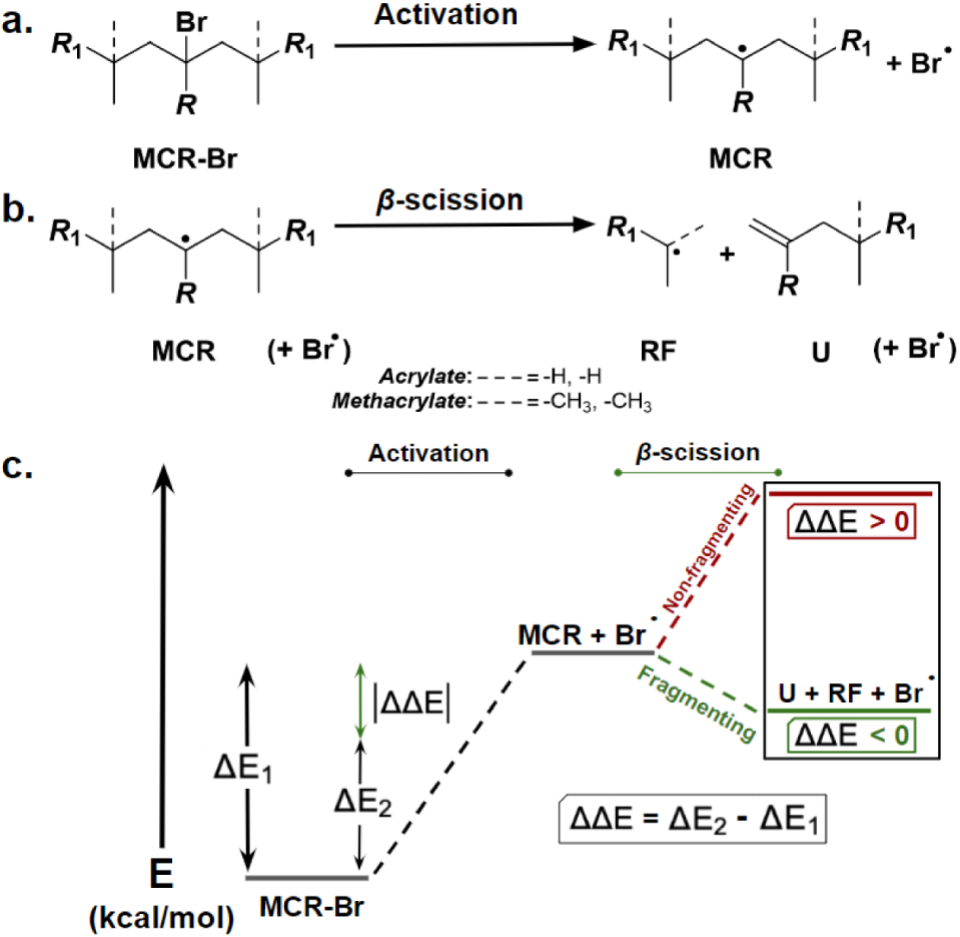
Model Substrates Bearing a Central Inibramer Unit Flanked by either Acrylate- or Methacrylate-based Moieties The activation reaction of a model midchain alkyl halide (MCR-Br) to form a Br atom and MCR. (b) The β-scission reaction of an MCR forming corresponding radical fragment (RF) and unsaturated (U) species (Br atom is considered stoichiometrically for total energies but is not a reactant). (c) Energy reaction coordinate diagram for the substrates and products of the above reactions, with favorable (green) and unfavorable (red) fragmentation pathways based on calculated ΔΔ*E* values. Δ*E*_1_ is the difference in total energies between activated intermediate product and starting alkyl halide substrate, Δ*E*_2_ is the difference in total energy between β-scission products and starting alkyl halide substrate, and ΔΔ*E* is the difference between Δ*E*_2_ and Δ*E*_1_, shown (green arrow) as an absolute value.

To gain insight into the fragmentation tendency, computational studies were carried out on model inibramer substrates with surrounding acrylates and methacrylates. Reactants and products were designated within the activation and β-scission reactions (Scheme 3a,b). Through the course of the overall reaction sequence, monomer–inibramer–-monomer (i.e., symmetric trimeric species of ABA, where A = comonomer and B = IB) model alkyl halides substrate (MCR-Br) was activated to form MCR and atomic Br, whereby MCR fragmented into radical (RF) and unsaturated (U) fragments. The energy coordinate diagram for the activation-scission pathway for model compounds is shown in Scheme 3c. Total energies were compiled, and the reported relative energies follow the following equations:
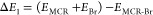

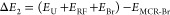

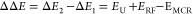


Using ΔΔ*E*, the relative tendency of midchain radical (MCR) to undergo β-scission into more stable fragments was investigated, where negative values indicate fragmentation products as more stable than their corresponding MCRs, whereas positive ΔΔ*E* values indicate the opposite.

Total energies of substrates, intermediates, and final products along the activation-scission pathway were compared for their tendency to fragment. For computational efficiency and simplicity, the following assumptions were used:

1. Trends of fragmentation were explored broadly but reliably using trimeric substrates as opposed to extended penta-, hepta-, or *n*-meric (i.e., higher oligomeric or polymeric) structures.

2. Ester functionalities with oligo(ethylene oxide) substituent derived from OEOA_480_, OEOMA_500_, and OEOBA were simplified to the corresponding β-methoxyethyl ester models (−CO_2_CH_2_CH_2_OMe), while comonomers and IB bearing −CO_2_Me ester moieties served as control model compounds.

3. For acrylate models, synthesized copolymers realistically would contain all possible stereochemical configurations neighboring their corresponding inibramer units, but their relative tendencies for fragmentation should all be similar (Figure S17, ESI).

Given these assumptions, the trends of fragmentation should be exhibited even within the model system by comparing acrylate- or methacrylate-based units flanking the OEOBA-derived inibramer. Indeed, similar symmetric “ABA” model substrates were analyzed by Yamago and coworkers for fragmentation selectivity in which a MCR moiety was flanked by two comonomer units, although with the branching point bearing a hydrogen or methyl and not ester, and not from the context of starting from any corresponding alkyl halide form.^77^

**Figure 7:**
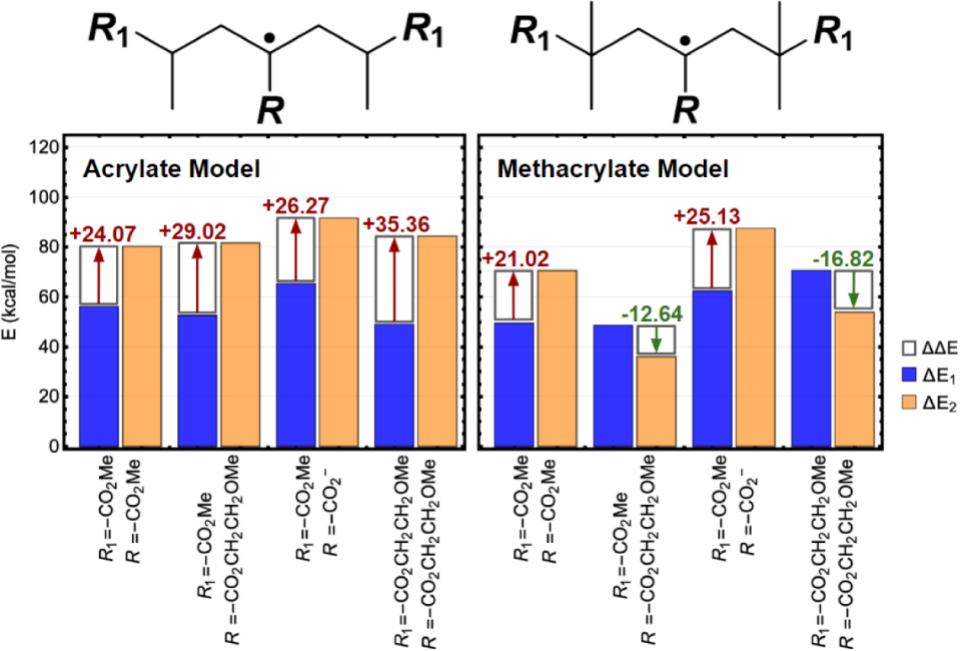
Corresponding Δ*E*_1_, Δ*E*_2_, and ΔΔ*E* energies for substrates bearing different substituents (*R* derived from inibramer and *R*_1_ from comonomer) in acrylate (left) and methacrylate (right) models. Values were obtained at the ωB97X-D4 def2-TZVP level of theory.

Fragmentation tendency inferred from total energies provided by calculations agreed with experimental fragmentation data based on the IB systems. Regardless of the inibramer-derived substituent within the acrylate model, all Δ*E*_2_ values were considerably higher than their corresponding Δ*E*_1_ values by ∼24–35 kcal/mol, indicating unfavorable β-scission products (positive ΔΔ*E* values) relative to their MCR analogs. Within the methacrylate model, positive ΔΔ*E* values were found when the inibramer moiety was based on small substituents: methyl- and sodium 2-bromoacrylate (i.e., *R* = −CO_2_Me and −CO_2_–, respectively, flanked with units bearing *R*_1_ = CO_2_Me). This indicates that copolymers of sodium 2-bromoacrylate with methyl acrylate or methacrylate would not appreciably fragment (Figure 7).

However, the inibramer model bearing an β-methoxyethyl variant (*R* = −CO_2_CH_2_CH_2_OMe, mimicking OEOBA) with both methacrylate comonomers (*R*_1_ = −CO_2_Me or −CO_2_CH_2_CH_2_OMe) exhibited favorable fragmenting with Δ*E*_2_ values lower than the corresponding Δ*E*_1_ and thus the only negative ΔΔ*E* values. Their negative ΔΔ*E* values indicate favored β-scission products relative to their corresponding MCRs. This supports the observed tendency of POEOMA_500_-*co*-POEOBA to undergo fragmentation upon halide activation during chain extension or branching conditions, where midchain halides could activate and fragment to generate lower molecular weight polymers. On the other hand, POEOA_480_-*co*-POEOBA exhibited nonfragmenting MCRs and instead persisted to undergo addition to comonomer and create branching points, agreeing with the relatively high ΔΔ*E* values found in the acrylate model.

This limited study showed that methacrylate units flanking the OEOBA play a key role in favoring fragmentation products. Flanking acrylate units lead to unfavorable fragmentation products, regardless of the nature of the inibramer substituent. Furthermore, only methacrylate-based model compounds with inibramer units derived from the OEOBA (i.e., *R* = −CO2CH2CH2OMe) showed favorable β-scission products relative to their corresponding MCRs, as demonstrated by negative ΔΔ*E* values. Future studies could include longer PEO substituents, more (co)monomer units appended to the substrate, and the addition of a solvent phase to the calculations. The use of model macromonomer, considerations regarding activation, the potential role of Cu, and the effect of stereochemistry are discussed in the electronic support information (ESI).

## Conclusions

In conclusion, we have demonstrated successful synthesis and application of the novel oligo(ethylene oxide) methyl ether 2-bromoacrylate (OEOBA) IB toward the preparation of structurally controlled HB polyacrylates with the predetermined degree of branching, molecular weight, and architecture. Furthermore, the remarkable oxygen tolerance of green-light-induced dual EY/Cu-catalyzed ATRP, coupled with the fast kinetics and the retention of chain-end functionality, attests to the versatility of this technique in synthesis of topological block copolymers and HB-PPH. However, the incorporation of OEOBA in a polymethacrylate backbone resulted in backbone degradation under benign conditions. The computation studies revealed that flanking the inibramer with methacrylate units favored fragmentation products but only when employing an OEOBA-like IB. These findings emphasize the importance of IB and comonomer structures and highlight the potential for advancing CRBP methodologies, particularly in water. This research also contributes to expanding the toolkit of water-soluble IB and opens avenues for the tailored synthesis of intricate macromolecular structures with enhanced precision and efficiency.

## Supporting Information Available

Experimental details including methods, instrumentation, and procedures, characterization results by SEC-MALS, details of computation methods and additional references (PDF)
