# Supplementary material for: Hydrophilic Poly(meth)acrylates by Controlled Radical Branching Polymerization: Hyperbranching and Fragmentation
Source: Macromolecules. 2024 May 29;57(11):5368–79. doi: 10.1021/acs.macromol.4c00408 (PMC11171460; doi:10.1021/acs.macromol.4c00408)
Supplement: Supplementary file 1 — ma4c00408_si_001.pdf [file ma4c00408_si_001.pdf]

Supporting Information

Hydrophilic Poly(meth)acrylates by Controlled Radical Branching Polymerization: Hyperbranching and Fragmentation

Kriti Kapil,[1] Arman Moini Jazani,[1] Julian Sobieski,[1] Leticia P. Madureira,[1] Grzegorz Szczepaniak,[1,2] Michael R. Martinez,[1,3] Adam Gorczyński,[1,4] Hironobu Murata,[1] Tomasz Kowalewski[1] and Krzysztof Matyjaszewski[1]*

[1] Department of Chemistry, Carnegie Mellon University, 4400 Fifth Avenue, Pittsburgh, PA 15213, United States.

[2] Faculty of Chemistry, University of Warsaw, Pasteura 1, 02-093 Warsaw, Poland.

[3] PPG Industries, Inc. 4325 Rosanna Drive, Allison Park, Pennsylvania, 15101, United States.

[4] Faculty of Chemistry, Adam Mickiewicz University, Uniwersytetu Poznańskiego 8, 61-614 Poznań, Poland.

Table of Contents

Experimental Details 3

Materials 3

Instrumentation 3

Nuclear Magnetic Resonance (NMR) 3

Size Exclusion Chromatography (RI Detector- DMF or THF as an eluent) 3

Size Exclusion Chromatography with Multi-Angle Light Scattering (SEC-MALS- 1X DPBS eluent) 4

Procedures 4

Synthesis of poly(ethylene glycol) PEG-based inibramer oligo(ethylene oxide) methyl ether 2-bromo acrylate (OEOBA) 4

Synthesis of HB-POEOA$_{480}$ by copolymerization of inibramer OEOBA and OEOA$_{480}$ by EY/Cu-catalyzed ATRP 9

Batch copolymerization. 9

Semi-batch copolymerization 10

Varying targeted degrees of polymerization (DP) 14

Synthesis of topological block copolymer (L-POEOA$_{480}$ –b–HB–POEOA$_{480}$) 14

Synthesis of hyperbranched protein polymer-hybrid (HB-PPH) 15

Copolymerization of OEOBA and OEOMA$_{500}$ by EY/Cu-catalyzed ATRP 15

Analysis of POEOMA$_{500}$ fragmentation induced by Br-activated midchain radicals (MCR) 18

Comparison between OEOBA and SBA during copolymerization with OEOMA$_{500}$ 23

Computational Methodology and Details 24

Procedure 24

Effect of Neighboring OEO-based Monomers and Activation Process 24

Effect of Stereochemistry on Total Energies 26

Optimized Structure XYZ Coordinates and ΔE Values 29

References: 60

Experimental Details

Materials

All chemicals were purchased from commercial sources and used as received unless otherwise noted. Tris(2-pyridylmethyl) amine (TPMA, 99%) and tris[2-(dimethylamino) ethyl] amine (Me_6TREN, 99%) ware purchased from *AmBeed*. Eosin Y (EY, 99%), copper (II) bromide ($CuBr_2$, 99.99%), 2-hydroxyethyl 2-bromoisobutyrate (HO-EBIB, 95%), Ethyl α-bromoisobutyrate (EBIB, 98%), Triethylamine (TEA, ≥99.5%) and 1,4-bis(3-isocyanopropyl) piperazine (QA) were purchased from *Sigma-Aldrich*. 10X PBS and 2-Bromoacrylic acid (BAA, 95%) were purchased from *Thermo Fisher Scientific*. Oligo (ethylene oxide) methyl ether methacrylate (average M_n = 500, $OEOMA_{500}$), oligo (ethylene glycol) methyl ether acrylate (average M_n = 480, $OEOA_{480}$), methyl acrylate (MA) and ethyl acrylate (EA) were purchased from *Sigma-Aldrich* and passed through a column of basic alumina to remove inhibitor prior to use. Water (HPLC grade) and dimethyl sulfoxide (DMSO, ≥99.7%) were purchased from *Fisher.* 4-Cyano-4-(thiobenzoylthio)pentanoic acid (CPDAP, 97%) from Strem Chemicals. Sodium pyruvate (SP, >97%) from Tokyo Chemical Industry (TCI).

Instrumentation

Nuclear Magnetic Resonance (NMR)

^{1}H NMR and ^{13}C NMR spectra were recorded on *Bruker* Avance III 500 MHz and 126 MHz spectrometers respectively, with $CDCl_3$, D_2O or DMSO-d_6 used as the solvent.

Size Exclusion Chromatography (RI Detector- DMF or THF as an eluent)

SEC measurements of polymers were performed using PSS columns (Styrogel 10^5, 10^3, 10^2 Å) with DMF as an eluent at 50 °C and the flow rate of 1 mL/min. Linear poly(methyl methacrylate) (PMMA) standards were used for calibration. Hydrophobic acrylates like poly(methyl acrylate) and poly(ethyl acrylate) were analyzed by SEC using THF as an eluent at 35 °C and the flow rate of 1 mL/min. Linear poly(methyl methacrylate) (PMMA) standards were used for calibration.

Size Exclusion Chromatography with Multi-Angle Light Scattering (SEC-MALS- 1X DPBS eluent)

SEC-MALS measurements of zwitterionic polyacrylate and bioconjugates were performed using the Agilent SEC system (Agilent, 1260 Infinity II) coupled with MALS, DLS, UV, Viscometer, and RI detectors (*Wyatt Technology*, USA). Measurements were performed using *Waters* Ultra hydrogel Linear column with 1X DPBS as an eluent at room temperature and the flow rate of 0.5 mL/min. The absolute molecular weight ($M_{n,abs}$) is calculated using the corresponding *dn/dc* value of the polymer samples. Waters Ultra Performance Liquid Chromatography (UPLC) equipped with triple detectors (RI, MALS and Viscometer) was also used with 1X PBS as an eluent at room temperature.

Procedures

Synthesis of poly(ethylene glycol) PEG-based inibramer oligo(ethylene oxide) methyl ether 2-bromo acrylate (OEOBA)

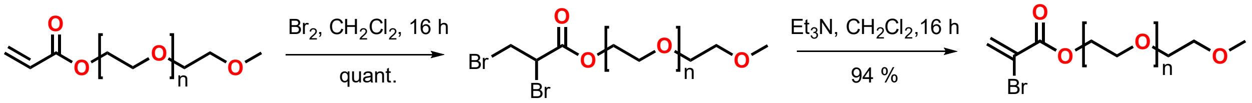

Reaction Conditions:

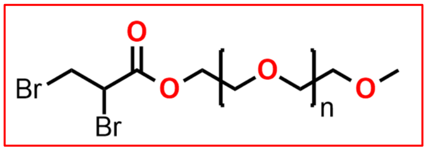

A 250 ml round-bottom flask was equipped with a magnetic stir bar and charged with oligo(ethylene oxide) acrylate (3.0 g, 6.25 mmol; M_n = 480 g/mol), followed by the addition of 100 ml of methylene chloride (CH_2Cl_2) via syringe and closed with a septum. A solution of bromine (11.0 g, 6.88 mmol; 1.1 eq.) in methylene chloride (20 ml) was prepared and added via the syringe, and the reaction was monitored by fading away of the intense bromine color resulting in an orange/yellowish solution. Reaction was left overnight, while also maintaining protection from sunlight with aluminum foil. The work-up necessitated the addition of an excess of sodium sulfite (8.0 g, 63.5 mmol) or sodium bisulfite (6.6 g, 63.5 mmol) for removal of unreacted bromine and stirring until the reaction color changed from orange to yellow. Filtering

and rinsing with methylene chloride allowed to obtain 2,3-dibromo oligo(ethylene oxide) methyl ether acrylate, which was obtained in quantitative yield and was used in the next step as a crude product (^{1}H-NMR and ^{13}C-NMR of an aliquot from reaction mixture are presented below).

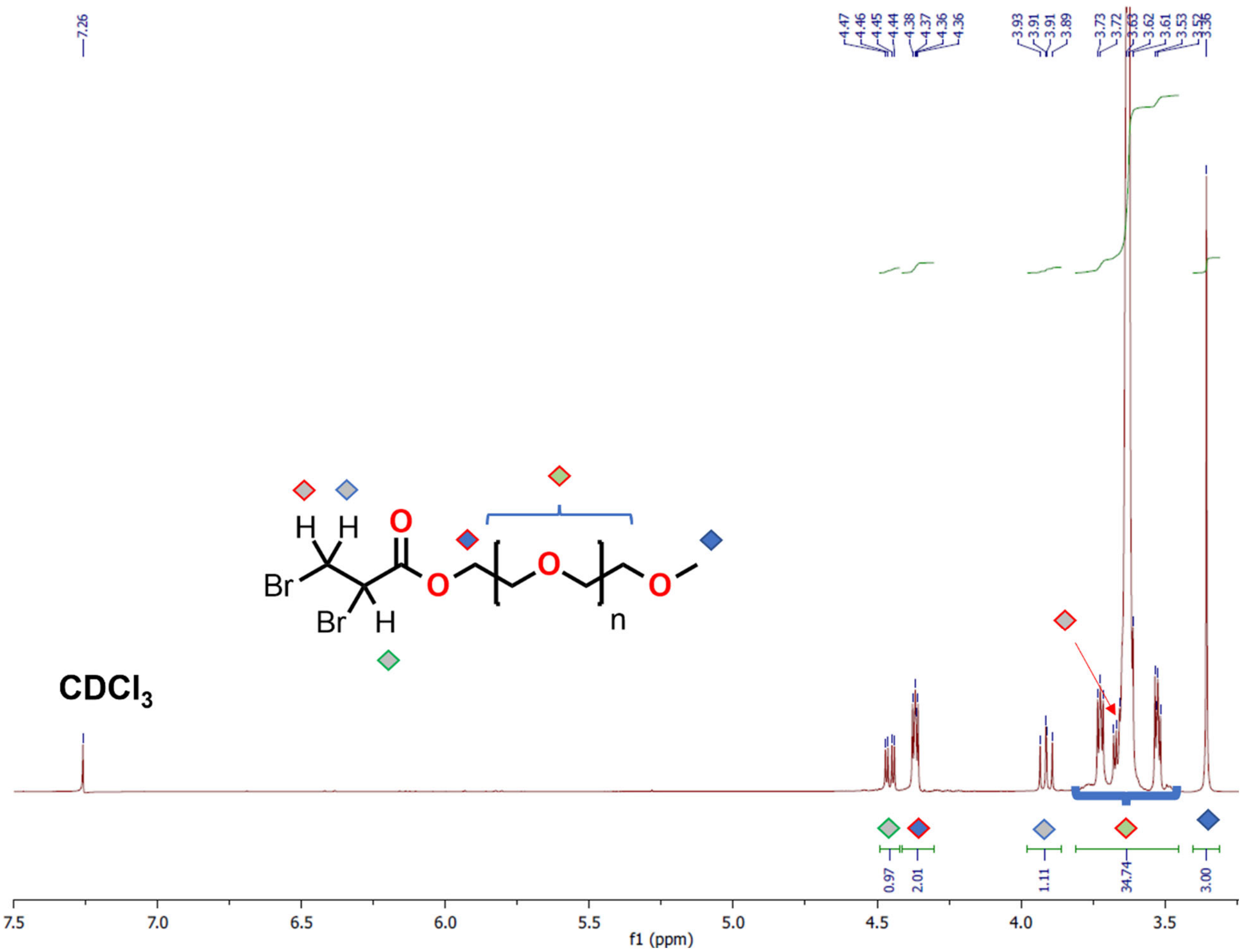

Figure S1. ^{1}H NMR of 2,3-dibromo oligo(ethylene oxide) methyl ether acrylate. ^{1}HNMR ($CDCl_3$) δ ppm 4.46 (dd, *J* = 11.2, 4.4 Hz, $BrCH_2$-, 1H), 4.37 (dd, *J* = 5.5, 4.1 Hz, -C(O)OCH_2-, 2H), 3.91 (dd, *J* = 11.1, 10.1 Hz, $BrCH_2$-, 1H), 3.81 – 3.45 (m, -O-CH_2CH_2-, 33.7H; -CHBr-, 1H), 3.36 (s, OCH_3, 3H).

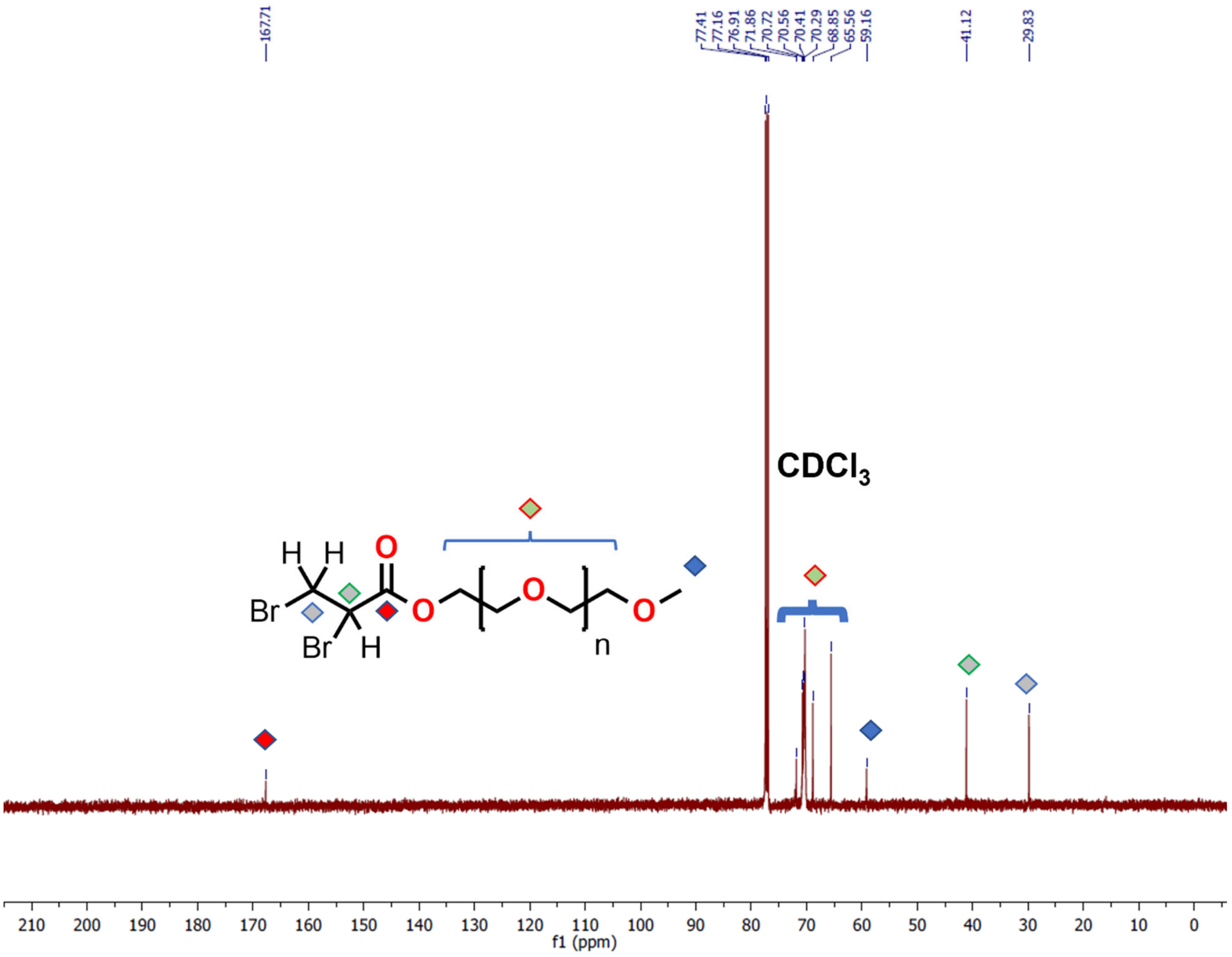

Figure S2. ^{13}C-NMR of 2,3-dibromo oligo(ethylene oxide) methyl ether acrylate. ^{13}C NMR ($CDCl_3$) δ ppm 167.7, 71.9, 70.7, 70.6, 70.4, 70.3, 68.9, 65.6, 59.2, 41.1, 29.8.

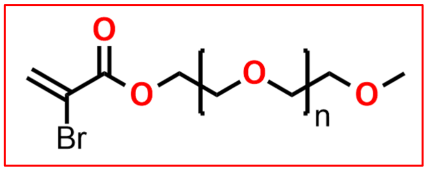

To the crude reaction mixture was added to CH_2Cl_2 so that the total volume after the previous step is 250 ml, followed by triethylamine (0.915 ml, 6.56 mmol; 1.05 eq.) and was left overnight in dark with stirring. The progress of the reaction was monitored by ^{1}H NMR (Figure S3).

The reaction mixture was filtered through a neutral alumina column using methylene chloride as an eluent to remove the $Et_3NH^+Br^-$ salt. An alternative way to remove the salt and unreacted triethylamine is to perform water/methylene chloride extraction by dissolving crude product in 200 ml of methylene chloride, extract twice with 40 mL of H_2O, drying the organic phase with sodium sulfate or magnesium sulfate,

filtering, and removal of solvent under reduced pressure. The final product oligo(ethylene oxide) 2-bromo acrylate (OEOBA) was obtained as a colorless oil (3.08 g, 5.87 mmol, 94% yield), that was stored at 4ºC. Prolonged storage can result in the monomer turning yellowish, which can then be repurified by filtering through the neutral alumina column.

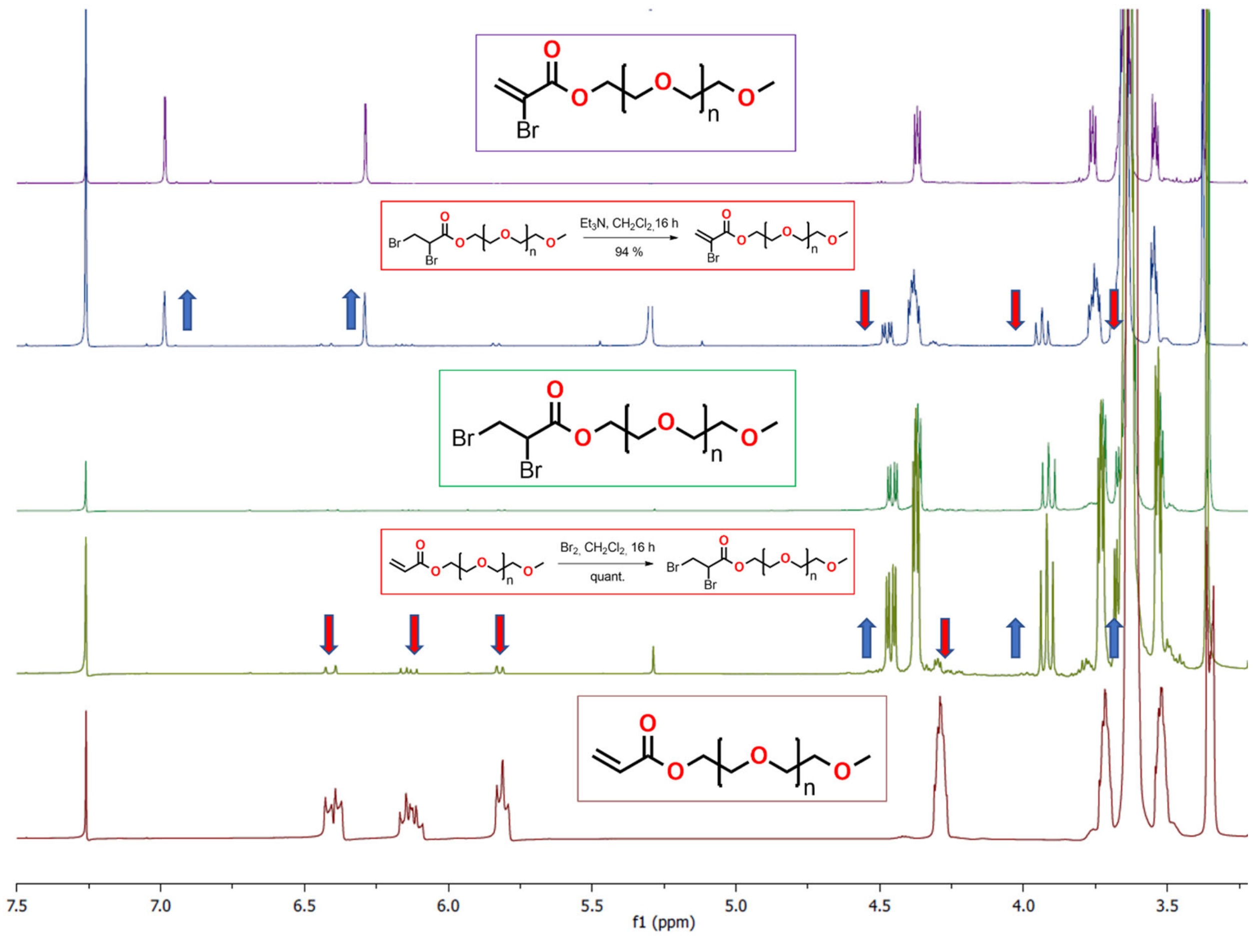

Figure S3. Comparison of the ^{1}H NMR signals in the 7.5-3.0 ppm region of oligo(ethylene oxide) acrylate starting material (bottom panel), 2,3-dibromo oligo(ethylene oxide) methyl ether acrylate intermediate (middle panel) and oligo(ethylene oxide) 2-bromo acrylate (OEOBA) final product (top panel). Signals at 7.26 ppm and 5.30 ppm correspond to $CDCl_3$ and CH_2Cl_2.

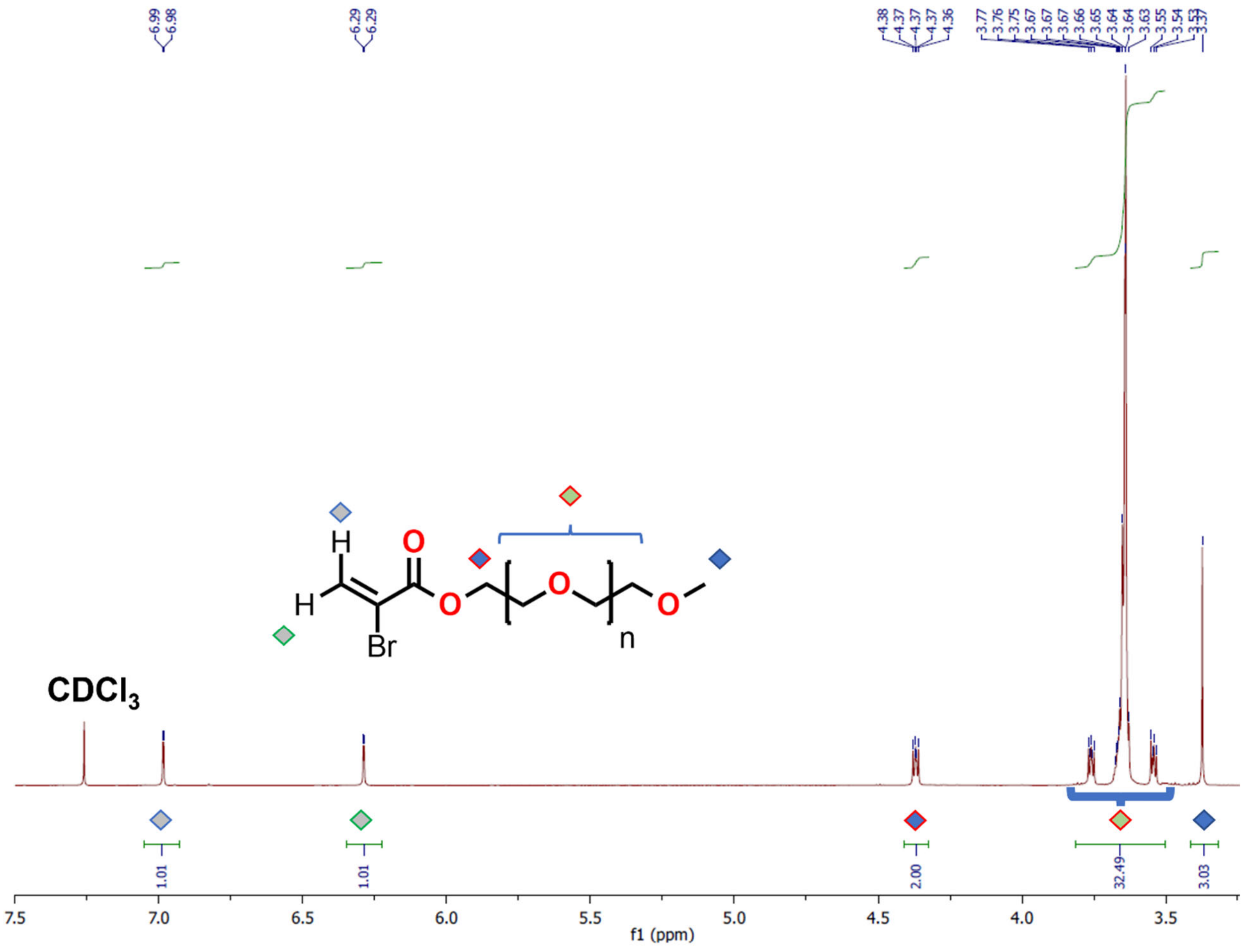

Figure S4. ^{1}H NMR of oligo(ethylene oxide) 2-bromo acrylate (OEOBA) inibramer. ^{1}H NMR (500 MHz, $CDCl_3$) δ ppm 6.98 (d, J = 1.7 Hz, H_2C=CRBr-, 1H), 6.29 (d, J = 1.6 Hz, H_2C=CRBr-, 1H), 4.48 – 4.23 (m, -C(O)OCH_2-, 2H), 3.84 – 3.45 (m, -O-CH_2CH_2-, 32.5 H), 3.37 (s, -OCH_3, 3H).

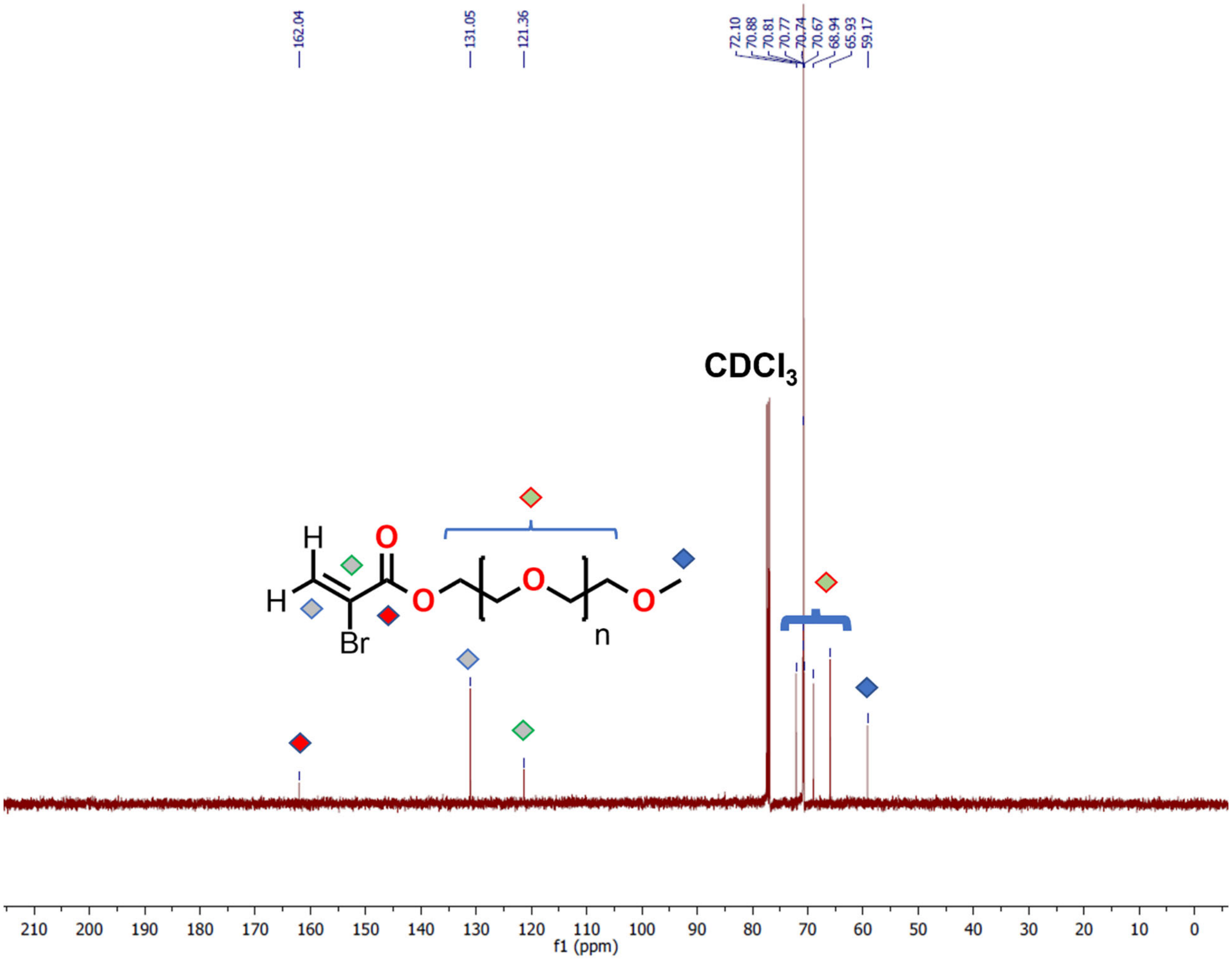

Figure S5. ^{13}CNMR of oligo(ethylene oxide) 2-bromo acrylate (OEOBA) inibramer. ^{13}C-NMR ($CDCl_3$) δ 162.0, 131.1, 121.4, 72.1, 70.9, 70.7, 68.9, 65.9, 59.2.

Synthesis of HB-POEOA$_{480}$ by copolymerization of inibramer OEOBA and OEOA$_{480}$ by EY/Cu-catalyzed ATRP

Prior to polymerization, the stock solutions of ATRP components were prepared as follows. HO-EBIB (15.8 mg in 1.0 mL DMSO), $CuBr_2$ (33.5 mg in 20.0 mL DMSO), Me_6TREN (5.18 mg in 1.0 mL DMSO), EY (0.97 mg in 1 mL DMSO) were prepared.

Batch copolymerization.

The polymerization was carried out at 2 mL volume, in an Agilent sample vial, $OEOA_{480}$ (288 mg) and OEOBA (16.5 mg) was weighed. $CuBr_2$ (80 µL), Me_6TREN (40 µL), HO-EBIB (40 µL), EY (20 µL), DMSO (20 µL), 10X PBS solution (200 µL) were then added. DMF (20 µL) was added as the internal standard and the remaining volume was filled with water. The final concentrations were $OEOA_{480}$ (300 mM), OEOBA (18 mM) HO-EBIB (1.5 mM), EY (15 µM), $CuBr_2$ (0.3 mM), Me_6TREN (0.45 mM), DMSO (10% v/v). The polymerization mixture was stirred at 100 rpm for 60 min using green LEDs 24-well plate and Lumidox box (525 nm, 50 mW/cm^2). Samples were taken and analyzed by ^{1}H NMR at regular intervals of 5 mins in D_2O to follow the conversion of monomer (Figure S6 A). SEC analysis, using 1X DPBS as the running buffer was used to obtain the absolute molecular weights ($M_{n,\ abs}$) of the final copolymer (Figure S6B).

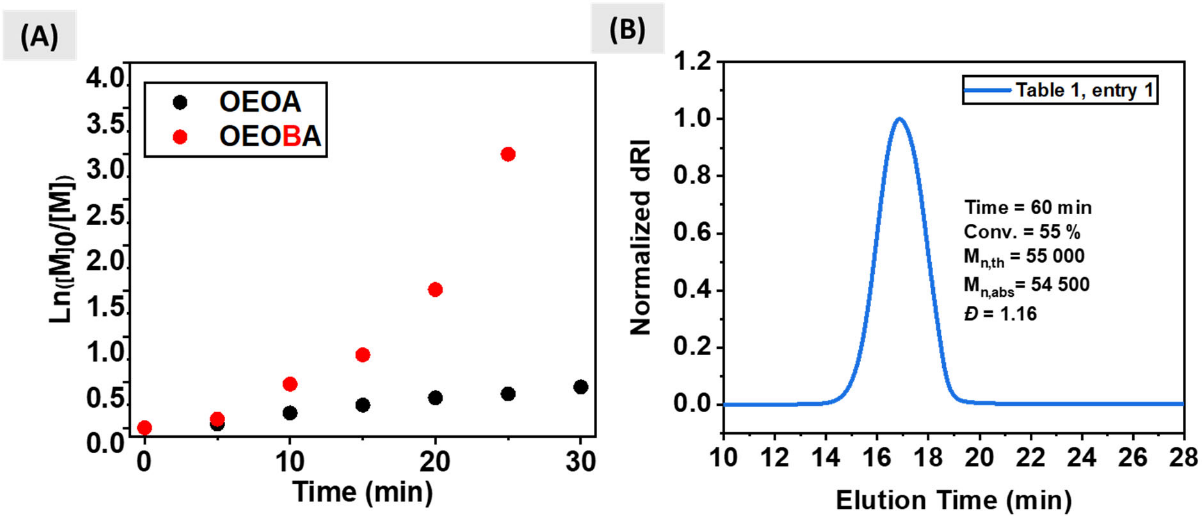

Figure S6. (A) First-order kinetic plot for one-pot copolymerization of OEOBA and $OEOA_{480}$ using molar ratios $[OEOA_{480}]/[OEOBA]/[HO\text{-}EBIB]/[EY]/[CuBr_2]/[Me_6Tren] = 200/12/1/0.01/0.2/0.3$). (B) SEC trace of the polymer after 60 min of green light irradiation (525 nm, 50 mW/cm^2) at room temperature.

Semi-batch copolymerization

The polymerizations were carried out at 2 mL volume, in an Agilent sample vial, $OEOA_{480}$ (288 mg) was weighed. $CuBr_2$ (80 µL), Me_6TREN stock (40 µL), HO-EBIB (40 µL), EY (20 µL), DMSO (20 µL), 10X

PBS solution (200 μL) were then added. DMF (20 μL) was added as the internal standard and the remaining volume was filled by water. The final concentrations were $OEOA_{480}$ (300 mM), HO-EBIB (1.5 mM), EY (15 μM), $CuBr_2$ (0.3 mM), Me_6TREN (0.45 mM), DMSO (10% v/v). To this solution, OEOBA was added using the syringe pump with a controlled flow rate as indicated in Table 1. The OEOBA concentration varied from 6 mM - 36 mM, to achieve desired degree of branching and the flow was adjusted to feed the equivalents in 60 mins. The polymerization mixture was stirred at 100 rpm for 60 min using green LEDs 24-well plate and Lumidox box (520 nm, 50 mW/cm^2). Samples were taken and analyzed by ^{1}H NMR using D_2O and SEC techniques using 1X DPBS as the running buffer to obtain $M_{n, abs}$ (Figure S7, Table 1).

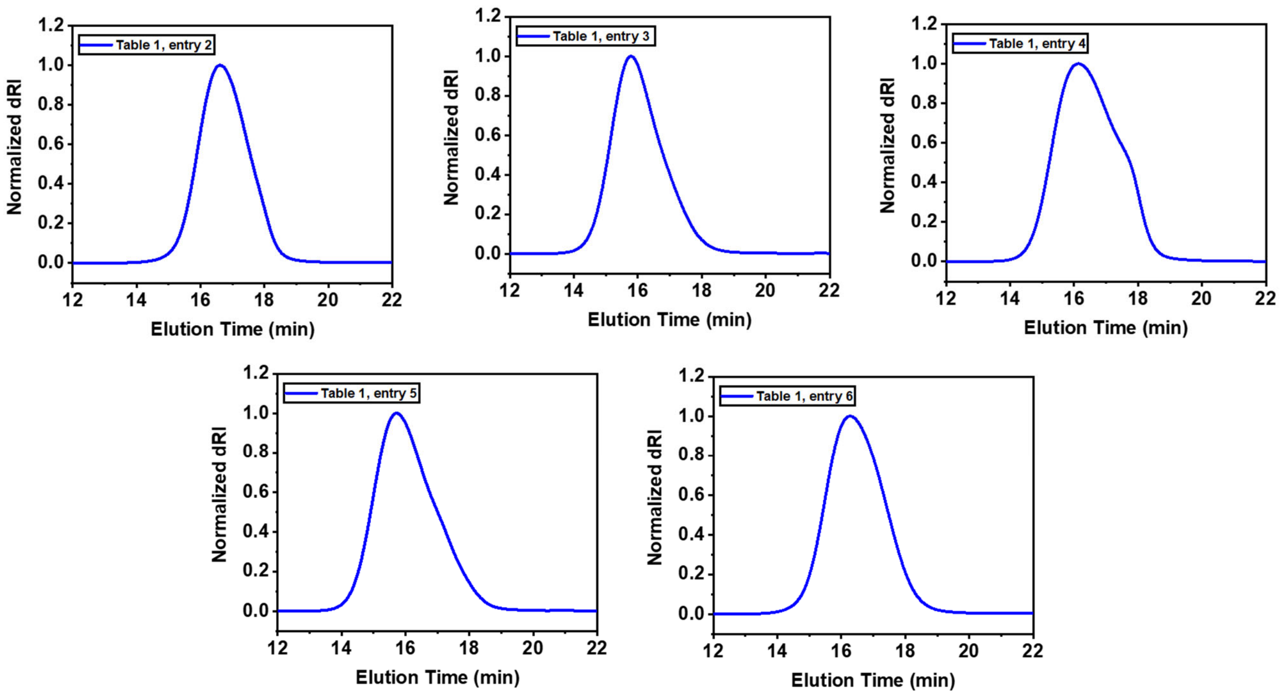

Figure S7. SEC traces of HB-$POEOA_{480}$ (Table 1, entry 2-6).

Copolymerization kinetics with slow feeding of OEOBA:

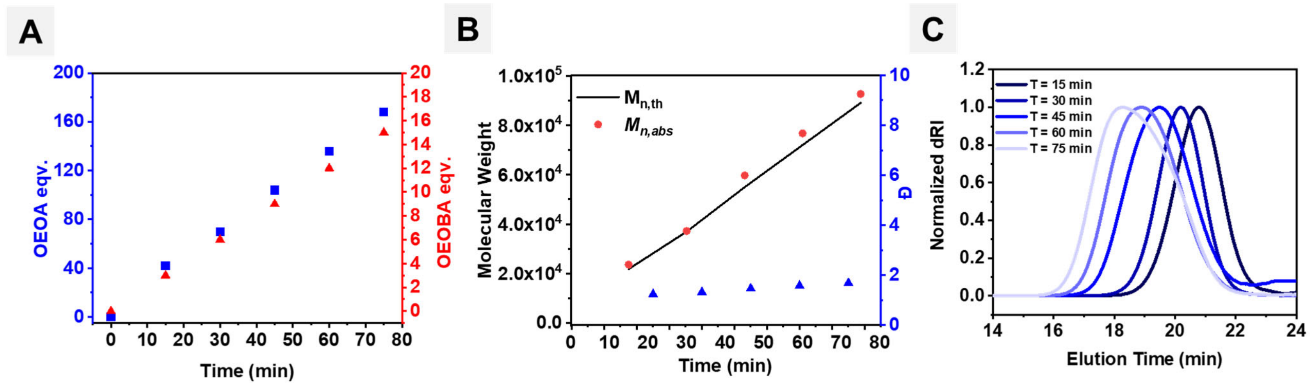

Figure S8. First-order kinetic plot for copolymerization of OEOBA and $OEOA_{480}$ with slow feeding of OEOBA for 75 minutes under green light irradiation (525 nm, 50 mW/cm^2) at room temperature. Using the molar ratios: [$OEOA_{480}$]/[OEOBA]/[HO-EBIB]/[EY]/[$CuBr_2$]/[Me_6Tren] = 200/15/1/0.01/0.2/0.3. (A) Feed equivalents over time (B) Evolution of molecular weight and molecular weight distribution with conversion (C) SEC traces evolution with time depicting shifting towards higher molecular weight region.

Expanding monomer scope

Hydrophilic acrylates such as 2-(methyl sulfinyl) ethyl acrylate (MSEA), and zwitterionic carboxy betaine acrylate (CBA) were synthesized according to previously reported procedures.[1] The linear PCBA and PMSEA were synthesized as reported earlier to compare their intrinsic viscosity with the corresponding branched polymers (Figure 2).

The copolymerization was carried out with CBA (300 mM) and MSEA (300 mM) under optimized conditions described above to reach high comonomer and inibramer conversion. The synthesized copolymers were by ^{1}H NMR using D_2O and SEC techniques using 1X DPBS as the running buffer (Figure S9 (A) and (B)).

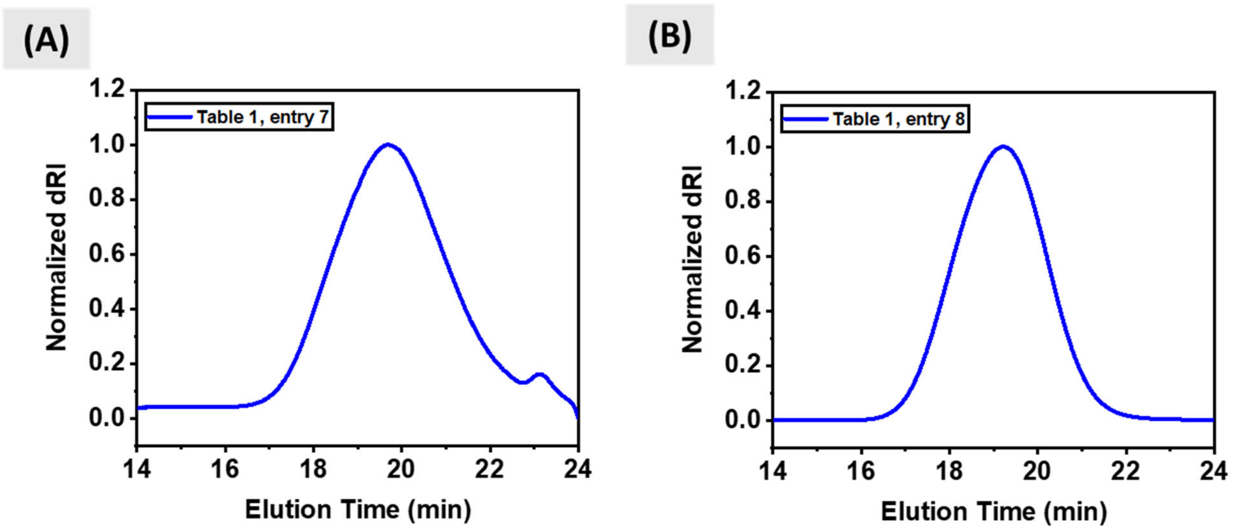

Figure S9. SEC trace of (A) HB-PMSEA (B) HB-PCBA

Extending to hydrophobic monomers- HB-PMA and HB-PEA

The linear poly(methyl acrylate) (PMA) and poly(ethyl acrylate) (PEA) were synthesized according to the previously reported procedure.[2] To introduce branching, OEOBA (18 mM) was slowly fed into the polymerization of MA or EA in a capped vial (reaction volume 2.0 mL) without any deoxygenation. The molar ratios of the components were as follows: [M]/[EBIB]/[$CuBr_2$]/[Me_6Tren]/[EY]: 200/1/0.05/0.3/0.001, [M] = [MA]/[EA] = 5.5 M, [EBIB] = 27.5 mM, in DMSO, irradiated for 90 minutes under green light LEDs (λ_{max} = 525 nm, 25.0 mW cm^{-2}), in an ambient atmosphere. The monomer conversion was determined by using ^{1}H NMR spectroscopy. The apparent molecular weight ($M_{n,app}$) was analyzed by SEC using THF as the eluent and PMMA as calibration standards. The absolute molecular weight $M_{n,abs}$ was analyzed using SEC-MALS running on THF as the eluent (Figure S10).

The resulting polymer traces revealed much lower $M_{n,app}$ for HB-PMA and HB-PEA as compared to its linear counterpart. While the $M_{n,abs}$ agreed well with the $M_{n,th}$. The HBPs also exhibited higher $Đ$ values (Figure S10).

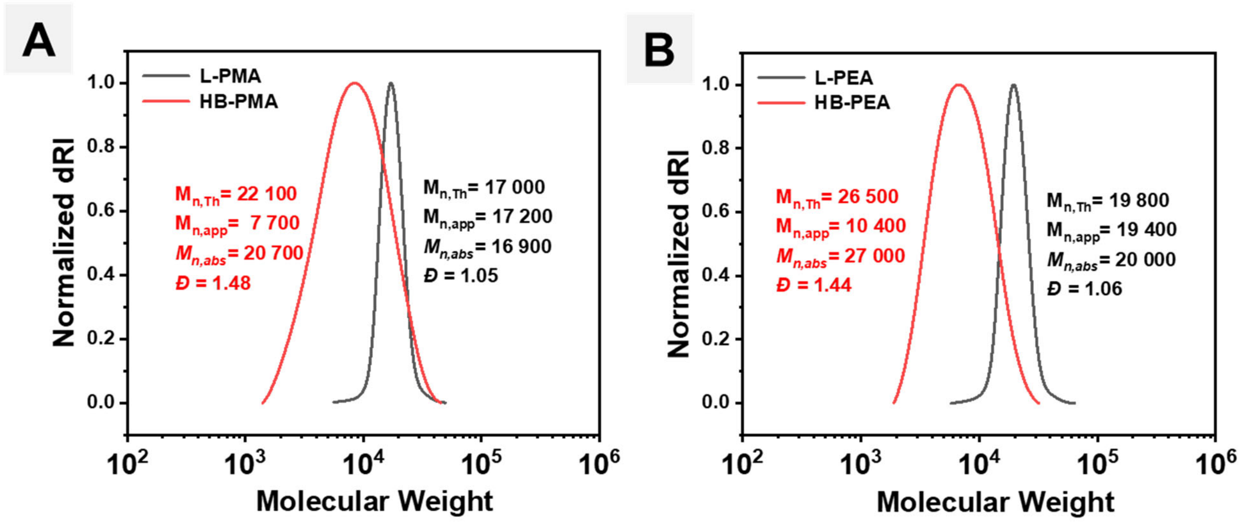

Figure S10. SEC trace of (A) HB-PMA (B) HB-PEA obtained from SEC with MALS (THF as eluent).

Varying targeted degrees of polymerization (DP)

The DP was varied in the ATRP reaction mixture prepared as described above by varying the concentration of OH-EBIB from 3 mM to 0.5 mM to synthesize HB-POEOA from DP = 100 – 600 (Figure 3A). The OEOBA was fed at the constant rate of 0.2 eq/min for 60 mins. The polymerization stock was irradiated with green light LED 24-well plate and Lumidox box (520 nm, 50 mW/cm^2), with stirring at 100 rpm. Well-defined HB-POEOA$_{480}$ of varying molecular weights were synthesized (Figure 3A).

Synthesis of topological block copolymer (L-POEOA$_{480}$ –b–HB–POEOA$_{480}$)

The first block of linear POEOA$_{480}$ (DP = 100) was chain-extended with a HB block of POEOA$_{480}$ *in-situ* without purification. The ATRP cocktail (2 mL) with a DP = 100 was prepared according to the general procedure. The final concentrations were: OEOA$_{480}$ (300 mM), HO-EBIB (3.0 mM), EY (15 µM), $CuBr_2$ (0.3 mM), Me$_6$TREN (0.45 mM), DMSO (10% v/v) and PBS. The polymerization mixture in an open-to-air vial was stirred at 100 rpm for 30 min with green LEDs (525 nm, 50.0 mW/cm^2). The macroinitiator POEOA$_{480}$ was synthesized with 80% monomer conversion ($M_{n,abs}$ = 37 500, $Đ$ = 1.06).

For the preparation of the second block, a sample (400 µL) of linear POEOA$_{480}$ (DP = 100) was used without further purification to prepare an ATRP cocktail for HB-POEOA$_{480}$ (DP = 500) as follows. In a 2 mL vial,

$OEOA_{480}$ (288 mg) was weighed and mixed with a crude $POEOA_{480}$ solution (400 µL). $CuBr_2$ (80 µL), Me_6TREN (40 µL), EY (20 µL) and 10X PBS solution (200 µL) were then added. The polymerization mixture in an uncapped vial was stirred at 100 rpm and OEOBA (36 mM) was fed at the rate of 0.2 eq/min for 60 min while irradiated with green LEDs (525 nm, 50.0 mW/cm^2). The sample was taken and analyzed by ^{1}H NMR and SEC. SEC analysis showed a clear shift toward higher molecular weights without any shoulder or tailing at lower molecular weights (conv. = 56 %, $M_{n,abs}$ = 156 100, $Đ$ = 1.58), (Figure 3B).

Synthesis of hyperbranched protein polymer-hybrid (HB-PPH)

The chymotrypsin macroinitiator (CT-$iBBr_{12}$) was synthesized according to previously reported procedure.[3, 4]

In a 2 mL volumetric flask, CT-$iBBr_{12}$ (7.0 mg) and CBA (128.5 mg, 1.5 mmol) were weighed, $CuBr_2$ (80 µL), Me_6Tren (40 µL), EY (20 µL), DMF (20 µL) as internal standard and 10X PBS solution (200 µL) were then added. Finally, water was added to the volumetric flask, and the reaction mixture was vortexed. The final concentrations were CBA (300 mM), CT-$iBBr_{12}$ (-Br sites/CT 1.5 mM), EY (15 µM), $CuBr_2$ (0.3 mM), Me_6Tren (0.45 mM), DMSO (10% v/v). Then the ATRP cocktail was transferred to Agilent vial (2 mL) equipped with a narrow magnetic stirrer. OEOBA (18 mM) was added to the polymerization mixture by slow feeding at the rate of 0.2 eq/min using a syringe pump. The polymerization mixture was stirred at 100 rpm for 60 min with green LEDs (525 nm, 50 mW/cm^2) using high-throughput set-up equipped with cooling to maintain temperature between 15-18 ºC throughout the polymerization.

Copolymerization of OEOBA and $OEOMA_{500}$ by EY/Cu-catalyzed ATRP

Prior to polymerizations, stock solutions of HO-EBIB (15.8 mg in 1.0 mL DMSO), $CuBr_2$ (33.5 mg in 20.0 mL DMSO), tris(2-pyridylmethyl) amine TPMA (13.1 mg in 1.0 mL DMSO) EY (0.97 mg in 1 mL DMSO) were prepared.

$OEOMA_{500}$ (750 mg) was added to a volumetric flask (5 mL). $CuBr_2$ (200 µL), TPMA (100 µL), HO-EBIB (100 µL), EY (50 µL), DMSO (50 µL) and 10X PBS solution (500 µL) were then added and the remaining

volume was filled up by HPLC grade water. The final concentrations were $OEOMA_{500}$ (300 mM), HO-EBIB (1.5 mM), EY (15 μM), $CuBr_2$ (0.3 mM), TPMA (0.9 mM), DMSO (10% v/v). Then the ATRP cocktail (4.4 mL) was added to a 1-dram (12/96 mm) vial equipped with a magnetic stirrer. The polymerization mixture was stirred at 500 rpm for 30 min with green LEDs (520 nm, 9.0 mW/cm^2). Samples were taken and analyzed by ^{1}H-NMR and SEC.

To introduce branching, OEOBA (16.80 mg, 6 mM) or OEOBA (50.30 mg, 18 mM) was added to the polymerization stock solutions above to get 2% and 6% OEOBA w.r.t to the $OEOMA_{500}$. Samples were taken and analyzed by ^{1}H NMR and SEC technique with DMF as an eluent calibrated using PMMA standards to obtain $M_{n,app}$ (Figure S11 A) and SEC equipped with MALS detector with 1X PBS as eluent to obtain $M_{n,abs}$ (Figure S11 B)

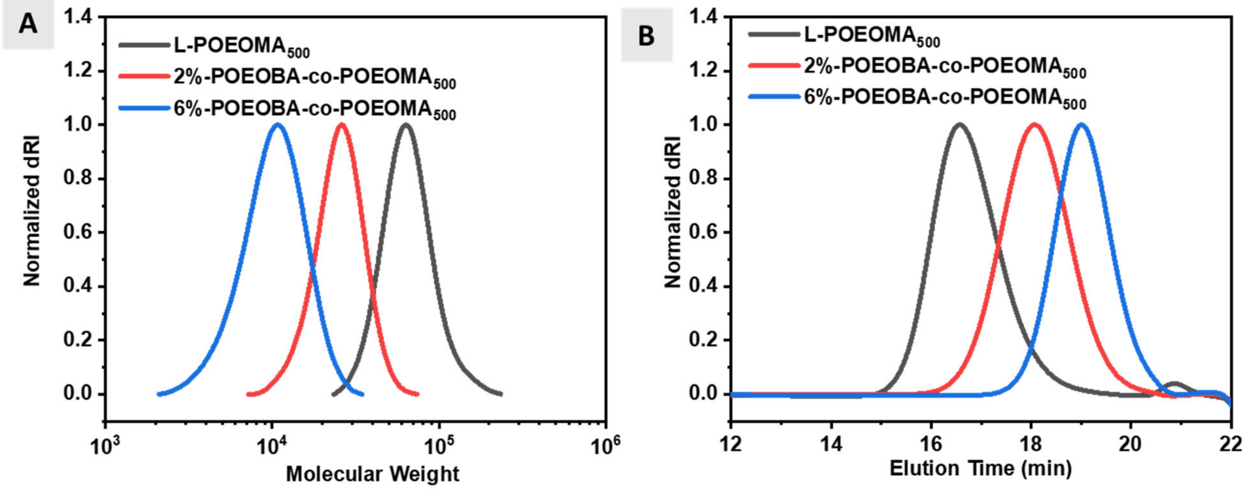

Figure S11. SEC traces for polymers presented in Table 2 (A) DMF-GPC used to determine $M_{n,app}$ using PMMA calibration standards (B) SEC-MALS used to determine $M_{n,abs}$ using *dn/dc* values.

Copolymerization kinetics of OEOBA and $OEOMA_{500}$

The stock solutions were prepared as described above with additional stock of TPMA (13.1 mg/mL) in DMSO.

In a 5 mL volumetric flask, OEOBA (50.3 mg) and $OEOMA_{500}$ (750 mg) were weighed. $CuBr_2$ (200 µL), TPMA (100 µL), HO-EBIB (100 µL), EY stock (50 µL), DMSO (50 µL) and 10X PBS solution (500 µL) were then added and the remaining volume was filled up by HPLC grade water. The final concentrations were $OEOMA_{500}$ (300 mM), HO-EBIB (1.5 mM), EY (15 µM), $CuBr_2$ (0.3 mM), TPMA (0.9 mM), DMSO (10% v/v). Then the ATRP cocktail (4.4 mL) was added to a 1-dram (12/96 mm) vial equipped with a magnetic stirrer. The polymerization mixture was stirred at 500 rpm for 30 min with green LEDs (520 nm, 9.0 mW/cm2). Samples were taken every 5 minutes and analyzed by ^{1}H NMR and SEC techniques (Figure 4).

The ^{1}H-NMR data was fitted to the Beckingham–Sanoja–Lynd (BSL) integrated model to determine reactivity ratios (Figure 4D). The BSL equation is a function of the total monomer concentration, defined by Equation S1, where $[OEOMA_{500}]_t$ and $[OEOBA]_t$ are the molar concentrations of each monomer at a given reaction time, and $[OEOMA_{500}]_0$ and $[OEOBA]_0$ are the initial monomer concentrations.

$$\text{Total Conversion} = 1 - [[OEOMA_{500}]_t + [OEOBA]_t]/[[OEOMA_{500}]_0 + [OEOBA]_0] \qquad [S1]$$

The reactivity ratios were found by fitting the experimental data to Equations S2 and S3, where $f_{1,0}$ is the initial feed fraction of $OEOMA_{500}$. The parameters r_1 and r_2 are the reactivity ratios of $OEOMA_{500}$ and OEOBA.

$$\text{Total Conversion} = 1 - f_{1,0}([OEOMA_{500}]_t /[OEOMA_{500}]_0) - (1 - f_{1,0})([OEOMA_{500}]_t /[OEOMA_{500}]_0)\text{^}r_2 \qquad [S2]$$

$$\text{Total Conversion} = 1 - f_{1,0}([OEOBA]_t /[OEOBA]_0)\text{^}r_1 - (1 - f_{1,0})([OEOBA]_t /[OEOBA]_0) \qquad [S3]$$

Analysis of POEOMA$_{500}$ fragmentation induced by Br-activated midchain radicals (MCR)

General procedure for RAFT polymerization with SP

The mixture of OEOMA$_{500}$ (750 mg, 1.5 mmol), OEOBA (50.3 mg, 90.0 µmol), CPDAP (2.1 mg, 7.5 µmol) and SP (70.1 mg, 0.63 mmol) were prepared in a volumetric flask (5 mL). After adjusting the DMSO volume to (500 µL, 10% v/v), the volumetric flask was topped up with water. The mixture was vortexed for 2 min, transferred to an open-to-air glass vial (5 mL), and irradiated with UV light (Kessil, 370 nm, 6.5 mW/Cm^2) for 1 hour to carry out the polymerization. Polymerization was stopped by turning the light off and dilution of samples in characterization solvents (DMF for GPC and D_2O for 1H-NMR).

Similar procedures were performed for carrying out polymerization of P1-24 except for the use of OEOBA (100.6 mg, 0.18 mmol).

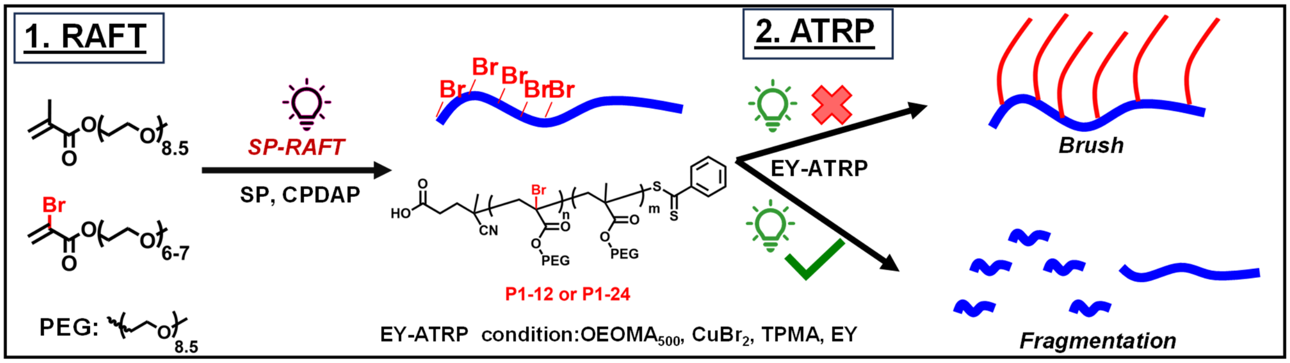

Figure S12. Synthesis of P1-12 and P1-24 by SP-RAFT polymerization of OEOMA$_{500}$ and OEOBA; dual reactivity of midchain alkyl bromides to form either brush copolymers or undergo fragmentation.

Table S1. Properties of P1-12 and P1-24 synthesized by SP-RAFT polymerization of OEOMA$_{500}$ in the presence of CPDAP.[a)]

Polymer	[OEOMA$_{500}$]/[OEOBA]	Conv. (%)$^{b)}$ OEOMA$_{500}$/OEOBA	$M_{n, theo}$ (x1000)	$M_{n, app}$$^{c)}$ (x1000)	$Đ$ $^{c)}$
P1-12	200/12	>95/>95	101.6	94.9	1.41
P1-14	200/24	>95/>95	108.0	92.0	1.42

[a] **Reactions conditions:** [OEOMA$_{500}$]/[OEOBA]/[CPDAP]/[SP]= 200/12-24/1/85 in water and DMSO (10% *v*/*v*), irradiated with Kessil LEDs (370 nm, 6.5 mW/cm^2) for 1 hour in a 5 mL glass vial. [b] Conversions were determined by using ^{1}H-NMR spectroscopy. [c] Molecular weight ($M_{n,app}$) and dispersity ($Đ$) were determined by SEC analysis (DMF as eluent) calibrated with poly(methyl methacrylate) standards.

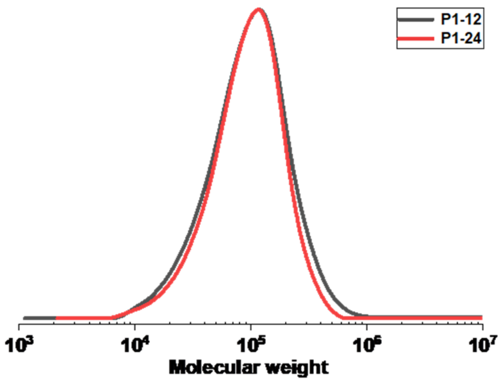

Figure S13. SEC trace of P1-12 and P1-24.

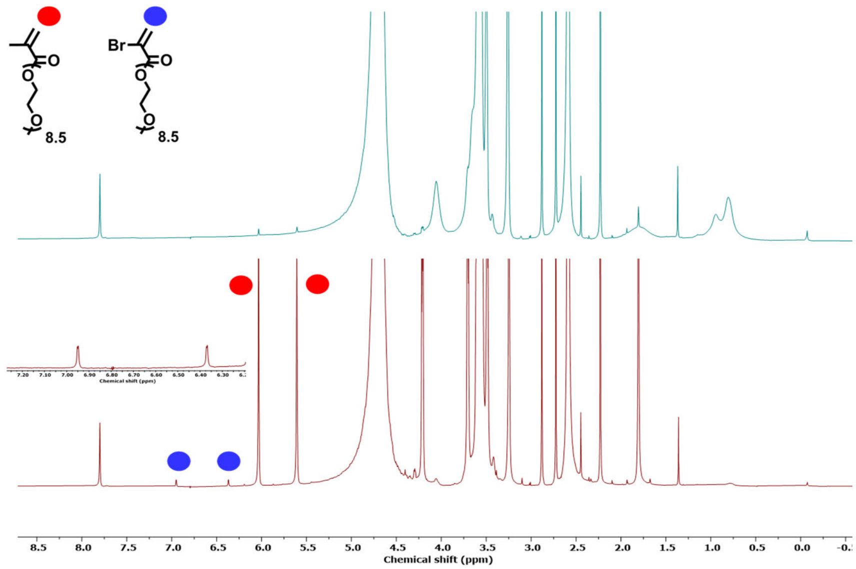

Figure S14. ^{1}H-NMR of copolymerization of $OEOMA_{500}$ and OEOBA before (bottom) and after (top) polymerization suggests the consumption of both monomer and inibramer.

General procedure for fragmentation experiments

P1-12 fragmentation without monomer was carried out by mixing P1-12 (27.5 mg, 0.25 µmol), $CuBr_2$ (67.0 µg, 0.30 µmol), TPMA (0.26 mg, 0.90 µmol), EY (9.7 µg, 0.015 µmol), PBS (100 µL), DMSO (100 µL) in a volumetric flask (1 mL). The mixture was vortexed for 2 minutes and then transferred to an open cap glass vial which was irradiated with green light for different times.

P1-12 and P1-24 fragmentation in the presence of $OEOMA_{500}$ (300 mM) was carried out by mixing P1-12 or P1-24 (55 mg, 0.50 µmol), $OEOMA_{500}$ (300 mg, 0.6 mmol), $CuBr_2$ (0.13 mg, 0.60 µmol), TPMA (0.52 mg, 1.8 µmol), EY (19.4 µg, 0.03 µmol), PBS (100 µL), DMSO (200 µL) in a volumetric flask (2 mL). The mixture was vortexed for 2 minutes and then transferred to an open cap glass vial which was irradiated with green light for different times.

All the fragmentation experiments were carried out without prior deoxygenation unless otherwise stated. For SEC analysis, aliquot of samples was withdrawn (250 µL) and the solvent was evaporated by Biotage solvent evaporator. The polymers were redissolved in DMF and analyzed by SEC.

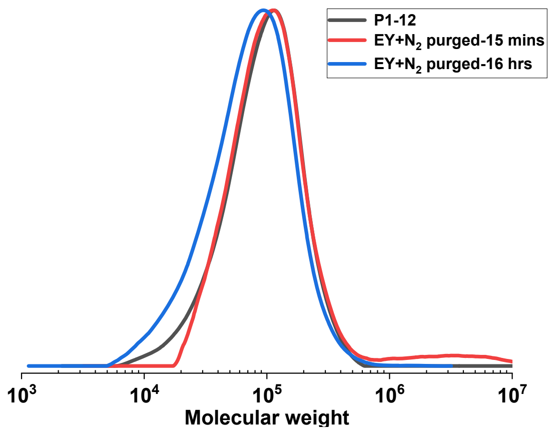

Figure S15. SEC traces of P1-12 compared to products after treatment with EY and green light (without $CuBr_2$ and TPMA) after 15 minutes or 16 hours light irradiation.

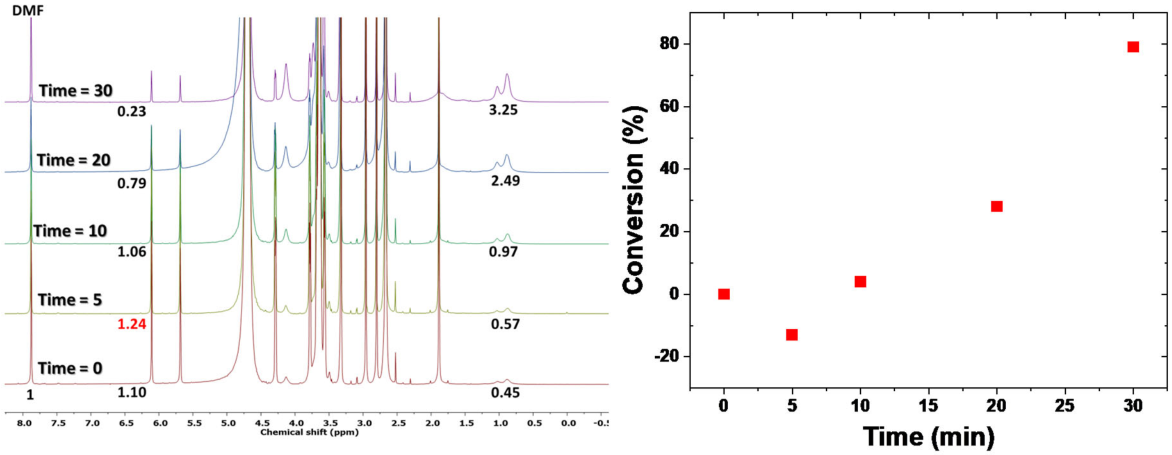

Figure S16. Overlapped ^{1}H-NMR of P1-12 activation under EY/Cu ATRP condition in the presence of $OEOMA_{500}$ (left) and conversion versus time plot for the polymerization (right). Values under spectra represent integration of vinyl and PMMA (CH_3) peaks with respect to DMF.

Attempts to suppress fragmentation in copolymerization of OEOBA and OEOMA$_{500}$ by low temperature polymerization and slow feeding.

Table S2: Copolymerization of OEOBA and OEOMA$_{500}$ at low temperature and slow feeding of OEOBA

Entry	Temp.	Feeding (OEOBA)	Time (min)	α_M (%)[a]	α_{IB} (%)[a]	$M_{n,th}$[b] ([I])	$M_{n,abs}$[c]	$Đ$[c]
1.	25℃	-	30	72%	>99	73 000	18 000	1.17
2.	25℃	Yes	30	67%	-	68 000	22 300	1.19
3.	12℃	-	30	26%	55%	26 600	18 400	1.20
			60	75%	99%	76 000	18 300	1.14
			90	86%	99%	87 000	18 600	1.15
4.	12℃	Yes	30	35%	-	35 000	23 000	1.17
			60	69%	-	69 000	26 000	1.19
			90	78%	-	78 000	24 000	1.23
5.		1.66 uL/min	60	12	-	12 000	-	-
	< 5 ℃		120	48	-	49 000	17 400	1.14
			180	84	-	85 200	18 200	1.16

Reaction Conditions: [$OEOMA_{500}$]/[OEOBA]/[HO-EBIB]/[EY]/[$CuBr_2$]/[TPMA]: 200/12/1/0.01/0.2/0.6. [$OEOMA_{500}$] = 300 mM. [OEOBA] = 18 mM, [HO-EBIB] = 1.5 mM in 1X PBS buffer at room temperature, irradiated with green light LEDs (λ_{max} = 520 nm, 100 mW/cm^{-2}), in an open-to-air vial. Reaction volume was 4.4 mL, stirring at 100 rpm. [a]$OEOMA_{500}$ and OEOBA conversion was determined by using ^{1}H NMR spectroscopy. [b]Theoretical molecular weight was calculated using the equation $M_{n,th}$ =[M/I] * MW_M *αM + [OEOBA/I]* MW OEOBA* αIB +MW_I. [c]$M_{n,abs}$ and $Đ$ analyzed using SEC (1X PBS as eluent) equipped with triple detectors: Multi-angle light scattering (MALS), Refractive index (RI) and inline viscometer and UV detector.

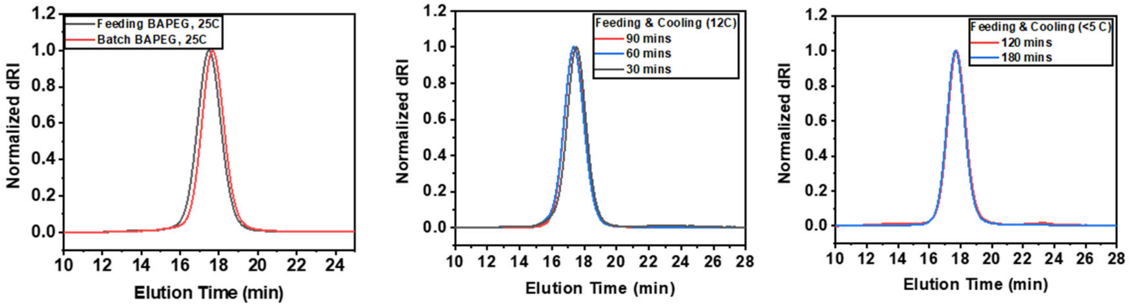

Figure S17. SEC-MALS analysis of copolymers synthesized in Table S2.

Comparison between OEOBA and SBA during copolymerization with $OEOMA_{500}$

Linear $POEOMA_{500}$ (M_n = 20 000) was synthesized by EY/Cu catalyzed ATRP according to previously reported procedure and used as macroinitiator (macro-I) for subsequent chain-extension.[2] The macro-I was mixed without any purification with $OEOMA_{500}$ and OEOBA or SBA. In reaction mixture 1, OEOBA (18 mM) was added in one-step and in the other reaction mixture, SBA (18 mM , BAA/Na_2CO_3 = 2.25/1) was introduced. The final concentrations were as follows: [$OEOMA_{500}$]/[IB]/[Macro-I]/[EY]/[$CuBr_2$]/[TPMA]: 250/15/1/0.012/0.25/0.75. Both the polymerizations were conducted at 4.4 mL scale, stirring at 500 rpm under green LED lights light LEDs (λ_{max} = 520 nm, 9 mW cm^{-2}) at ambient temperature for 30 minutes. ^{1}H NMR and UPLC with triple detectors were used to analyze the polymerization products (Figure 6).

Computational methodology details

Procedure

The structures of different alkyl halides were studied using the same computational procedure to calculate electronic energy differences between fragmentation and radical formation. Initially, the chemical structures were created based on a workflow developed in Mathematica 13.3, using image recognition of molecules, generating 3D geometries based on 2D drawings. This workflow differentiated between radicals, anionic species, and enforced stereochemistry. In cases without designated stereochemistry, stereocenters were designated as below. All structures were optimized with ORCA 5.0.4, using Density Functional Theory. The functional of choice was ωB97X, with D4 dispersion correction, def2-TZVP basis set and convergence tolerance for optimization of 10^{-12}. Vibrational frequencies were also checked for consistency and no imaginary modes were found, confirming the quality of the optimized structures. Final single-point energies were calculated on the optimized structures with DLPNO-CCSD(T) level of theory, using def2-TZVP as the basis sets. All calculations were carried out without solvent corrections, considering molecules in vacuum.

Effect of neighboring OEO-based monomers and activation process

β-methoxyethyl esters flanking the β-methoxyethyl inibramer unit (i.e. R = R_1 = -$CO_2CH_2CH_2OMe$) decreased ΔΔE further by ~4.2 kcal/mol when going from R = -$CO_2CH_2CH_2OMe$ with R_1 = -CO_2Me to R = R_1 = -$CO_2CH_2CH_2OMe$ (Figure 7, main text). However, this was only observed in the methacrylate system. The analogous acrylate system did not exhibit this tendency and instead exhibited a ΔΔE value ~6.3 kcal/mol higher when going from R = -$CO_2CH_2CH_2OMe$ with R_1 = -CO_2Me to R = R_1 = -$CO_2CH_2CH_2OMe$. This highlights a delicate interplay of electronic effects related to substituents on neighboring esters alongside their steric contributions that would require further investigations beyond the scope of this work to comprehensively isolate and compile.

It generally followed for both acrylate and methacrylate models that ΔE_1 for R = -CO2$^-$ was higher than for -$CO_2CH_2CH_2OMe$, which was only marginally higher than for -CO_2Me. This may indicate that the total tendency for midchain halide positions bearing R = -CO_2^- to undergo midchain activation may be lower than corresponding neutral esters, given the same catalyst and medium for activation. However, midchain alkyl halides α− to an ester or carboxylate, which become *de facto* tertiary initiators upon incorporation into the chain, can be considered readily activatable by Cu-ATRP catalysts of even intermediate activity.[5, 6] Furthermore, given the irreversible nature of addition or β-scission reactions subsequent to activation, the magnitude of ΔE_1 should not be considered prohibitive to branching or fragmentation chemistry occurring at the midchain alkyl halide. To this end, bulkier macromonomer neighboring units would not be anticipated to bear any meaningful significance regarding the feasibility of alkyl halide activation.

It is important to note that a positive ΔE_1 value does not mean that the MCR-Br species cannot be activated. Within the catalytic system, Cu^I-activator complexes are involved with the alkyl halide in an inner-sphere electron transfer (ISET) reaction.[7] The ISET results in the halogen unit being abstracted to form Cu^{II}-deactivator complexes, catalyzing the activation process. The activation process itself is not of concern to this investigation, as it was already reported experimentally for inibramer units to branch or fragment when built into the backbone under Cu-ATRP conditions.

Furthermore, the interaction of tertiary MMA-like radicals with Cu^I-activators to form Cu^{II}-alkyl organometallic species was quantified to be generally insignificant, especially when considering polymerization scale.[8] This indicates that the employed Cu^I-activators would not efficiently associate with the studied MCRs. Furthermore, without association, catalytic radical termination at MCR sites would never proceed. To this end, it is not necessary to consider Cu-catalyst explicitly for activation-scission modelling in the considered structures or as inducing meaningful proportion of Cu-catalyzed side reactions. Rather, Cu-catalyst should be eschewed for simplicity; indeed, the β-scission step itself is agnostic of Cu-catalyst. However, the exact role of Cu-ATRP complexes on other inibramer-containing copolymers or

when in the presence for MCRs in general should not be disregarded depending on the units of the copolymer.

Effect of stereochemistry on total energies

While the methacrylate substrates employed in computational investigation do not possess the capacity for stereochemical configuration, acrylate model stereocenters could impact total energies. It was assumed that for acrylate models, synthesized copolymers realistically would contain all possible stereochemical configurations neighboring their corresponding inibramer units, but their tendencies should all be similar (assumption 3, main text).

To this end, a pilot model study for the effect of stereochemistry on the total energies of MCR and fragmentation products was performed; the chosen substrate was for OEOBA-like inibramer unit flanked by methyl acrylate-like units with asterisks (*) labelling the stereocenters (Figure S17). OEOBA-like inibramer was chosen for the reason that it was found to stabilize fragmentation products in the methacrylate case. However, the acrylate model with OEOBA-like inibramer flanked by $OEOA_{480}$-like monomer units actually had higher ΔΔE than with merely methyl acrylate-derived monomer. Thus, it was deemed arbitrary which "monomer" unit would show the greatest impact from stereochemistry.

Generally, it was regulated for acrylate model fragmentation calculations of ΔE/ΔΔE to include MCR-Br, MCR, and unsaturated fragment (U) units possessing corresponding stereochemical configuration. In other words, *R*,*R* diastereomers should not fragment into *S*-containing U species. Due to symmetry, it was deemed arbitrary whether *R*,*S* substrates fragment into either *R* or *S* units for U. Radical fragments (RF) fragmenting from the acrylate model would possess no absolute stereochemical capacity owed to the sp^2 hybridization of the carbon-centered radical.

Table S3. Stereochemical configurations used in the main-text acrylate model for its two MCR stereocenters labeled by an asterisk (*), e.g., RS indicates *R*,*S* configuration, and the single stereocenter used for the corresponding unsaturated fragment (U).

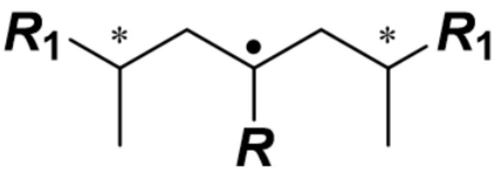

	$R_1 = -CO_2Me$<br>$R = -CO_2Me$	$R_1 = -CO_2Me$<br>$R = -CO_2CH_2CH_2OMe$	$R_1 = -CO_2Me$<br>$R = -CO_2^-$	$R_1 = -CO_2CH_2CH_2OMe$<br>$R = -CO_2CH_2CH_2OMe$
MCR	RS	SR	SS	SS
U	R	S	S	S

Stereochemistry had little impact on the overall trend associated with the acrylate model which showed little stabilization of fragmentation products and thus no tendency to fragment experimentally. Indeed, going from the SR configuration (which was reported in the main text) to the analogous RR resulted in an increase of ~0.6 kcal/mol for ΔΔE (Figure S17). Such a meager change in ΔΔE for the diastereomer, while intriguing from an organic-physical perspective, is not of high enough magnitude to appreciably change direction and reverse any trends observed for branching vs. fragmentation within the acrylate model. Lastly, any *R*,*R* configuration would be otherwise identical energetically to analogous *S*,*S*.

While a comprehensive analysis of all diastereomers achievable by an acrylate model, especially in the context of higher *n*-unit substrates like pentamers, heptamers, etc. would be intriguing for their modelling, such full analysis becomes much more computationally expensive and beyond the scope of this work alone. However, it is worthwhile in a comprehensive investigation for the fate of MCRs, especially in an expanded library of copolymers.

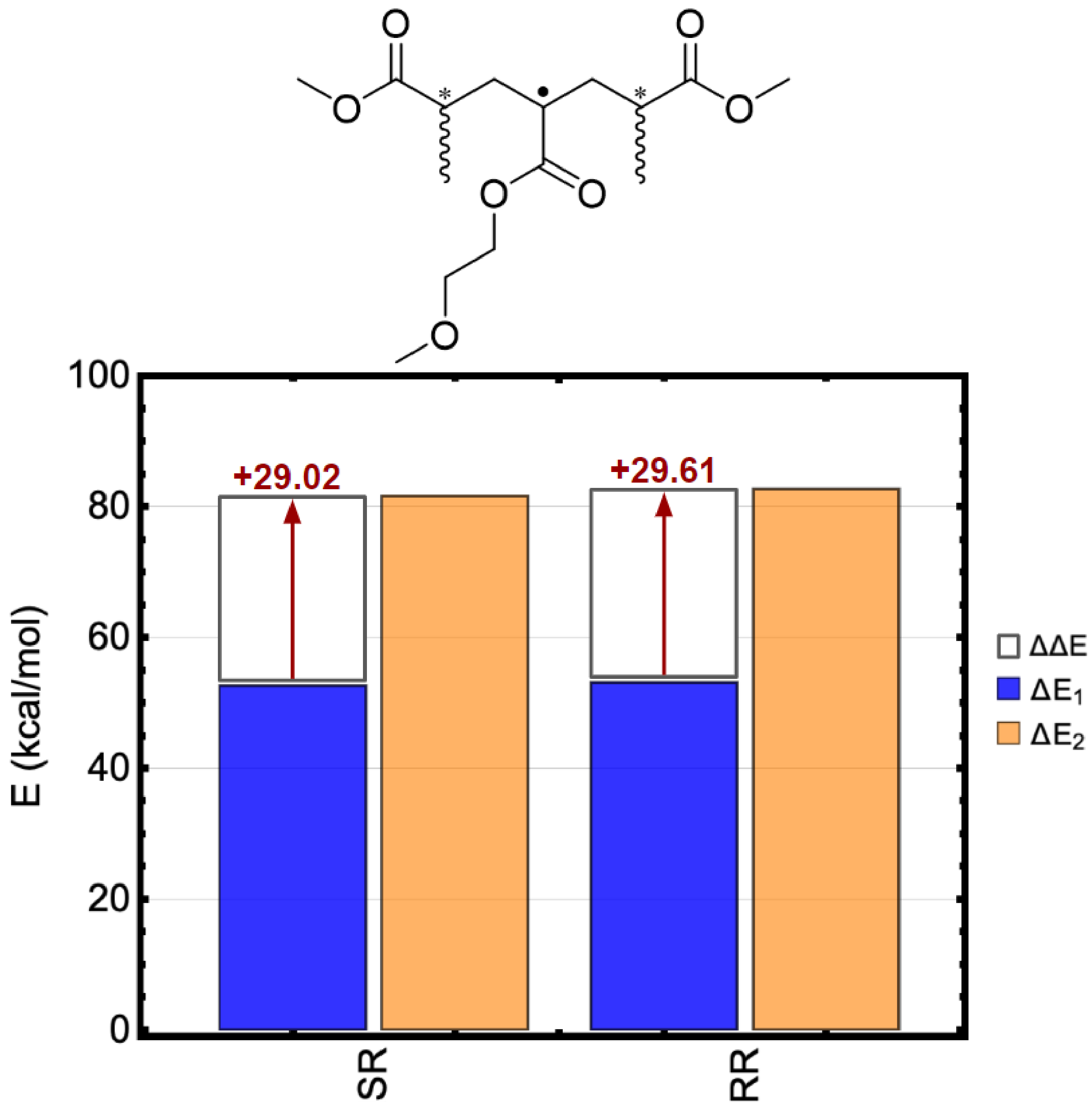

Figure S17. Corresponding ΔE_1, ΔE_2, and $\Delta\Delta E$ energies for (methyl) acrylate model substrate with OEOBA-like inibramer unit, specified with SR (*S*,*R* which was reported in main text) and analogous *R*,*R* diastereomer (RR: ΔE_1 = 53.09 kcal/mol, ΔE_2 = 82.70 kcal/mol). Values obtained at the ωB97X-D4 def2-TZVP level of theory.

Optimized Structure XYZ Coordinates and ΔE Values

For reference, the total energy differences for activation and β-scission are summarized below in Table S3. The XYZ coordinates of optimized structures are presented below (atomic Br not included).

Table S3. The ΔE_1 and ΔE_2 values presented in Figure 7 (main text) for acrylate and methacrylate models with different IB and comonomer structures.

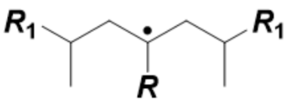

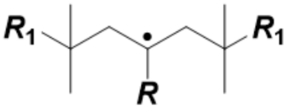

	Acrylate Model (kcal/mol)				Methacrylate Model (kcal/mol)			
	$R_1 = -CO_2Me$<br>$R = -CO_2Me$	$R_1 = -CO_2Me$<br>$R = -CO_2CH_2CH_2OMe$	$R_1 = -CO_2Me$<br>$R = -CO_2^-$	$R_1 = -CO_2CH_2CH_2OMe$<br>$R = -CO_2CH_2CH_2OMe$	$R_1 = -CO_2Me$<br>$R = -CO_2Me$	$R_1 = -CO_2Me$<br>$R = -CO_2CH_2CH_2OMe$	$R_1 = -CO_2Me$<br>$R = -CO_2^-$	$R_1 = -CO_2CH_2CH_2OMe$<br>$R = -CO_2CH_2CH_2OMe$
ΔE_1	56.1365	52.6124	65.3944	49.0261	49.5509	48.6488	62.4214	70.6949
ΔE_2	80.2103	81.6349	91.6643	84.3902	70.5742	36.0127	87.5484	53.8747

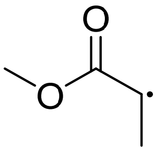

13

C	-0.01372805402020	0.06292195794230	-0.00000508569129
O	-1.10494881702124	-0.73013099311739	-0.00000116521562
C	1.21200889896702	-0.70589878756853	-0.00000283228627
C	-2.35380785421636	-0.04509707221136	0.00000049799158
O	-0.07786362992965	1.27369624374534	-0.00000146229423
C	2.53287514066765	-0.04598426067821	0.00000009987565
H	1.12879191892431	-1.78541862170936	-0.00000266902814
H	-3.11748627238988	-0.81870404043859	0.00000541030475
H	-2.44698262135293	0.58287022168392	-0.88640801468338
H	-2.44697737961194	0.58287624466281	0.88640525094407
H	3.11808086768892	-0.34380256741515	0.87614300775783
H	3.11808574706710	-0.34380427498885	-0.87613890066523
H	2.42141205522720	1.03707095009308	-0.00000113700973

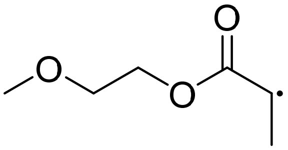

20

C	-1.31849462400009	-0.04075032888032	-0.17119885852945
C	-2.67112091339102	-0.41256259970437	0.18084743988247
C	0.85563908676165	-0.86468456901670	-0.58724781664501
O	-0.50484946981581	-1.11797312288299	-0.23387544608905
O	3.00304639314580	-0.20861721011523	0.29508407474443
C	1.66439175403740	-0.46733688802297	0.64002976956200
C	3.22480647210111	1.11149151266994	-0.14601828649867

O	-0.94304354952363	1.09171614672130	-0.39058527040914
C	-3.74103811503997	0.59720831249253	0.30351531246644
H	-2.87520893105443	-1.46240619681389	0.34916065207400
H	0.89754165248893	-0.08485343195823	-1.34833488999758
H	1.23396138696440	-1.79858571390594	-1.00030281726419
H	1.20423257421394	0.40647857170483	1.11776989288013
H	1.66699836760923	-1.29113715035755	1.35680906248991
H	4.27742978941989	1.18866455545595	-0.41401122930596
H	3.00206516891441	1.83349669440805	0.64871941748446
H	2.61640643977994	1.36453008234726	-1.02156234144200
H	-4.20880400269262	0.55210412646142	1.29199958443571
H	-4.53876475296546	0.40679661386744	-0.42242014440061
H	-3.34712772695367	1.59876859552946	0.13903289456211

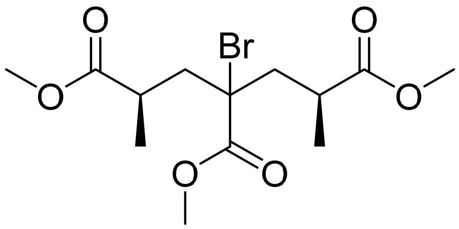

41

C	1.45274611104882	0.13100877677478	0.41364651021466
C	0.01955741236428	-0.27403370093103	0.03177982718602
C	-0.69712688066107	-0.76985841544708	1.29161312628784
O	0.07473564611614	-1.57326710164507	2.02163229138685
C	-3.11219453989698	1.29651043975090	0.00353401066286
O	-4.28245076364719	0.65710064736899	0.09608935206514
C	3.49715230316398	1.44791959171496	0.16372649890796
O	4.41458610618731	0.47969377059062	0.28960056803162
C	2.26421937555093	0.97407680125078	-0.58293154657459
C	-0.52985243480965	-2.10728876767126	3.20121755893585
C	-5.20304115842870	1.20588079759092	1.03712128701330
O	-2.85987883092527	2.30579868910117	0.60784143598218
O	3.63744057335559	2.55048121583256	0.62184254641891
C	5.59111188045297	0.83515616475163	1.01663703121491
O	-1.81648497814741	-0.46650085846574	1.60278353401776
C	-2.20543238890035	0.64383104611185	-1.01981544520050
C	-0.73023154746391	0.85707618484694	-0.66620489654085
C	2.63553767766259	0.26995038286334	-1.88533603207047
C	-2.52698518884496	1.25380816936766	-2.38810677722084
Br	0.03025240325523	-1.86186703286143	-1.14981792279087
H	2.01569782475498	-0.76221067117323	0.68353904859922
H	1.35291606843044	0.72544885324983	1.32978343675575
H	1.71875490371863	1.89284820084517	-0.80196151197405
H	-0.82094575429152	-1.30273866155432	3.87576868220517
H	0.22759944150930	-2.73666988187828	3.65979879972628
H	-1.41067033838698	-2.69392361245611	2.94159797777631
H	-6.09078877743908	0.58068033756205	0.98617233222277
H	-5.44591974837584	2.23751347767733	0.78215023929556
H	-4.77326388417453	1.17972311350369	2.03847598071565
H	6.21766598557304	-0.05283261215431	1.01792995077716

H	5.33554680863182	1.12570171140313	2.03551498268533
H	6.10203152779171	1.66549261296692	0.53008400811871
H	-2.45169940462218	-0.41761903897505	-1.04720839827985
H	-0.65630349421918	1.74780484464542	-0.03361191374798
H	-0.18095538966667	1.07606862651745	-1.58278702502179
H	3.31467141952000	0.89188094084163	-2.47142757887826
H	3.12533236086638	-0.68472373964382	-1.69358043102294
H	1.75186609246310	0.07737732688051	-2.49245303482573
H	-2.27113972456539	2.31573673371713	-2.40509093184467
H	-3.58692752183158	1.15125209659295	-2.62471831139374
H	-1.95345817311879	0.74790854053643	-3.16676925981663

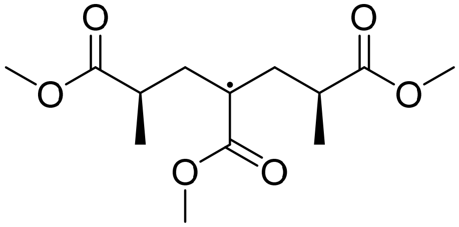

40

C	-1.26598678981170	-0.15972571165649	-0.82966864493691
C	0.20795679895120	-0.41400870051831	-0.89093832769417
C	0.78884747128054	-1.58961272554587	-0.25889108435443
O	2.12508888534906	-1.66520236996548	-0.43859502672699
C	2.74443346839120	1.22161761624884	-0.08402303879151
O	2.18932594737093	0.96356042455750	1.11004056546655
C	-3.48210616964305	-0.18665764345774	0.37145677499924
O	-3.75399874089063	0.92583456934946	-0.33305960601875
C	-1.99691473301291	-0.46006012565836	0.48531944734563
C	2.76321837101017	-2.79088469971094	0.15640458402620
C	3.07807561854249	0.45208117501618	2.10185271453108
O	3.91833544796922	1.09274526544531	-0.30475447917504
O	-4.34027349606423	-0.85336334471845	0.88463766834005
C	-5.13589246298030	1.26778269809587	-0.43067418395329
O	0.17590358894730	-2.43425941639935	0.35994398252694
C	1.72335638834949	1.67724157330289	-1.10629001516726
C	1.04913532870452	0.45193097853539	-1.77622298734488
C	-1.44884183879227	0.34548795853844	1.66528921829874
C	0.72564134757626	2.68057358518325	-0.53983498057602
H	-1.44342486630644	0.88199755573241	-1.10487369124690
H	-1.73483050451374	-0.75293883123924	-1.62929622454884
H	-1.90848876250276	-1.51817075019980	0.71803756400073
H	2.38698422783633	-3.71856185279647	-0.27516319111485
H	3.82359150081800	-2.67306324157909	-0.05354152520106
H	2.58327468994504	-2.81091634430118	1.23219272191381
H	3.54186413997013	-0.46995417406532	1.75056865718963
H	2.46783277707995	0.26379916108427	2.98172855705510
H	3.85912197380006	1.17856803677046	2.32503269490092
H	-5.68949595043356	0.47307758676152	-0.93041596811419
H	-5.17474529376798	2.18482319719190	-1.01304553198437
H	-5.56195027167417	1.42411193126007	0.56017540537517
H	2.31420001096914	2.15878229595823	-1.88746918561788

H	1.83518353620282	-0.14572067659739	-2.23926577281587
H	0.41533481666455	0.84384418894024	-2.57751316300103
H	-1.94565853476687	0.05042251944864	2.59029976369379
H	-1.60592478729801	1.41688359667696	1.51729289259192
H	-0.37769549557314	0.17035738087227	1.78057759355116
H	1.23967680967152	3.56299550797891	-0.15557712522777
H	0.14615295097054	2.25211602980853	0.27654780371272
H	0.03656660166133	3.00806077565193	-1.32111385590735

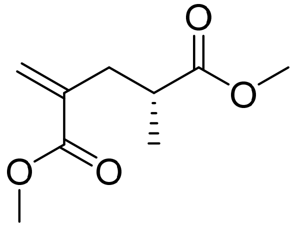

27

C	1.99535885381248	0.48389519241862	0.09387584741511
O	2.26702468421463	-0.72305994925074	-0.42885650393849
C	0.84453352285003	0.43176129362352	1.05103096192629
C	0.09380166932858	-0.85806561977512	1.24217973926171
C	-0.84068284037616	-1.19851650003885	0.07331245616372
C	-1.87796875675154	-0.10467404911937	-0.06500746850779
O	-1.88766186885701	0.43608139271997	-1.28875884251116
O	2.62080888812509	1.47187801828629	-0.18802418500300
O	-2.61759682908595	0.24792014175510	0.81689268375094
C	-2.82240800076520	1.49881271927010	-1.48129406376952
C	3.34123486038733	-0.75488740516931	-1.36719511239191
C	0.52457394521586	1.55877856904760	1.67495818769970
C	-1.53936554002729	-2.53560913743316	0.30532905891311
H	0.79591996945289	-1.68458371683540	1.36633428587445
H	-0.50554878998535	-0.78723221831098	2.15202193911508
H	-0.26489414811980	-1.23954264001404	-0.85230724897801
H	-2.59035942644533	2.32905481556022	-0.81419784399689
H	-2.71696536801607	1.80271231166966	-2.51925295028067
H	-3.83723168607494	1.15615391125264	-1.28115950074433
H	4.26063975536037	-0.39352703161198	-0.90742735393177
H	3.44416467482441	-1.79492647285390	-1.66557551955032
H	3.10881118150057	-0.12993559604170	-2.22950178016272
H	1.08584921888761	2.46596549929032	1.48901898433240
H	-0.30222407119155	1.59309328334811	2.37454433349453
H	-2.14772604316694	-2.49425005077045	1.21052428254768
H	-0.79960697744458	-3.32951913615651	0.42020986140656
H	-2.18899987765215	-2.79587762486063	-0.53198424813471

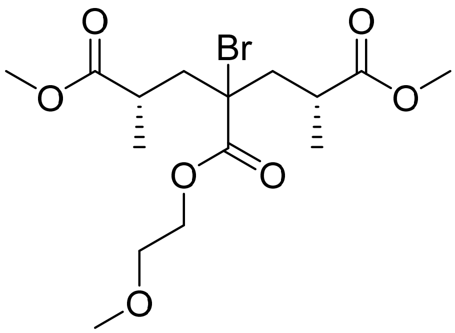

48

Br	-0.87818628374294	-0.89719196965649	1.82869872965200
C	0.81684101480322	0.54194719483755	0.09520435855868
O	3.16495372782456	4.19218852823696	-0.30952356284684
C	4.28304527569856	3.75459119594563	0.42734392294463
O	3.47525473937003	-2.10286767649055	0.14114140709317
C	2.49398916979954	-1.76228263592539	-0.69301176211364
C	-4.14254614841731	0.19208176109247	-0.39627767868861
O	-5.07044943585335	-0.32724454834793	0.16331703078204
C	2.02924585707156	2.20569608326840	-1.04953291212995
O	0.83317439177787	1.42575778181686	-0.90051632059683
O	-4.14806384013834	1.47400594346981	-0.79027318770882
C	-5.33801627540065	2.20496129871229	-0.49359151523742
O	1.68301548729905	0.44116387515548	0.92060292368519
O	2.64989257990701	-1.03147348225850	-1.64004173363214
C	4.73215275416753	-1.46953362265767	-0.09302598843857
C	2.01494576710525	3.40627149495123	-0.11477291734284
C	-0.40582700535286	-0.38433205481836	-0.00343342093953
C	-0.01816525643086	-1.64062380890986	-0.80150864718633
C	-1.61059058833253	0.33427791916282	-0.62675568499983
C	-2.87308001048171	-0.52908732071895	-0.79815849178407
C	-3.06356374072547	-1.02101103728798	-2.23849104732212
C	1.18543193649173	-3.83366049656467	-0.97728054415247
C	1.19109308991352	-2.44445145368813	-0.33075099568964
H	4.58169178614931	2.73362172732404	0.16112333234812
H	5.10449831685385	4.43098454783062	0.19638967822355
H	4.08122810231902	3.78792001642375	1.50456136575923
H	2.89200553077138	1.56337978671599	-0.87413180106634
H	2.02944573088050	2.53475539230242	-2.08687138212673
H	-6.20130030316639	1.73350414535318	-0.96264255280272
H	-5.49992910073847	2.24629158845354	0.58333657424610
H	-5.18152158742584	3.20281409002719	-0.89500083717657
H	4.63414385581811	-0.39247806618500	0.05000100107796
H	5.08147087960831	-1.66872245842671	-1.10567648776520
H	5.41722494007308	-1.88637076358994	0.64013273908273
H	1.15152766828128	4.03485962317426	-0.34274993402534
H	1.93540213483280	3.07146228401859	0.92625384209591
H	0.15533115676304	-1.32692418043096	-1.83602530314433
H	-0.88509039887685	-2.30342771624909	-0.79987428762496
H	-1.83048585480953	1.20905091868862	-0.01486437663601
H	-1.29689224424949	0.71763848335604	-1.59887006912409

H	-2.83525148147024	-1.39228149459437	-0.13229651030807
H	-2.20671028932843	-1.61102578064199	-2.56464777848841
H	-3.95629849620440	-1.64310777104926	-2.31808718640277
H	-3.16869326134342	-0.17444820854310	-2.92014495202761
H	2.06045923612399	-4.40994154950062	-0.67551428723017
H	0.29417083227404	-4.38534503595404	-0.67535650720855
H	1.18550168711979	-3.75211389532238	-2.06680787720418
H	1.17094095339115	-2.55930265250576	0.75418963562240

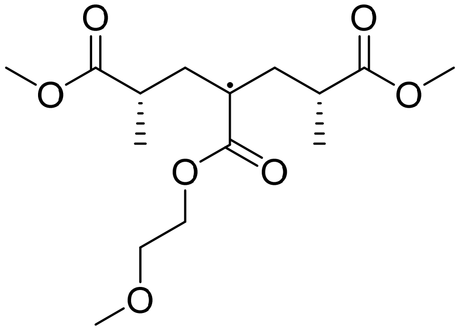

47

C	0.71446978386630	1.49205915509294	0.16249548744729
O	4.97587885336675	1.96204880711689	-0.58938008119747
C	6.06945674574526	1.87593641988945	0.28751406888160
O	-2.93044249285411	1.08064099345833	-1.41599294237581
C	-3.33851546583381	1.08844879538201	-0.14094166336388
C	-0.07075267298983	-3.06386541518514	-0.17535022109945
O	-1.04262331640722	-3.29429018247935	-0.84610025536858
C	2.71887122855388	1.55038060557300	-1.09548218213985
O	1.54094597391482	0.84957950746931	-0.69476185022500
O	0.88716761130992	-3.97081888526205	0.05832441333823
C	0.66879447079300	-5.25885601207234	-0.51831857915400
O	0.93212755836706	2.61359866227228	0.57199501972453
O	-4.18403583088721	0.34616714033334	0.28607933919338
C	-3.50601168201965	0.06451249215588	-2.24218561020327
C	3.81796710648327	1.36543810570360	-0.06924064285822
C	-0.44604763922255	0.69726342229923	0.52792869925890
C	-1.43801815846142	1.32850853018271	1.44125750936330
C	-0.68008736382115	-0.65756945289839	-0.05260155262918
C	0.22502983295072	-1.75361231097331	0.52434065696351
C	0.04733048364621	-1.92619370487104	2.03406707352062
C	-3.48999415901093	2.77672332670287	1.69037518499393
C	-2.56732490970931	2.08451644959268	0.69799844865376
H	6.32309182360012	0.83039904755324	0.50585693521499
H	6.91836557582862	2.35410170106009	-0.19851104323421
H	5.86195143127515	2.38992420553989	1.23443095905092
H	3.01798352425237	1.11278026278132	-2.04679712070451
H	2.50054080071731	2.60927336609430	-1.22987219602220
H	1.53426807680974	-5.85697107325574	-0.24579183445535
H	0.58175366571279	-5.18175231196469	-1.60174680739494
H	-0.24388167373605	-5.70263263866262	-0.12079378987298
H	-4.59290232236014	0.13451758353003	-2.23308535723332
H	-3.20696313374693	-0.92339525774611	-1.88750926975725

H	-3.11690070819495	0.24143121679220	-3.24138974728062
H	3.52659507140436	1.83372531923972	0.88044991316764
H	3.98015181550647	0.29218760995843	0.11170427546316
H	-0.93152173756786	2.05213268710863	2.08169383731769
H	-1.90404876359942	0.56418003281034	2.07110043398246
H	-0.53163839728025	-0.62188824921156	-1.13485094483985
H	-1.71694290669579	-0.95547858591602	0.11993590251601
H	1.26965509401269	-1.50881554152513	0.31790489233715
H	0.69441835426139	-2.71533749758446	2.41615736350710
H	0.29846154789626	-0.99965618389163	2.55359599097984
H	-0.98833722032876	-2.17909694534530	2.27495847949826
H	-2.92257981380807	3.48008969357773	2.30135660347274
H	-4.27874971422763	3.32806512884740	1.17636271611282
H	-3.96439304529700	2.04441548183929	2.34471020846711
H	-2.10575330221445	2.81420349888777	0.03020627898295

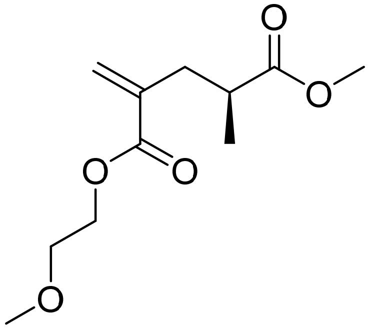

34

C	0.20213747349001	-1.53913464817043	0.04025283872358
C	-1.26929689663636	-1.34963008325984	-0.19707433624171
C	4.98320745365941	0.86232463349012	1.15267745653231
O	3.70378600520820	1.34789621801028	0.83494824498642
C	-5.01432368448879	1.68497339746323	-0.21787348654807
O	-3.64822101709986	1.42635600135941	0.10133900592907
C	3.32476447143005	0.00551333649953	-1.18968338040729
O	2.40454616505584	-0.84779676420298	-0.50638450030030
C	-3.24944124876210	0.15553822258499	-0.05757120474895
O	-3.97965416664381	-0.71412062637136	-0.45445909676380
C	1.10479584703235	-0.66081018847986	-0.76610283941065
O	0.70168366715335	0.13708112698898	-1.57635694638277
C	3.41751051546373	1.37521608600068	-0.53984977938879
C	0.70228377994251	-2.41827844701818	0.89989957882403
C	-1.80280846315077	-0.02137824057687	0.34782827121636
C	-1.68937460550113	0.08081531931007	1.87028109254237
H	-1.47005718388521	-1.39356055536416	-1.26900627913139
H	-1.81759731299433	-2.17259562756484	0.26494494586088
H	5.14219807615267	1.04535557582234	2.21426103867860
H	5.07659614672129	-0.21420411737073	0.96822608787040
H	5.76160962649278	1.38659330799241	0.58191725246248
H	-5.21180532988993	1.45403721197985	-1.26461387465584
H	-5.16982329854534	2.74332922380052	-0.02561028944025
H	-5.67284981405558	1.08103354898969	0.40650343359683
H	4.27540383868721	-0.52568914570792	-1.15177062544428

H	3.02270309030163	0.12391369901232	-2.23158425481250
H	4.18069126131812	1.95181898749589	-1.08495530338708
H	2.46281368357364	1.89109459042889	-0.64141645796144
H	0.04707957362884	-3.05647925172811	1.48173371906604
H	1.76882225606880	-2.52482224568481	1.04472778688624
H	-1.24770123518790	0.80324175170184	-0.10497497245927
H	-0.64730116039516	-0.00265331858469	2.18052824422157
H	-2.07555367841664	1.03516382142033	2.22761625649171
H	-2.25115683572750	-0.72300080026659	2.35376837359547

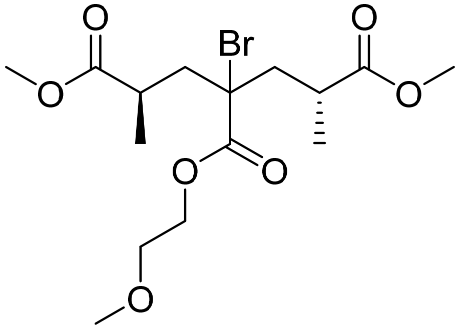

R,*R* Diastereomer

48

C	0.541554	-1.204956	-1.407277
C	0.073762	0.152378	-0.830298
C	3.163049	1.021927	-0.127410
C	-0.561471	-2.949537	0.129680
C	-1.395237	0.426002	-1.206425
O	-2.271776	-0.050762	-0.285225
C	0.933738	1.351579	-1.348780
C	2.461654	1.164678	-1.463493
O	0.643378	-3.274460	0.670421
O	3.072415	2.162638	0.607502
Br	0.231034	0.153220	1.145749
C	-3.667530	0.238436	-0.482292
C	-3.803305	3.549078	1.159678
O	-3.467485	2.539707	0.217649
O	3.732642	-0.002961	0.225120
O	-1.644650	-3.090463	0.682588
C	0.568635	-3.779201	2.003971
C	3.710864	2.086129	1.882680
O	-1.726598	0.935215	-2.272513
C	-4.097069	1.293948	0.532488
C	3.091474	2.327388	-2.238662
C	-0.405133	-2.421053	-1.284524
C	0.080556	-3.569584	-2.175385
H	1.516655	-1.472077	-0.980566
H	0.711913	-1.052559	-2.482752
H	0.714063	2.247495	-0.751024
H	0.588407	1.597899	-2.362684
H	2.680735	0.257676	-2.038754
H	-3.881645	0.568105	-1.504185
H	-4.214751	-0.692667	-0.304655

H	-4.883651	3.722589	1.166809
H	-3.302388	4.475152	0.865285
H	-3.459416	3.268866	2.159963
H	0.101188	-3.046914	2.669655
H	1.588107	-3.962943	2.354025
H	0.019377	-4.725583	2.022105
H	3.556277	3.039036	2.396205
H	3.268290	1.290320	2.489776
H	4.787008	1.928173	1.761245
H	-3.795082	0.987572	1.540612
H	-5.184500	1.420482	0.497392
H	2.719089	2.353189	-3.268215
H	2.863522	3.293010	-1.774071
H	4.181769	2.226651	-2.276825
H	-1.404340	-2.148260	-1.642207
H	0.070583	-3.273971	-3.229755
H	-0.564026	-4.448755	-2.065782
H	1.101373	-3.873758	-1.918948

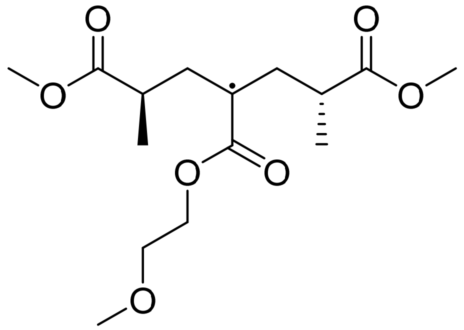

47

C	-1.805795	-0.774474	0.855941
C	-0.373776	-0.346241	0.642709
C	1.151895	3.180895	0.150634
C	-4.162068	-0.766039	0.044690
C	0.658023	-1.402539	0.657809
O	1.892217	-0.856555	0.745668
C	-0.019954	1.103298	0.855386
C	0.734481	1.792581	-0.298532
O	-4.981642	0.062891	-0.660842
O	2.354223	3.511391	-0.396884
C	2.983117	-1.786851	0.839414
C	5.635716	-2.427092	-0.794176
O	4.503042	-3.207606	-0.432733
O	0.488703	3.903861	0.881525
O	-4.535861	-1.589423	0.868574
C	-6.365948	-0.082197	-0.344705
C	2.824997	4.808856	-0.026835
O	0.406147	-2.595105	0.615928
C	3.280309	-2.469967	-0.495266
C	-0.092847	1.923282	-1.576388
C	-2.716011	-0.535476	-0.357972
C	-2.383473	-1.450067	-1.533115

H	-2.199624	-0.229070	1.725703
H	-1.857632	-1.831863	1.146228
H	-0.940680	1.666374	1.063456
H	0.574256	1.181783	1.779423
H	1.648158	1.231026	-0.525816
H	3.852718	-1.213251	1.174561
H	2.794547	-2.526800	1.629472
H	5.700730	-1.510782	-0.199656
H	6.531924	-3.024334	-0.609955
H	5.594409	-2.175685	-1.858303
H	-6.716956	-1.089171	-0.600372
H	-6.542400	0.137730	0.713371
H	-6.925109	0.644559	-0.941477
H	2.939236	4.874030	1.062061
H	2.140742	5.581181	-0.392911
H	3.805572	4.961755	-0.491138
H	3.290577	-1.747195	-1.326296
H	2.484832	-3.189357	-0.719379
H	-0.313201	0.942970	-2.008172
H	-1.040592	2.442570	-1.390383
H	0.454310	2.504100	-2.330247
H	-2.619472	0.512106	-0.668455
H	-2.439476	-2.505868	-1.239626
H	-3.092466	-1.301717	-2.358460
H	-1.376378	-1.255844	-1.917436

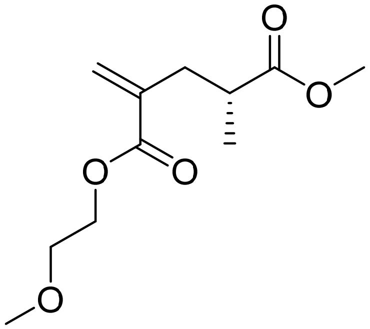

34

C	3.554994	-1.085643	1.998023
O	2.312675	-1.119543	1.295130
C	0.799991	0.428719	-1.504186
C	1.046951	-0.846812	-0.682759
O	-3.077857	-1.420759	0.483870
C	-3.843374	-2.536545	0.049793
C	2.410332	-0.787040	-0.021703
C	-0.641271	1.870972	0.112391
O	-1.547321	0.927667	-0.237206
O	-0.820459	2.760283	0.930530
C	0.624171	1.662755	-0.650955
C	-2.772905	0.972199	0.511005
O	3.450663	-0.499097	-0.599593
C	-3.659352	-0.183805	0.057349
C	1.570241	2.612845	-0.556889

C	0.991884	-2.090288	-1.565796
H	3.989348	-0.081579	1.963432
H	4.248328	-1.823731	1.583048
H	3.360226	-1.342945	3.042816
H	1.630748	0.582132	-2.206866
H	-0.086570	0.301266	-2.138683
H	0.279315	-0.943456	0.094584
H	-3.876017	-2.575652	-1.043105
H	-3.362938	-3.447297	0.417198
H	-4.858077	-2.486939	0.456033
H	-2.546051	0.875567	1.578987
H	-3.278225	1.924001	0.314068
H	-4.654379	-0.083914	0.504117
H	-3.743805	-0.186071	-1.035385
H	1.432405	3.500350	0.055708
H	2.515373	2.524374	-1.084569
H	0.003360	-2.198514	-2.023794
H	1.194388	-2.993060	-0.979138
H	1.736965	-2.045167	-2.367794

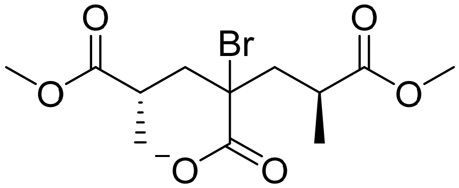

37

C	-3.63825106428605	0.91298248499186	0.26836346159299
C	-2.15319372664407	0.92909548934297	-0.00687709976548
O	3.94257408511553	0.43267916140352	-0.35484829039268
C	2.91755740793432	0.90510114086909	0.38189427703136
O	-0.72441912497919	-3.00311750344515	-0.05456400871773
C	0.01834491476352	-2.31435957849666	0.65398104784610
O	-4.30033885877876	0.13239630914605	-0.60480729271320
C	-5.69108128608948	-0.01842021408058	-0.35973783173822
O	-4.20094005429809	1.49265355904585	1.16354850490274
O	2.61503152800521	2.07246517470289	0.37674046448797
C	4.56004060289285	1.37954874398876	-1.21197204770024
O	0.82526624208343	-2.65354537923171	1.53918913358824
C	-1.59360643539434	-0.38005375703895	0.58545502683034
C	-1.54159298432112	2.22485530362026	0.50758545050970
C	0.79037973599535	0.03608477978304	1.40335438710499
C	2.29427492339294	-0.17977813527236	1.22673419952775
C	3.00612177228220	-0.20563127709282	2.58162567451910
C	-0.11035589257447	-0.73211763039288	0.44152619775727
Br	0.45657361662964	-0.35915419286814	-1.42940568508353
H	-2.03080390457426	0.87186287943303	-1.09067359670346
H	-6.05681085484654	-0.69673683078871	-1.12727177836887
H	-5.86361674924794	-0.43941484572610	0.63141676396359
H	-6.20183304844004	0.94373154266531	-0.42450335731018
H	4.96453590068012	2.21935999742702	-0.64472977882894
H	3.83757815678879	1.76172704389682	-1.93492424576193

H	5.35933081826901	0.84604209421578	-1.72255570436337
H	-2.13503343346890	-1.21428926802230	0.13655173454805
H	-1.80364981844359	-0.39637579338542	1.66315226414600
H	-1.54045255297152	2.25556889966183	1.59844683774106
H	-2.11598781768831	3.08428219415374	0.15493539944615
H	-0.51740399558576	2.33238924864935	0.15094641989190
H	0.51043235350479	-0.33191557278948	2.39270172583235
H	0.58783154805397	1.10599701647266	1.37802182582555
H	2.46834374381567	-1.13821696230683	0.74287125240534
H	4.08690335812924	-0.31696042084370	2.46522123811923
H	2.81042544567180	0.71258959650354	3.14273016644980
H	2.62654344862408	-1.05821329819161	3.14563926338028

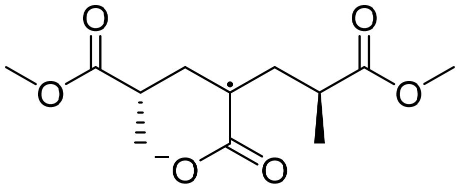

36

C	3.36336958156518	0.69115724736752	0.17198323595059
C	2.07392602773359	0.03321327128847	-0.24847665491744
O	-3.87888563260025	1.00296901060289	0.81216571216816
C	-3.11213894085234	1.25228198470506	-0.27016355922951
O	0.49732280949176	-3.16423550807919	0.12343252619477
C	-0.45598279615774	-2.37715932998596	0.32915685226708
O	3.78326226655476	1.59257967562641	-0.74828563044329
C	5.02812957541710	2.21227113552279	-0.46448520212559
O	3.99915666218399	0.46605460381874	1.16862120233312
O	-3.29162110687881	2.23099826761888	-0.95483788735361
C	-4.89290423667848	1.95916009378421	1.07408106252308
O	-1.68647286484958	-2.62765131780892	0.29956284526347
C	1.34135860752283	-0.59751633665066	0.94763359529141
C	2.38708903347791	-1.00111344854218	-1.33509063504486
C	-1.12203862991377	0.11494118522978	0.73663702261651
C	-2.06838735599744	0.19017246289842	-0.48147728347404
C	-1.32546690195466	0.41947343936006	-1.78665155323445
C	-0.07664211082693	-0.95245307352844	0.63938029808195
H	1.45065403905739	0.81881760990076	-0.68856552625609
H	5.81898254612047	1.46720830357641	-0.36612883025038
H	5.23296717435080	2.87463973284708	-1.30325424902860
H	4.97517693632306	2.78259838461524	0.46424117054782
H	-4.46402408785655	2.95084788152765	1.22683183886590
H	-5.59891906237787	2.01350257970024	0.24361739518434
H	-5.39707577882025	1.62042621097966	1.97685584487875
H	1.87440145867039	-1.50228785634250	1.24237659453938
H	1.37926809303085	0.11034517329354	1.78523059058678
H	3.12004365651230	-1.72275541304866	-0.96769808080445
H	1.49023107658743	-1.57470722946659	-1.56619099368467
H	2.77435087471629	-0.52286120664438	-2.23723511967545
H	-1.74237666073134	-0.07271526013717	1.62088220841390
H	-0.64382279343860	1.09450059453188	0.87738432623818

H	-2.57024704623389	-0.78268932298277	-0.50141246006924
H	-0.63522682143224	-0.40706011211022	-1.96104236496451
H	-0.75691255976846	1.35331873889055	-1.76026916115410
H	-2.01503203194692	0.47920182764136	-2.62984413023487

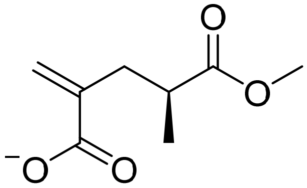

23

C	2.10549295727810	2.08904933031795	-0.68720312187082
O	1.44053350363401	0.86433438220305	-0.95535712049682
C	-2.04350713824456	0.42729544969622	-0.47491912089559
O	-2.55090133034191	1.55762309661288	-0.56746522835565
C	1.67536230308834	-0.12803757082619	-0.08783305272309
C	0.71166319500064	-1.27335955457114	-0.26843315214651
C	-1.11867744115869	0.18185801562324	0.74648358651244
C	-0.38853452500695	-1.13189167165969	0.80164992829776
O	-2.17507126273978	-0.56276542351080	-1.22980085417087
O	2.53585182536649	-0.06404216201725	0.75816268243457
C	-0.98012176913126	1.09756536170407	1.69779897440459
C	1.42676058971729	-2.61097761845171	-0.14374631493694
H	1.78970841774638	2.77613449697847	-1.46863022168673
H	1.80188153251277	2.46878288630574	0.28958221179081
H	3.18895598150628	1.95808853553911	-0.70095583490352
H	0.22265206065609	-1.17173495987458	-1.23804112571891
H	0.06731391871179	-1.27337612034645	1.78828660561463
H	-1.10480592101889	-1.93581649651923	0.61897537802410
H	-0.34053342135229	0.93482014564260	2.56222831955186
H	-1.52028872491659	2.03326406133160	1.60940861124120
H	2.17868981177644	-2.74309246822444	-0.92581609287213
H	0.70306871540749	-3.42414591474780	-0.22771194130939
H	1.92882372150881	-2.68748880120567	0.82300688421501

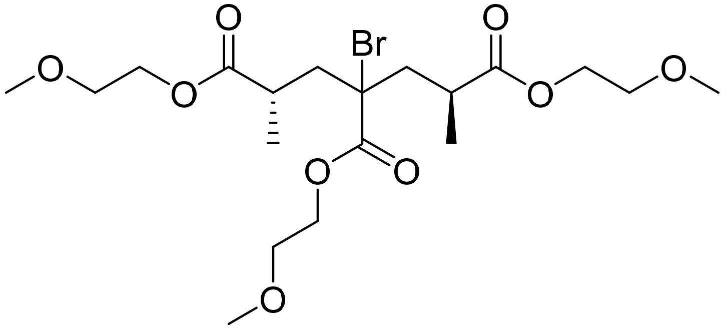

62

C	-2.06933063529452	2.98677972293935	0.70195195858083
O	-2.31494073656881	3.12577130421780	-0.61728052752154
C	4.87039892193352	-1.69475789007650	-0.76458139963605
O	3.69512979118782	-0.88819241709592	-0.64016310686322
C	-2.16826856186927	4.42556433991000	-1.18855303760647
C	-0.95100172173310	4.42843317130912	-2.07985117788543

O	0.18174852589519	4.16857973745565	-1.29342449180590
C	1.37503438844158	4.18155076350796	-2.03511639416507
O	-2.43676149122213	-1.75027091620284	-0.16363084909402
C	-1.60177181224765	-1.57602497200858	0.87105449020213
O	-5.52553120230379	-3.39311319555926	-0.90701201562514
C	-6.38751984681796	-3.49452196560893	-2.01195195756859
C	-1.97519588482526	1.52543368033862	1.09185236413394
C	1.46608605361789	-0.32169336154921	-1.19833516893400
C	2.67263579044325	-1.20313906672023	-1.44211763611816
C	5.86906418116798	-1.14531838000554	0.22754455674516
O	7.02843962533262	-1.92847513214346	0.13207076851624
O	-1.72270346388832	-2.14334626146894	1.91675694201878
C	-0.50982033367168	-0.56765095576421	0.48809634408036
C	0.32144819583766	-1.19675747813432	-0.64585496208324
Br	0.65035656885484	-0.32269098532498	2.04015504134077
C	-3.55396940233078	-2.61107909551975	0.08253758950844
C	8.05111147692674	-1.48199241343507	0.98551672668182
O	2.71353528837386	-2.08996382449610	-2.25349541340270
O	-1.96591786068119	3.90466202048553	1.46576893009034
C	-4.38932460514617	-2.61846303856373	-1.17643558301101
C	-1.10543910393324	0.78151102862579	0.07008489924264
C	-3.39188942299616	0.96824391396500	1.26269224355971
C	1.09821329136776	0.37101343672477	-2.50976863423708
H	5.25506553552504	-1.64030859533497	-1.78391725650479
H	4.62964630743471	-2.73603109852532	-0.54524067548823
H	-2.07041056408458	5.16356799601748	-0.39332461872738
H	-3.06531459337705	4.63083561450553	-1.77429065062424
H	-1.05366159455757	3.66358309761060	-2.86437996795242
H	-0.87075225933242	5.40896939745032	-2.57209080569752
H	1.55854126783152	5.16958206146237	-2.47568005840991
H	2.18753552968092	3.93993361383055	-1.35160466662799
H	1.35010780731457	3.43812726919238	-2.84289693790593
H	-6.74619074590649	-2.50704823725750	-2.32918999242752
H	-5.88848066444871	-3.97541879354870	-2.86271024657668
H	-7.23849999779482	-4.10190288482611	-1.70856105512502
H	-1.47888925051737	1.52956724255175	2.06332238366033
H	1.75264921585017	0.41899181087409	-0.45107076667169
H	5.44935238413976	-1.18323407273307	1.24256389169530
H	6.08701634009111	-0.09284223557565	-0.00336001636711
H	-0.36484094566954	-1.44358498614776	-1.45894642264029
H	0.71482582194684	-2.14574007976928	-0.27532607193949
H	-3.20437209508056	-3.61255666310834	0.33519302990866
H	-4.13233723696974	-2.22864968927599	0.92572462721386
H	8.90223323783349	-2.14778084622263	0.85358182748051
H	8.35452506670639	-0.45668880396383	0.73798418359304
H	7.73398735809180	-1.50796345033131	2.03577053585283
H	-3.81542667921065	-3.04038208060765	-2.01369499637536
H	-4.66904278014221	-1.58934117562455	-1.44589915698061
H	-0.27244627709638	1.43188668363512	-0.20365600325374
H	-1.68562506350087	0.61322769427324	-0.84041888056163
H	-3.98224806514353	1.63239430364133	1.89593862431439

H	-3.37881261420173	-0.00656807281953	1.75040563755097
H	-3.89516316653740	0.87368657099086	0.29796983050874
H	0.89740062749721	-0.37366169736207	-3.28143527638627
H	1.91562855980308	1.00660782918812	-2.85660497143122
H	0.21218451997436	0.99584450800849	-2.38568757624709

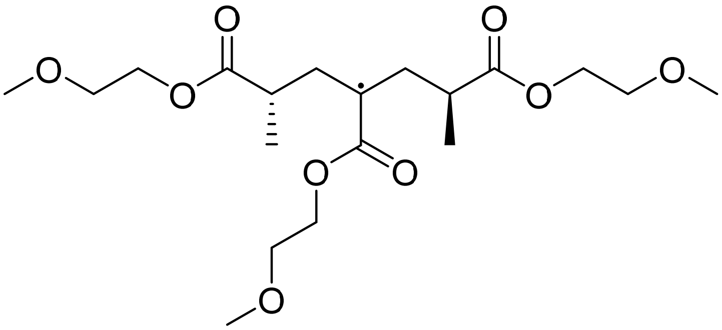

61

C	-0.32648833489005	1.98712027775805	0.05760308475996
O	-1.36433928418020	1.22548935261881	-0.33674722465852
C	-2.51923484539640	-2.53347674869702	0.42571670931853
O	-1.40368005298274	-2.25829084269626	-0.42750796494164
C	-2.00986287845401	1.58663423291352	-1.55957802415841
C	-3.10539282470457	2.59704321208599	-1.33923576474079
O	-4.15540626386340	1.99077865699082	-0.62683909108446
C	-5.21252057637943	2.87938829655061	-0.36989349404617
O	3.75098440420143	-0.85350409363197	-0.28985027065370
C	2.93460937110512	-0.35773624032121	0.66569010433769
O	5.03921876573644	1.72849827775434	-0.62116970939235
C	5.52539439909272	2.29532188159477	0.57531380950887
C	0.26530458610571	1.48807953139055	1.35816508441959
C	0.85567288952433	-2.46899143883433	-1.09203699002104
C	-0.25487156073666	-2.89726546088248	-0.15439419655507
C	-3.50578395515234	-1.40638908684400	0.22104930092533
O	-4.64341069473333	-1.70025917023092	0.98825147281241
O	3.33485291842582	0.10595032683626	1.71347695107254
C	1.52659789867538	-0.45584285648163	0.31173108731168
C	1.09811118535299	-0.94605562788446	-1.03086308042139
C	5.15508603595217	-0.66632579717941	-0.11774192158193
C	-5.58634155037115	-0.65862276406860	0.96268607119737
O	-0.14117762631074	-3.70636935683604	0.72627168435034
O	0.04698663256978	2.95515370505187	-0.54886440398007
C	5.62915103121140	0.50375574328165	-0.96081464528987
C	0.54190521812768	-0.02817785283648	1.34771372143113
C	-0.67697558053533	1.84951062068673	2.50662269440339
C	0.53794266247956	-2.90169335299262	-2.52166448344180
H	-2.18934316240006	-2.58639008427123	1.46384017881485
H	-2.95785503122388	-3.49640672470517	0.15715170411473
H	-2.42944755705953	0.65788455492242	-1.94815133093420
H	-1.27195400350258	1.98498815967995	-2.25666684470446
H	-3.46136043001147	2.95018841566637	-2.31843641515778
H	-2.71081144885006	3.46465487803514	-0.79374256505676

H	-5.98650241819418	2.32608422311690	0.16129231360218
H	-5.63465859027593	3.27624584732742	-1.30205334644983
H	-4.88473416785236	3.72222080846077	0.25136329155285
H	5.07294684788378	3.28159087288529	0.66472360089012
H	6.61713981422483	2.40573447232268	0.53779737493634
H	5.24614306117961	1.70088733660813	1.45090222062061
H	1.21038113069182	2.01929743319779	1.47891370629090
H	1.74986821236348	-2.98666342506055	-0.74160116201200
H	-3.05933765602003	-0.45135913776808	0.52799102176225
H	-3.76652070325190	-1.31906816114281	-0.84446586447314
H	0.17272048236771	-0.44668205880965	-1.32644358265947
H	1.86499364587047	-0.70957903748986	-1.76938990730489
H	5.37718243888877	-0.52860207327982	0.94002231743157
H	5.63206211564190	-1.58254150924019	-0.46951003919579
H	-5.16612942514674	0.26412647628172	1.38221809292582
H	-5.91633524758597	-0.44648464687981	-0.06279468027295
H	-6.44028480303378	-0.97548564024879	1.55971852815542
H	5.36765668438676	0.31934487124748	-2.00500075032184
H	6.72590831506686	0.56224288429369	-0.88680650650217
H	0.93150107632384	-0.28580158213299	2.33436002730262
H	-0.40696283781316	-0.54952122503647	1.20575008261306
H	-0.86668671059226	2.92378024214335	2.53700860066197
H	-0.23207366587646	1.55464427037970	3.45781828385099
H	-1.63353021392249	1.33322024152655	2.39930206810189
H	-0.34698796153515	-2.38387198462288	-2.89551273623998
H	1.37721931516482	-2.66578540316738	-3.17772530119210
H	0.35531492422320	-3.97614071933627	-2.57756389203245

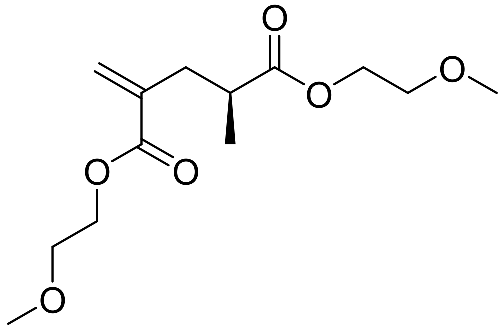

41

C	-1.44910527509710	0.50596453700545	-0.29668552781031
C	-2.50982729454167	-0.57453216814013	-0.34219905254274
C	-5.56707526323068	-1.04337736326366	0.19924308254068
O	-6.43926755233996	0.05599605004886	0.27980100107308
C	1.09848477550223	0.77289461773987	-0.18261634017283
C	1.11078333002848	1.92472252771669	-0.84223633525712
C	2.37056020470214	0.21157373890894	0.37831098471676
C	-0.10047027331803	-0.10213822381091	0.06577489320773
O	-3.60603737297080	-0.15333215782881	-0.99436698390663
O	3.45508840150312	0.94443180290932	0.08324115798171
C	4.70558729670219	0.47078139249019	0.59460596222865
C	-5.82849596620794	1.27076165477821	0.64459374654923
C	-4.70754192657061	-1.07351161462360	-1.05021877557923

C	7.60592965757565	-0.12705605245023	-0.31481210863595
O	6.57199883518222	-0.98516224181530	0.10221071615040
O	-2.41379489940172	-1.66459123669733	0.15559850168238
C	5.29253461645160	-0.58996781603001	-0.32698489260605
O	2.41267205066897	-0.79911749677173	1.03050373801298
C	-1.89519681384417	1.58285473906321	0.69861292531524
H	-1.41558291055828	0.94341729029032	-1.29846655593801
H	-4.92723086784021	-1.10024696060638	1.08988842483388
H	-6.20308956500876	-1.93120646482518	0.18680061597639
H	0.20835263500754	2.35683369545026	-1.25670570470912
H	2.03240708702709	2.47074196003899	-0.98803942183734
H	-0.10361633379526	-0.40588537611067	1.11500917537883
H	0.04357553104536	-1.03749573169766	-0.48160505317331
H	4.57087320379624	0.06357445663870	1.59634258010657
H	5.34380331857992	1.35271617076065	0.63877980348676
H	-5.20190911791338	1.67405858742653	-0.15795465575346
H	-5.20835675921635	1.14630001504384	1.54247011315559
H	-6.62706679903430	1.97903482050857	0.86228521330075
H	-5.29490499799614	-0.76451293071098	-1.91436615003602
H	-4.31925741775545	-2.08081143802804	-1.20464253085029
H	8.54169583563475	-0.55147618882170	0.04564221207415
H	7.50201797962050	0.88374008645514	0.09732888955122
H	7.64298858917631	-0.05660990538831	-1.40907714888724
H	5.32368532406308	-0.20685989963216	-1.35610563013053
H	4.65882112611016	-1.47745614039797	-0.30634269732324
H	-1.13979792166755	2.36404139875962	0.78522974356411
H	-2.03638702128424	1.14466239407461	1.68962069277824
H	-2.83308044878491	2.04317947154276	0.38735539148408

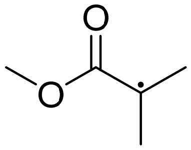

16

C	1.11149347996953	0.16626502696323	-0.02040456546796
C	-0.25966822725508	-0.30919135130581	-0.03130868767639
O	-1.17203981974767	0.68508760761203	0.04133418780956
C	-2.53055672730630	0.26071869114635	0.02752531245076
O	-0.58149075505969	-1.47813835889709	-0.09938593428140
C	1.45820720037509	1.60972978661014	0.04129265441152
C	2.20394169396663	-0.83472980999423	-0.10642826556378
H	-2.74279915533308	-0.37978756281728	0.88396005744552
H	-3.12590229457766	1.16929268066279	0.07477678893060
H	-2.75128010933036	-0.29299428137069	-0.88534168016855
H	1.87446447445806	1.94213987900228	-0.91732202433679
H	2.23694124975142	1.78261781817794	0.79054140351053
H	0.59765105186664	2.23251341398712	0.27092904696418
H	1.80772698979350	-1.84138640420555	-0.21869057447161
H	2.86539684245574	-0.61007256479206	-0.94999596541533
H	2.82825910597323	-0.79925457077918	0.79337724585914

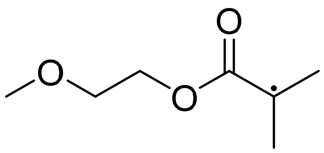

23

C	0.97578882919226	1.13432862443779	0.28143875418917
C	2.31418149374215	0.52043892698056	0.58571848277090
C	-1.25712262924269	0.48826384174021	-0.03712219041050
C	-2.21940509687034	-0.59768385815291	-0.01225732334736
O	-0.01227694177988	0.10741967764283	0.32190057826197
O	2.71936939207073	-0.27932826514573	-0.49355298848287
O	-1.52106686412671	1.63328649420912	-0.34492082793195
C	3.97606209335760	-0.86783771086607	-0.28561034786296
C	-1.84633130162997	-1.98740124496777	0.35727947416040
C	-3.61683593979465	-0.30830626086071	-0.41972529510599
H	0.73260948348045	1.90372730069413	1.01840426169707
H	0.98295542275080	1.59871327151112	-0.70602793381307
H	2.25604641702937	-0.07570495994938	1.50803794011007
H	3.03918273925144	1.33076987014514	0.75544559146687
H	4.75683653326735	-0.10648308333940	-0.15543602621162
H	4.20859198034258	-1.46711897156534	-1.16435764391974
H	3.97303053493338	-1.51655698216454	0.60017016497285
H	-2.58278820537946	-2.40918121982160	1.04768316898038
H	-0.85806859984242	-2.04448524775950	0.80629954420918
H	-1.85265201438832	-2.62927879127404	-0.53212975219053
H	-4.31477392512125	-0.57356285135143	0.38150057313568
H	-3.89661912839356	-0.91730202667594	-1.28676936247754
H	-3.74480627284890	0.74223746653348	-0.67057884220043

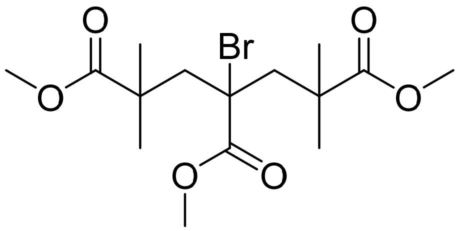

47

O	-0.15872543999320	-2.18565629225960	1.62013721459725
C	0.09488450512214	-1.04404542129115	1.36167893991681
C	-2.55831216282072	0.16017646847932	0.22798379454934
C	-3.43594321452009	1.32839718008311	-0.24653430440092
C	-1.12214290417240	0.54909321193173	-0.18997947999370
C	3.70810144190685	1.05292225938722	0.08121619320808
C	2.77803070271078	-0.10335302679812	-0.28852509430237
O	-4.73198470552841	1.12136368245493	0.02001172247363
C	1.33932213231728	0.48678739280472	-0.25718005740886
Br	0.02288495835745	-1.81305341098847	-1.37031858666411
C	0.09517016088981	-0.39551901699689	-0.02621513658253
O	0.37515351255579	-0.12171679585036	2.29063894457631
O	3.49646902462053	2.12695022084747	-0.69456594706493

C	4.32638181955535	3.25652719846005	-0.42917609530503
O	-3.04181247015748	2.32721577193546	-0.78934697989962
C	0.38453670436727	-0.58718481495492	3.64061592791497
C	-5.62062673320462	2.16031382316870	-0.38908598324996
O	4.54570100821678	1.02967380592338	0.94284271479802
C	3.01141572096315	-1.23476211657306	0.70883885419951
C	3.20854362829825	-0.56058058528309	-1.69316391160877
C	-3.08401228372269	-1.12593205873231	-0.42095532888832
C	-2.73292583384695	0.05658323038653	1.75258553158247
H	-0.87175891210418	1.45926920311656	0.36006907213468
H	-1.13981156194485	0.83872259739572	-1.24080935711082
H	1.17425510163684	1.03424348499298	-1.18614203229525
H	1.29746953292686	1.22978668100796	0.54179017943012
H	4.01002398699748	4.02698455729923	-1.12742937546052
H	4.19449793458293	3.59344640210563	0.59887780407626
H	5.37503947217275	3.00506390387640	-0.58728565291671
H	1.10203370933976	-1.39967999260439	3.75555983475367
H	0.67321080819361	0.26783174344909	4.24571541524628
H	-0.60567959090429	-0.94250572706785	3.92751816453670
H	-5.37042780706699	3.09587587613120	0.11076385153185
H	-6.61516928693696	1.82874790996191	-0.10253297662996
H	-5.56170893101717	2.30885503806299	-1.46705004225502
H	4.05537390860198	-1.54525274170043	0.67717127588357
H	2.39201302643105	-2.09768586273908	0.46149293153617
H	2.80238128933385	-0.92186611740435	1.73245722126857
H	4.28527402310235	-0.74708138805934	-1.70744369592588
H	2.98294949631655	0.19852246866608	-2.44272988285888
H	2.70455675752618	-1.48292913265133	-1.97123853691240
H	-2.52382192934777	-1.98864035550672	-0.06225372891294
H	-2.99455376191260	-1.09397531601902	-1.50816752750960
H	-4.13330002943800	-1.26617969866450	-0.16320527060460
H	-2.26176865753342	-0.84915585539406	2.13372038771385
H	-2.30296519788103	0.92060305191015	2.26418809679216
H	-3.79189995298976	-0.00244043629946	2.00143491204142

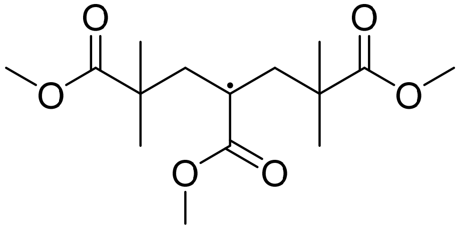

46

O	0.70426213253379	-2.91272665980652	-0.60724638599875
C	-0.10337450764571	-2.19855005740581	-0.05088018208187
C	2.29732849376284	0.13976722703298	0.41066267055786
C	3.42923734523074	0.84087958228748	-0.34005147282738
C	1.15780635019667	-0.08890836390360	-0.61540761745439
C	-3.44802546112959	1.09734499640792	0.01253425160807
C	-2.32030206874402	0.34630763285594	-0.69211606139898
O	4.53736746786840	0.94671527143071	0.40716471089253
C	-1.23578932323831	0.06511468011826	0.39559603814306

C	-0.06782348434974	-0.74046678698699	-0.06933341472941
O	-1.13554800373706	-2.69799588965746	0.65962592496740
O	-3.03639002884509	2.27664472419890	0.50465077955103
C	-4.03042403174996	3.03832046372267	1.18903375887000
O	3.35952385428112	1.27696847876243	-1.45869018001218
C	-1.23939435992088	-4.11861733126871	0.66472434535004
C	5.63735984676391	1.60841976588838	-0.21704941537213
O	-4.57733600872897	0.70405717677933	0.13398392656637
C	-2.89017322234948	-0.94091244676526	-1.27905271002128
C	-1.76616267979662	1.22646477835750	-1.81775254988380
C	1.84706895221957	1.06998197685417	1.54442190532354
C	2.80990331942043	-1.17584606059153	1.00182684641087
H	1.55610914653671	-0.70192253166014	-1.42619856153203
H	0.89795631484557	0.88314160454764	-1.03683096198854
H	-1.72307396195657	-0.45039911742545	1.22720524256984
H	-0.88171216973533	1.03112195289126	0.75979233592006
H	-3.53377482817553	3.94714972003806	1.51879312126029
H	-4.41615643269336	2.48270191010769	2.04368971080834
H	-4.85713309548989	3.27480479985324	0.51960192267248
H	-0.33986738667511	-4.56821769669521	1.08551521989042
H	-2.10556808641431	-4.35143072255823	1.27894000382743
H	-1.37817652594331	-4.49523551956989	-0.34926574476522
H	5.93870376305826	1.07666177248700	-1.11925782586460
H	5.36785013429825	2.63041774494540	-0.48319419245015
H	6.44075912056300	1.60344217333667	0.51488679312618
H	-3.29828955706545	-1.58808151396498	-0.50440290868552
H	-2.11083912581943	-1.48594630720258	-1.81566029000609
H	-3.69100846058300	-0.71150336023923	-1.98237499937052
H	-1.33262618619549	2.14978517911173	-1.43285032482309
H	-0.99931297439128	0.68695285060074	-2.37589322213307
H	-2.56106969769212	1.48882955624749	-2.51928724724726
H	1.47599807533302	2.02227453946584	1.15734531809227
H	1.04634216215272	0.59827811144450	2.11729912850306
H	2.67431482374941	1.27304902281693	2.22494712168049
H	3.65707782529980	-0.99227525740645	1.66146912019546
H	2.02166444627361	-1.65489116993098	1.58507682424937
H	3.11038109467780	-1.87506089955186	0.22069424760976

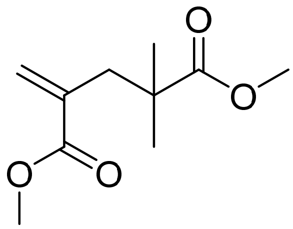

30

C	0.47391071013295	-0.89090907696020	1.52069676888083
C	2.00884612928539	-2.13333461332133	-0.01787488614711
C	1.99302571082838	0.30506535295232	-0.11845043793091
C	-1.02612690270433	0.10798521854706	-0.91684851062139
C	0.02691697820462	-0.96749406845653	-0.98369505748892
O	2.53481259593299	0.78233025809115	1.00842895084535

C	-4.34047543360154	0.49205127949484	0.64363812867854
O	-3.12201630486252	0.78810942351494	-0.03589705629593
C	-2.27329357572316	-0.23951809692171	-0.16698630048996
O	-2.50899995989116	-1.33772724488750	0.27181261047503
C	3.44977841490629	1.86495819572171	0.84423911719527
O	2.25047775919170	0.76192426552268	-1.20326808621767
C	1.09607377232183	-0.90582842297721	0.12465471477655
C	-0.90316833695957	1.29010467682332	-1.51069766003796
H	-0.09736985265774	0.02201226389593	1.69957953027039
H	1.24270924729061	-0.95548346698864	2.28973346656495
H	-0.20481007916705	-1.73838789505031	1.62553337730970
H	2.45231466254677	-2.17829839454008	-1.01415777469973
H	2.81288336069687	-2.10934180160300	0.72040920644605
H	1.42596153686981	-3.04228329029391	0.14257948114794
H	0.53334078617053	-0.90927181193644	-1.94709080253574
H	-0.46228711789943	-1.94025080169472	-0.91368281897091
H	-4.91953925661429	1.41173008264475	0.62682625515696
H	-4.13980103500072	0.18640215226351	1.67048469378792
H	-4.87673106522462	-0.30879065250592	0.13498187667001
H	4.30104342731620	1.55655793304695	0.23734566254800
H	2.95896790672621	2.70911577118723	0.36062878243234
H	3.77214170394979	2.13447929172770	1.84665152003620
H	-0.01098598825026	1.53016121110630	-2.07660451103533
H	-1.68409179381455	2.03543426159711	-1.44103524075047

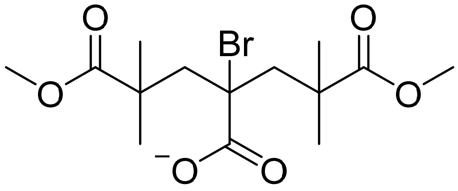

43

C	3.56344738485104	0.23088404960453	2.03964181617478
C	3.40053607961689	-1.01838310290485	-0.10881436840066
Br	0.38197937863367	-2.37513297772545	-0.55003831584496
C	0.09140437580952	0.50330374231266	-0.88953500834429
C	-3.53337534944894	-1.13987309874751	1.57228080346647
C	-2.77669239819219	-1.24795328293787	-0.79888280465307
O	-3.57031368898809	1.28975965784394	-0.67164684058351
C	-2.94797758367836	0.94657688984534	0.46502952606544
O	1.87057419270927	2.24082468943423	0.43924827739430
C	1.69274666195715	3.40009789914096	-0.36707507886451
O	-2.83288510776830	1.70562457603036	1.39542137235936
C	2.81374216087180	1.38475958118992	0.02929725827294
O	3.69178807133625	1.68880968267671	-0.73548387045202
O	0.99791488914592	0.43248149398461	-1.73002401053350
C	-3.69704199145065	2.68846623140885	-0.87650398826771
O	-0.80848683530833	1.35819867633969	-0.77911058017582
C	2.72879400289643	0.04336534872523	0.75770180376937
C	-1.14678117952100	-0.64436163433698	1.13002985447164
C	0.12096758530268	-0.56778190696620	0.25914579683284

C	-2.57180136084876	-0.53519253053837	0.53397246222087
C	1.29809823760082	-0.30702143662149	1.22498789662014
H	3.61563319520438	-0.71243822230326	2.58952295545616
H	4.58046802219602	0.53783030771278	1.78686289872790
H	3.12454614410578	0.98685803294201	2.69515270684525
H	3.35150494500296	-1.99095736886655	0.38655240270983
H	4.44638692489398	-0.75164379445597	-0.26874674423177
H	2.90773235233986	-1.08708721760879	-1.07615661708545
H	-3.34480947446667	-2.21165612277464	1.67135916584660
H	-3.39739960380307	-0.66555667880139	2.54578648226433
H	-4.57415531856273	-1.00212273222168	1.26625671399716
H	-2.15142952123986	-0.82308927255110	-1.58222917595974
H	-3.81836676121277	-1.15805726275323	-1.11258237434043
H	-2.53295363892199	-2.30612648384133	-0.69717700192777
H	1.43104580578925	3.08717318680622	-1.37851208914685
H	0.86235524632655	3.94187963483591	0.07942699288723
H	2.60022671415221	4.00664314483516	-0.37845843035448
H	-4.27744385577918	3.15364365835062	-0.07795061371784
H	-4.20101025487584	2.80673997382837	-1.83424008163587
H	-2.69994395613832	3.12957571363081	-0.91403024898293
H	-1.08128396226037	0.16600084031886	1.85942700968601
H	-1.08955918000130	-1.57837622746538	1.69682405043133
H	0.97142931001258	0.52565357874919	1.85203088069067
H	1.38028834171171	-1.17810623612493	1.88448311631252

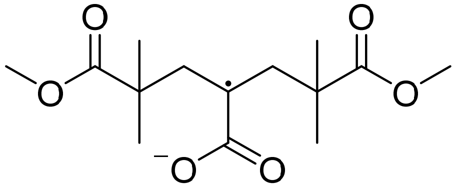

42

C	-2.23209379447477	1.64099463413452	-1.36755432637174
C	-3.91832207987974	1.53088554498389	0.47916120017731
C	0.84503030926352	1.98594594122100	0.26364456929183
C	0.99290054504122	-0.78687168822978	-1.65560178683063
C	1.30315128130895	-2.68889993301939	-0.06451966113402
O	3.53946860175863	-0.43461900557843	-0.88766531811567
C	2.77035234854370	-0.75018459743906	0.16549915978338
O	-3.16578029811150	-1.17671552567870	0.83808344349313
C	-3.28966183844025	-2.58523210604122	0.71084330884053
O	3.21048328352433	-0.85485422168922	1.28422547635123
C	-2.78235718208690	-0.52825772808930	-0.28427672600094
O	-2.63235692810878	-1.11542303492448	-1.32492794070980
O	2.05231584756410	1.66760198011008	0.11279752564216
C	4.75039256217350	0.22607123851370	-0.55214197921085
O	0.33737977153246	3.12396491114396	0.15003429595813
C	-2.59671739830490	0.96214161838851	-0.05227431849278
C	0.36161927414652	-0.55205674518971	0.79682483748327
C	-0.09395719470993	0.86407086092886	0.61863975275140
C	1.35231940050399	-1.16069011694256	-0.22125758946386
C	-1.49143565503107	1.20839600912840	1.01467482377546

H	-3.05082404586744	1.56279339426238	-2.08746457436252
H	-1.99798029462210	2.68888637265662	-1.17573324709872
H	-1.34456637016572	1.18934176446859	-1.80922666899059
H	-4.74248801015455	1.33116190723239	-0.21198727326463
H	-3.81907396289106	2.61290994137233	0.58517881384555
H	-4.17142891686414	1.10836228694081	1.45239481978044
H	1.14682234631889	0.27829092661277	-1.82103854119746
H	1.61483692988455	-1.33599127378138	-2.36587639360357
H	-0.05455443253680	-1.03540471717487	-1.84902445989525
H	2.04935677995424	-3.17317314434512	-0.70053871264282
H	0.31672328457166	-3.05740840619184	-0.36104327545637
H	1.49481444986410	-2.97707599309882	0.97105674137654
H	-2.32782082648942	-3.03201786972622	0.45044850609201
H	-4.01414730897073	-2.84405174658372	-0.06250649515040
H	-3.62254437529375	-2.94658142400655	1.68160686474590
H	4.50678994713246	1.15478420769118	-0.03408359848791
H	5.37935666123800	-0.40182153281405	0.08192427102341
H	5.25081281243644	0.43441060378645	-1.49668730017719
H	-0.51853344900722	-1.20688623009791	0.81368055782971
H	0.82572316140528	-0.65747678108123	1.78814781698632
H	-1.50626942328249	2.28022674357091	1.22282541097980
H	-1.78013681287326	0.66663393457621	1.92421699045023

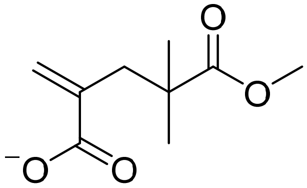

26

C	0.19970143482490	-0.39632153411298	1.57683167038582
C	0.49997434228380	1.99061443497312	0.87998201911263
C	-2.46600033553970	-0.88702957573892	-0.16715663346506
O	-3.61010044025670	-0.71616672049086	0.28855714069738
O	2.29850746509548	-1.04466053431435	-0.15512952161751
C	2.06188435526381	0.26352458469811	0.04916886559429
O	-1.82703955379358	-1.95122553549500	-0.31660392411665
C	3.60835597077038	-1.37341071760463	-0.58574694179196
O	2.93043278913209	1.09079551785501	-0.09206697691210
C	-1.71458875111363	0.38927200439732	-0.63747768339103
C	-0.22829897367693	0.28123686247371	-0.84699129250905
C	-2.38663128923613	1.50507368632855	-0.89376212306055
C	0.61682349930508	0.53679703108156	0.43375642761714
H	0.15689117092250	-1.43322446402132	1.25011952701353
H	-0.80089673663664	-0.12506462019903	1.91695500057359
H	0.88908914365593	-0.29740343253474	2.42160163983011
H	0.83220868237225	2.67400096361082	0.09679839370896
H	1.11080022169303	2.17629780089019	1.76662924896904
H	-0.54165829346542	2.21048114685270	1.11562983351793
H	3.61238153067029	-2.45164683215181	-0.73053372964092
H	4.34823423412776	-1.08747823079927	0.16392507892355
H	3.84810197058112	-0.86394384120984	-1.52037183899972

H	0.10051937874439	0.99858030408267	-1.60821504905049
H	-0.02587655335152	-0.73231433478075	-1.19471238712863
H	-3.45659764364196	1.52821942826732	-0.72190344759018
H	-1.89729961873061	2.39849060794241	-1.27569629667011

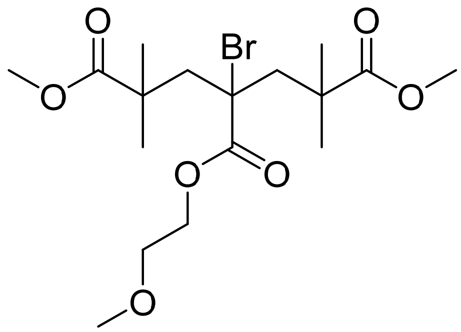

54

C	1.22665638011224	2.95249921702437	-2.27508339848697
C	1.98930687214667	2.13116979671345	-0.04547495949166
Br	-0.88559001204376	0.29364515788156	1.13889202768840
C	1.44277157441621	-0.88200333569509	0.08341131990572
O	-3.88741696229931	-1.37972988824781	0.35654683895522
C	-2.75156941878987	-2.02424606258906	0.07889513939800
C	5.77156713985802	-2.00998572265434	1.44545112493746
O	5.73506803519412	-1.46744949483742	0.14383201048316
C	-0.37609049979131	2.99429726710241	-0.45366534071548
O	-1.46339124736370	3.02132248516501	-0.97110745708726
C	3.48559467852366	-1.81186257869588	-0.64316392317588
O	2.25018337056071	-1.15252022848997	-0.95527374373462
C	-4.50579842270478	-1.74210678475837	1.59066688465582
O	-2.30654162667108	-2.89807450082547	0.77662327102247
O	1.72480739695782	-1.12721179333581	1.21967138704809
O	-0.06119259649973	3.73178304604175	0.61344828585602
C	-1.12984126274475	4.50601835405585	1.15710861446051
C	4.52955049362732	-0.81421725233807	-0.16117134692527
C	0.80369695239616	2.19181760224959	-1.00515685571330
C	-0.59465087042401	-1.54566008934519	-1.08514623483230
C	-2.12727852422658	-1.61131670501458	-1.25687999162160
C	0.10279259060731	-0.32242952746028	-0.42977868970265
C	0.29945195870364	0.80771327231406	-1.45606643634154
C	-2.40661784092642	-2.80443404556014	-2.19041408153911
C	-2.76764605144867	-0.36254664092898	-1.85791634102312
H	0.41204571142248	2.98264022154020	-2.99972789177846
H	1.51078300340779	3.97898472548576	-2.03404999711725
H	2.08757035723230	2.46139738631685	-2.73323873773867
H	1.73346831651062	1.66687310362666	0.90707651276139
H	2.35977230588048	3.13331158950212	0.16773199578407
H	2.80177775715342	1.56526163060131	-0.50752797067563
H	5.00081380486483	-2.77322864850352	1.60001591004986
H	5.63897087840390	-1.22647650606449	2.20046837577867
H	6.75054393590538	-2.46928755072846	1.57249017777364
H	3.80895487286117	-2.27800368499751	-1.57223895987755
H	3.30253699904442	-2.57962327094492	0.10890663832357

H	-3.82662096238787	-1.54660951129032	2.42087403664037
H	-4.76999295012770	-2.79940332203693	1.59126212272178
H	-5.39598158882570	-1.12342695422145	1.66846515027758
H	-0.71181822747732	5.02349702600167	2.01640159894574
H	-1.94746455133990	3.85253948927101	1.46202413439073
H	-1.49979531384655	5.21786511135277	0.41947736041907
H	4.74415266792124	-0.09655769799800	-0.95692341987241
H	4.14588934455942	-0.26607112659859	0.70762573572259
H	-0.13977479824604	-1.64434946965994	-2.07492971472493
H	-0.32006201834209	-2.43925463159600	-0.51999738043811
H	1.01078887638259	0.41523609828347	-2.18623355962145
H	-0.63927834044205	0.96467424461963	-1.98167474071270
H	-2.00190226130338	-2.60216539208433	-3.18401693521443
H	-3.48162958125121	-2.96792052108226	-2.29085086893215
H	-1.95499249187611	-3.71835791071262	-1.80285814243085
H	-2.66023080938184	0.51966319137586	-1.22789677561676
H	-3.83321694752865	-0.52906937936476	-2.01394133612793
H	-2.31867809634357	-0.15319478786488	-2.83168242272986

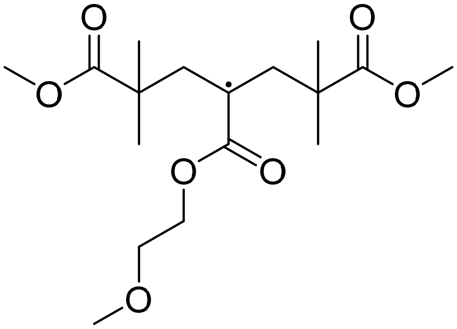

53

C	-2.35277328216597	-0.27509290734024	1.84661118367156
C	-2.10183199547538	-2.43639946756660	0.59806206375401
C	1.07194809910866	-1.53689032709254	-0.33516984979099
O	-0.54727333333263	3.06832253734755	-1.02653991964668
C	-0.24437023869454	2.70062882789579	0.21894451431626
C	6.36536222429705	-0.88404307278040	0.36999511389394
O	5.10659757954041	-1.15028374971227	-0.19149872948530
C	-3.56779512721031	-0.55439032935103	-0.25164135506383
O	-3.73650109359100	0.44734986216396	-0.90018336148766
C	3.19496755526914	-2.54726185137707	-0.33589563599260
O	2.31844342562206	-1.55005330526012	0.18027189106728
C	-1.84886751604204	3.63987853760927	-1.20388338820923
O	-0.97095445131498	2.90834236805669	1.15862237764525
O	0.69477294042109	-2.37023509126407	-1.13358728804691
O	-4.54625790986324	-1.43987541240086	-0.03411949140233
C	-5.81183006013533	-1.11491668052085	-0.60848002991572
C	4.54501587938881	-2.34224698843737	0.29283119181012
C	-2.26511864960286	-0.92440478932682	0.45685995662642
C	0.84713384212630	0.61913177221276	1.03769730997458
C	1.10147073833901	1.98935376497712	0.34657491846210
C	0.25687305728726	-0.43063765114324	0.15284717941568
C	-1.12449660614033	-0.32007379956669	-0.39984378691017

C	1.82292976679386	1.80506560933838	-0.98545102702679
C	1.95589752714187	2.84294301408012	1.29009638128942
H	-2.38781418652322	0.81412867636070	1.78079418222436
H	-3.24350714952546	-0.62744914005874	2.37080484102397
H	-1.48588728310125	-0.55680266553701	2.44661018918798
H	-2.06600719651236	-2.92875947505120	-0.37442824297349
H	-2.91738919072047	-2.86559304865111	1.17880117799604
H	-1.16331188177156	-2.65526574790087	1.11085676562217
H	7.08414012226829	-1.68007432429246	0.13493132490982
H	6.72392656728577	0.05281359039902	-0.05333349657355
H	6.30220678443792	-0.78359350725477	1.46122854723922
H	2.80677660949602	-3.54047582072993	-0.09861756666770
H	3.25586781190970	-2.45268904935448	-1.42178224081995
H	-1.89045500635269	3.95455569549574	-2.24312494899381
H	-1.98672088000404	4.48833018081673	-0.53523876585306
H	-2.61338359349987	2.88690452877414	-1.00289262655083
H	-5.72546047524439	-1.01960501891417	-1.69047325835117
H	-6.18686735208813	-0.17664619337667	-0.19996445249739
H	-6.47401134667755	-1.93666508047004	-0.34862972719027
H	5.18642186622369	-3.20023735372790	0.04258582250067
H	4.44657131156369	-2.30358740221802	1.38723227714860
H	0.18831565102314	0.81011503451610	1.88865821722984
H	1.80264934906500	0.26930672274200	1.42499622034932
H	-1.14195103992246	-0.82360148572433	-1.36863169572294
H	-1.37551330851841	0.72459424854463	-0.57752505369829
H	1.21502414791735	1.25757813309354	-1.70685301971932
H	2.07771367933363	2.76771324832868	-1.43012328057502
H	2.74595928481232	1.24331387295367	-0.82133156190966
H	2.11311123209714	3.84091315039353	0.87472039029237
H	1.47440861805485	2.94817153098044	2.26265073445983
H	2.93232548320643	2.37376382932128	1.42528602896381

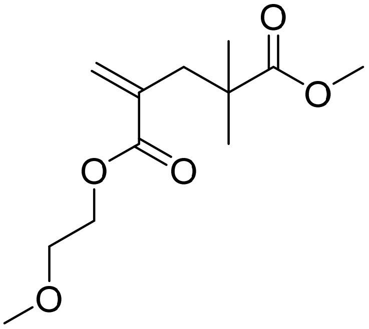

37

C	0.07865724281612	1.45597024890069	-0.40762202476784
C	0.31705070314092	2.15840324623461	-1.50858789000902
C	-1.29806499289771	1.04997352488686	0.03863246432303
C	-1.74485565924883	-0.34356428876796	-0.47683239317606
C	-5.27563198956732	0.04683769171408	0.55927043897295
O	-3.99046139316432	0.29015733476881	-0.01032638882986
C	-3.07755591882335	-0.67449233674574	0.18863203971143
C	1.20725235195022	1.07381192523717	0.49954509772447
O	2.40762700327684	1.18911514308672	-0.07940819753447

O	1.05215397060175	0.71363561577884	1.63985769686399
C	3.54127438146038	0.86106916100274	0.73029230715973
C	3.78981625545081	-0.62522145101802	0.77979411225101
O	4.19694295903367	-1.06658966080528	-0.48927449276300
C	4.39749553908508	-2.45553771991230	-0.53405318378919
O	-3.31960676309721	-1.67432647327288	0.81014834652794
C	-1.96939589935723	-0.31772102304273	-1.99335808186809
C	-0.73765505480319	-1.43359593522759	-0.12075972349217
H	-0.49747317842620	2.48497736088219	-2.14538599353428
H	1.32320417799003	2.42927039162497	-1.79860357692910
H	-2.02068347595941	1.79112706234212	-0.30313925420279
H	-1.31911320232574	1.04468335181507	1.13048045918979
H	-5.87995629508198	0.91578325328141	0.31170643799102
H	-5.19838828581894	-0.07092189590381	1.63986393502904
H	-5.71434082520204	-0.85712193515820	0.13680229374014
H	3.39389794568864	1.24780077031108	1.73898294332371
H	4.38252611160723	1.36286608280688	0.25411443725890
H	4.57288236588818	-0.82289618968879	1.52764610006304
H	2.88010015133249	-1.14813982716420	1.10382316000832
H	3.47644144902311	-2.99967323819263	-0.28836806329672
H	5.18326836660611	-2.76823694083079	0.16619267655433
H	4.70228199814072	-2.71098088854981	-1.54767519402929
H	-2.69417997156517	0.44726555473039	-2.27552009277792
H	-1.02818090645930	-0.11324161494008	-2.50642000311693
H	-2.33672133810044	-1.28489741718153	-2.34366476062235
H	-1.11375285537156	-2.41038697659491	-0.42450402889854
H	0.20665458239551	-1.25653575866139	-0.64180086907425
H	-0.54398855021784	-1.46271114774594	0.95140026601901

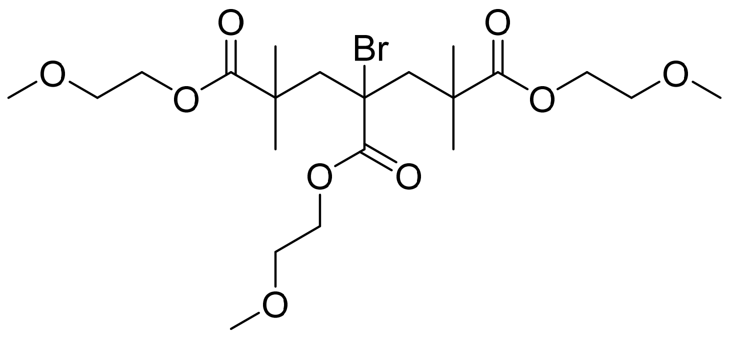

68

C	-3.14404521070248	3.78917698122021	1.12991525830898
C	-2.39942136176230	5.02921377688535	0.66823018826250
C	4.24139355210672	1.78019363678559	-0.66840274446868
O	3.48622875280359	0.56837577872732	-0.72599261486603
O	-1.60945695580472	4.81597939805788	-0.47136204537990
C	-2.33521483564476	4.73191035409380	-1.67881352813148
C	2.61661517881493	0.46417315877487	-1.74441604400593
C	-0.72694594648969	0.99401147235614	0.76468211591863
C	-1.99128024215862	1.82025392857247	0.49330391768350
O	-2.19821068036392	2.76001537488866	1.42662468625639
C	-0.54194047292134	-2.73603212070954	-0.69506855735994

Br	0.05669900718038	-2.36610531272848	1.91893884416763
C	1.84741680621855	-0.85757420979054	-1.73067626402879
C	-2.44770667077692	-3.88523447180115	-1.43023082062883
O	-1.86807393911850	-2.75605916864847	-0.76608311562679
C	6.98186589800038	2.94630401887177	0.12273145605520
O	5.76590459522265	2.91203653960500	0.83067351199954
C	5.02124436830578	1.73814544283344	0.63351427363080
C	-5.74447218173612	-3.78128823802999	0.12638788230262
O	-4.36488004183109	-3.98226331728104	-0.04170327291389
O	-2.68995325099518	1.68770848948615	-0.47824524446872
O	2.51811237989460	1.30914575774179	-2.59343264882601
C	-3.94259683158291	-3.74689180765931	-1.35747518306324
O	0.16418808573472	-3.57568915078495	-1.18799312238047
C	1.45962843931002	-1.27504015046592	-0.26811438252580
C	2.84573316684834	-1.90263912176818	-2.27013856201628
C	0.70775686379731	-0.72597167125291	-2.74380223011711
C	-1.01254346853684	-0.37851363438173	0.10898008519875
C	-0.00628065983516	-1.53808923392113	0.10704126737564
C	-0.41943600395785	0.92199238441247	2.26015400165993
C	0.37398626953104	1.81562966746877	0.06779756237089
H	-3.85384168608483	3.43012920219483	0.38565939036862
H	-3.67339310449658	4.00146893846169	2.05956414915423
H	-3.12864046238791	5.83506673159612	0.49833468905137
H	-1.71422303667177	5.34888652572660	1.45592622706458
H	4.89197971981097	1.84266840697374	-1.54308047611247
H	3.56729089756308	2.63885680145400	-0.68636459780510
H	-2.97075495312377	3.84141888491603	-1.71047317503410
H	-1.60393024404324	4.66720497236543	-2.48282980113236
H	-2.95036761286663	5.62875345816479	-1.82585738324552
H	-2.11543001357903	-4.80048962075278	-0.93821677880239
H	-2.10902835153528	-3.90372422055446	-2.46807525652138
H	6.83135236003702	2.97222937992809	-0.96255674596885
H	7.60611000272184	2.07865615458365	0.37058516293352
H	7.50274175527390	3.85418546197346	0.42274776961021
H	4.32117931626252	1.66160648576301	1.46818657116149
H	5.66885204435326	0.85115595225362	0.65380756169486
H	-6.32850511135660	-4.44823056436189	-0.52167645805183
H	-6.02507179427197	-2.74415453689304	-0.09692774210180
H	-5.98244146265563	-3.99905211017155	1.16609040249600
H	-4.23941056914895	-2.74095752124918	-1.68698595548828
H	-4.39631967933189	-4.47501731532457	-2.04647928881556
H	1.98659862786662	-2.20290763635099	-0.05483228756945
H	1.85006484125702	-0.55026489939147	0.44107192742220
H	3.70800995637110	-1.99596258608829	-1.60808484261351
H	3.19866745302488	-1.62025274839794	-3.26429228598155
H	2.34844288062267	-2.87097572308844	-2.33145258046440
H	0.00681634209753	0.06710872402472	-2.48999426540578
H	1.12623295859594	-0.47265691338435	-3.71672407456622
H	0.16939186716512	-1.66703389299797	-2.84680884024415
H	-1.27077975125977	-0.18246999160056	-0.93098622098816
H	-1.93090560160524	-0.76600452410305	0.55071215101021

H	-0.33093248233739	1.92439961010690	2.67568764991257
H	-1.19493048402920	0.38729258942967	2.81056867860499
H	0.52389855914836	0.40228598143481	2.42439537938808
H	0.31273738532509	2.85957083751222	0.38138246478346
H	0.27259173512180	1.79556955994215	-1.01789805401353
H	1.36202308861631	1.45057059434662	0.34454026588695

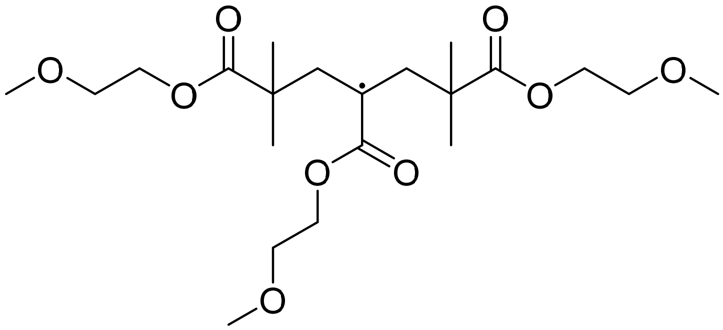

67

C	3.97383028112061	3.50986530871762	-0.28484784105437
C	5.34381748689295	3.66380840016904	-0.90638607145074
C	-5.71321501018622	0.73735574572908	0.07839811357158
O	-4.37373320437510	0.35563264422303	-0.24501442601006
O	6.38982248375070	3.15350506707499	-0.11990295626999
C	6.74691592040153	3.97337651522087	0.96502359190781
C	-3.47762283720069	0.40129175587481	0.75001763937682
C	2.34372633634468	0.26024406202576	0.45698479937797
C	2.54794288971723	1.78096558816192	0.42462150016881
O	3.68992861736170	2.11336869576573	-0.18866430709734
C	0.40998500232351	-2.36608932253975	0.85058336017630
C	-2.04785364427946	0.16864600052651	0.26800306959847
C	-0.46561616584229	-4.48196998896118	0.25541971628008
O	-0.68799571888356	-3.11894621119514	0.61622210273586
C	-6.47735157498749	2.64002179357590	-2.17634665895791
O	-5.53431872714779	2.84777114994403	-1.15533517477099
C	-5.86750846362782	2.24607051556712	0.07053946007365
C	1.26900204000443	-6.38034828298074	-1.48628280907449
O	-0.05505545025439	-5.92302904237065	-1.64428823913327
O	1.78496775138384	2.60856999362538	0.85451004329282
O	-3.77555038624478	0.65031867850924	1.88979586960773
C	-0.24143271420983	-4.58825079292349	-1.24645063021496
O	1.53332293311538	-2.82797785505199	0.80732759901450
C	-1.31519014330241	-0.56631525530534	1.42196444849910
C	-1.47174472000768	1.57373056353120	0.03845593494915
C	-2.00915920430743	-0.63820943542475	-1.02852897586557
C	1.22810600358601	-0.05224311017961	1.50417188843733
C	0.09985734146401	-0.97859739635245	1.16438625285675
C	3.66044495959698	-0.42038184122929	0.85089853003434
C	1.98206508063675	-0.14736748245159	-0.97980480924943
H	3.91556668184744	3.95804404935362	0.70877390368406
H	3.21941364400512	3.99543104159666	-0.90921654869689
H	5.37484411220617	3.11660885325440	-1.85039143987409
H	5.50647754906487	4.72974267476705	-1.12311786102491

H	-6.33178403446458	0.26110629971838	-0.68172137540687
H	-5.98563574191709	0.34945298885856	1.06079635742576
H	7.04071216716935	4.97488438780516	0.62415981535216
H	7.59728527828186	3.50310176181118	1.45602840180537
H	5.93684396711213	4.07671579457524	1.69654540110564
H	0.38605566189477	-4.87562625276214	0.81021102226682
H	-1.36972190503735	-5.01833019389367	0.54039480828607
H	-6.16901241059421	3.24526298541239	-3.02747495853173
H	-6.52517486265702	1.59206650782072	-2.49506427900160
H	-7.47997719482291	2.95600717523931	-1.85927120742282
H	-6.90263560219491	2.48890616478643	0.35355238123127
H	-5.20075483027367	2.67867234089199	0.81764461526565
H	1.96461923278619	-5.77716551320937	-2.08187078876310
H	1.59524568981980	-6.35570106329599	-0.44060659145407
H	1.29713288441236	-7.41006660081884	-1.83917937761239
H	0.61807656287514	-3.96778184148077	-1.53304315865706
H	-1.12379447464340	-4.21309192895149	-1.76971884864453
H	-1.90141593971866	-1.44966601265218	1.67641111620359
H	-1.33517852590069	0.09313498979543	2.29289659418241
H	-2.05435551122213	2.10384305955178	-0.71855551836886
H	-0.43857744591804	1.52374151605133	-0.30373433062105
H	-1.48497999009224	2.15917701586474	0.95914860423166
H	-0.97298032000970	-0.79843931336452	-1.33307553610715
H	-2.47619664499349	-1.61621135517946	-0.90577522062241
H	-2.52278464361294	-0.10958859595899	-1.83022850302143
H	1.72712409286306	-0.47508580263301	2.38506459533192
H	0.79163017455510	0.89282187411291	1.82228089682107
H	3.49752491164625	-1.49544159639187	0.90950657948988
H	4.44399150742411	-0.21133668533135	0.12393815764776
H	4.00478546991802	-0.06749309879555	1.82616591721701
H	1.04154565677457	0.29675078096113	-1.31073034988513
H	2.76735338338711	0.17404115940462	-1.66488494483657
H	1.90080328718624	-1.23165902819007	-1.04789634980541

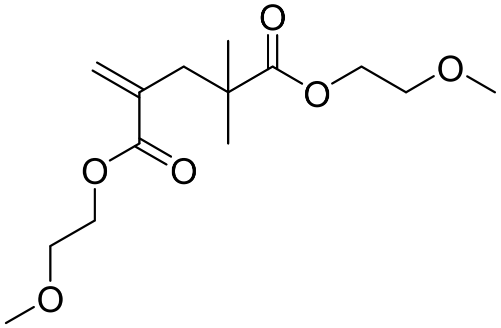

44

C	-2.04715522558498	1.59380705535342	-0.94258649190742
C	-1.61559635876933	2.33719103819186	1.44862405155424
C	-2.00984520350388	-0.03779573902554	0.90961533120676
O	-3.34155984050903	0.10714450052448	1.03929683787655
C	5.40403721174747	-3.49708946451990	0.43133845640375
O	5.30037161296431	-2.15267850404240	0.03841688772672

C	4.02450763628242	-0.20930464975636	-0.27818694575802
O	2.69194691269226	0.28578212035687	-0.13714241604104
C	-4.66530443846544	-1.90073710310235	-2.22897259977777
O	-4.03007241037021	-1.74295548919837	-0.98657801624216
C	-4.12335876585475	-1.06334415740199	1.27744274742960
O	-1.42340363429582	-1.04953196027432	1.17933952982481
C	2.50026336753807	1.56295891440285	-0.49444485562349
C	3.99718588218487	-1.65084464295052	0.17235967446507
C	-4.91841045637297	-1.37904482946708	0.03596789815225
O	3.38497907806138	2.26165460366281	-0.91384252991311
C	-1.36559279539207	1.25292485340897	0.39011002981870
C	0.12541699948594	0.93870461273976	0.20854381743955
C	1.06889838780356	1.99611333365822	-0.30714233126337
C	0.82690270608851	3.26260087863958	-0.62177655628140
H	-1.95529095595663	0.76034765455982	-1.64326997673677
H	-3.10786553433197	1.79144746983809	-0.79199489941993
H	-1.59576934208220	2.46924535720451	-1.40591724079534
H	-1.20941733140158	3.29859092052000	1.14118329634875
H	-2.68435167214091	2.46152914950114	1.62200943433904
H	-1.14349478650165	2.06610639008087	2.39587209549828
H	6.43902195357943	-3.80191787165785	0.28531852483378
H	4.75172089087683	-4.13965050194084	-0.17381032947406
H	5.13737710220500	-3.62551132590359	1.48815952093758
H	4.34398912816857	-0.12791185083582	-1.31816263895007
H	4.70625206818106	0.38227068427505	0.33466875969840
H	-5.43724064491865	-2.67997067683767	-2.18732255780287
H	-3.90500346249689	-2.19351944680223	-2.95109826028962
H	-5.13217856591470	-0.96369882025397	-2.55932303190990
H	-3.46899682432549	-1.89261857038644	1.54342989125225
H	-4.79456374152562	-0.84005123874152	2.10782882560506
H	3.65717751947001	-1.71265776270684	1.21567041863438
H	3.29061311806040	-2.22531422675608	-0.44313305749462
H	-5.62210662671963	-2.19635685759291	0.25420934517731
H	-5.50569456843838	-0.49824723254224	-0.26232319470909
H	0.51872028066798	0.57570962523853	1.16137174607004
H	0.20905744769746	0.07088630035755	-0.45025961802315
H	-0.14854151790031	3.71834939323715	-0.54279694874879
H	1.64156940001755	3.88028206694533	-0.97825262313089

References:

(1) Kapil, K.; Jazani, A. M.; Szczepaniak, G.; Murata, H.; Olszewski, M.; Matyjaszewski, K. Fully Oxygen-Tolerant Visible-Light-Induced ATRP of Acrylates in Water: Toward Synthesis of Protein-Polymer Hybrids. *Macromolecules* **2023**, *56*, 2017-2026.

(2) Szczepaniak, G.; Jeong, J.; Kapil, K.; Dadashi-Silab, S.; Yerneni, S. S.; Ratajczyk, P.; Lathwal, S.; Schild, D. J.; Das, S. R.; Matyjaszewski, K. Open-air green-light-driven ATRP enabled by dual photoredox/copper catalysis. *Chemical Science* **2022**, *13*, 11540-11550.

(3) Carmali, S.; Murata, H.; Cummings, C.; Matyjaszewski, K.; Russell, A. J. Polymer-Based Protein Engineering: Synthesis and Characterization of Armored, High Graft Density Polymer-Protein Conjugates. In *Nanoarmoring of Enzymes: Rational Design of Polymer-Wrapped Enzymes*, Kumar, C. V. Ed.; Methods in Enzymology **2017**, *590*, 347-380.

(4) Murata, H.; Carmali, S.; Baker, S. L.; Matyjaszewski, K.; Russell, A. J. Solid-phase synthesis of protein-polymers on reversible immobilization supports. *Nature Communications* **2018**, *9*, 845.

(5) Li, F.; Cao, M.; Feng, Y.; Liang, R.; Fu, X.; Zhong, M. Site-Specifically Initiated Controlled/Living Branching Radical Polymerization: A Synthetic Route toward Hierarchically Branched Architectures. *Journal of the American Chemical Society* **2019**, *141*, 794-799.

(6) Fantin, M.; Lorandi, F.; Ribelli, T. G.; Szczepaniak, G.; Enciso, A. E.; Fliedel, C.; Thevenin, L.; Isse, A. A.; Poli, R.; Matyjaszewski, K. Impact of Organometallic Intermediates on Copper-Catalyzed Atom Transfer Radical Polymerization. *Macromolecules* **2019**, *52*, 4079-4090.

(7) Fang, C.; Fantin, M.; Pan, X. C.; de Fiebre, K.; Coote, M. L.; Matyjaszewski, K.; Liu, P. Mechanistically Guided Predictive Models for Ligand and Initiator Effects in Copper-Catalyzed Atom Transfer Radical Polymerization (Cu-ATRP). *Journal of the American Chemical Society* **2019**, *141*, 7486-7497.

(8) Sobieski, J.; Silab, S. D.; Thevenin, L.; Matyjaszewski, K.; Fliedel, C.; Poli, R. Termination of the Carbomethoxyisopropyl Radical, a Poly(methyl methacrylate) Model, in the Presence of Copper Complexes and Proton Donors. *Macromolecules* **2023**, *56*, 6339-6353.
